# Blood Cells and Venous Thromboembolism Risk: A Two-Sample Mendelian Randomization Study

**DOI:** 10.3389/fcvm.2022.919640

**Published:** 2022-07-08

**Authors:** Jiahao He, Qian Jiang, Yiting Yao, Yi Shen, Juan Li, Jianuo Yang, Ran Ma, Nuofu Zhang, Chunli Liu

**Affiliations:** State Key Laboratory of Respiratory Disease, National Center for Respiratory Medicine, National Clinical Research Center for Respiratory Disease, Guangzhou Institute of Respiratory Health, The First Affiliated Hospital of Guangzhou Medical University, Guangzhou, China

**Keywords:** Mendelian randomization analysis, venous thromboembolism, blood cells, mean red blood cell volume, red blood cell distribution width, mean reticulocyte volume, monocyte count

## Abstract

**Background:**

Previous studies have shown that various cell indices are associated with a higher risk of venous thromboembolism (VTE), however, whether these findings reflect a causal relationship remains unclear. Therefore, we performed a two-sample Mendelian randomization (MR) analysis to assess the causal association of various blood cells with VTE risk.

**Study Design and Methods:**

Summary statistics of genetic instruments representing cell indices for erythrocytes, leukocytes, and platelets were extracted from genome-wide association studies of European ancestry, by Two-Sample Mendelian Randomization. Inverse variance weighting (IVW) was used as the primary analytical method for MR. Sensitivity analyses were performed to detect horizontal pleiotropy and heterogeneity.

**Results:**

Genetically predicted red blood cell distribution width, mean reticulocyte volume, and mean red blood cell volume were positively associated with VTE, with odds ratio (OR) of 1.002 [CI 1.000–1.003, *P* = 0.022), 1.003 (CI 1.001–1.004, *P* = 0.001, respectively)] and 1.001 (CI 1.000–1.002, *P* = 0.005). Genetically predicted monocyte count was negatively correlated with VTE, with OR = 0.998 (CI 0.996–0.999, *P* = 0.041).

**Conclusion:**

Genetically liability to high- red blood cell distribution width, mean reticulocyte volume, mean red blood cell volume, and low monocyte count are associated with the higher risk of VTE. Targeting these factors might be a potential strategy to prevent VTE.

## Introduction

Venous thromboembolism including deep vein thrombosis and pulmonary embolism, the third most common vascular disease, is currently one of the leading causes of unanticipated and perioperative deaths in hospitals, affecting nearly 1 million people worldwide each year (1, 2). VTE is affected by a variety of environmental and genetic factors (3). Studies have shown that blood cells such as red blood cells, white blood cells and platelets are associated with the risk of VTE (4–9). In addition, pathology suggests that the embolic components of VTE patients include red blood cells, platelets and white blood cells (10, 11). However, the causal relationship between blood cells and the risk of VTE remains unclear. The occurrence of VTE and changes in cell indices are affected by a variety of factors, such as immobilization, infection, surgery and disease; in addition, if cell indices changes are secondary to the occurrence of VTE or a prothrombotic state, there may also be reverse causality. Evidence from previous observational studies may be influenced by confounding factors or reverse causality.

Mendelian randomization (MR) is an epidemiological method that uses genetic variation as an instrumental variable for exposure to estimate the causal effect of exposure on outcomes and strengthens causal inference, overcoming the limitations of traditional observational study designs by using Mendelian laws, exploits the random assignment to genotypes at conception, making genotypes independent of potential confounders and also avoiding reverse causality (12). Two-sample MR analysis is an extension of the MR approach that allows summary statistics from genome-wide association studies (GWAS) to be used in MR studies without direct analysis of individual-level data (13). In recent years, two-sample MR studies have gradually been recognized by researchers, allowing data between genetic instrument variables and phenotypes, phenotypes and diseases to come from two different independent populations, improving the efficiency and statistical power of the study (14–16). Through the statistical method of two-sample MR, this study used the genome-wide association analysis (GWAS) database to select single nucleotide polymorphism (SNP) loci as instrumental variables to explore the relationship between blood cells (erythrocytes, leukocytes, and platelets) indices and VTE.

## Materials and Methods

### Study Design

**FIGURE 1:**
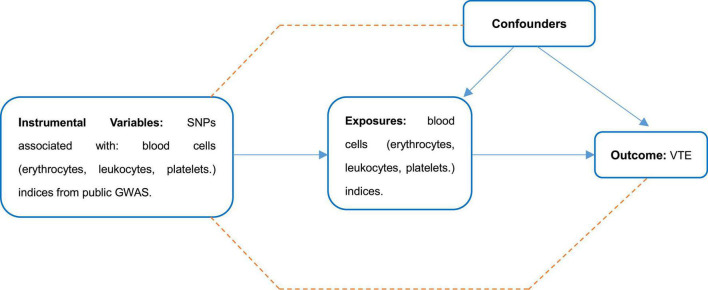
The scheme of study design.

A schematic diagram of the study design and the three hypotheses of MR are presented in Figure 1. Genetic variants associated with cell indices are used as instrumental variables (IVs) to assess causal associations with VTE risk. There are three main assumptions that need to be met to perform an MR analysis: First, there is a robust correlation between these instrumental variables and exposure factors (association hypothesis); second, the instrumental variables are independent of the confounding factors that affect the “exposure-outcome” relationship (independence assumption). Finally, genetic variation affects outcomes only through exposure, not through other pathways (exclusivity hypothesis). Genetic associations between instrumental variables and traits were adjusted for age, sex, and study-specific covariates included in the GWAS. We performed a secondary analysis of publicly available pooled data. This manuscript did not produce any raw data. Ethical approval for each study used and informed consent from subjects were provided in the original publication. Secondary analyses of aggregated data do not require an Institutional Review Board. This study followed the ethical guidelines of the 1975 Declaration of Helsinki.

### Data Source and Single Nucleotide Polymorphism Selection

Summary level data of the cell indices which related to red blood cells, white blood cells and platelets from the Neale lab analysis of UK Biobank phenotypes, round 2^1^, 4,620 patients and 356,574 controls with European-ancestry were included, of whom 53.76% were female and 46.24% were male. Summary level data of the cell indices which related to neutrophils, eosinophils, and basophils from the datasets that satisfy minimum requirements imported from the EBI database of complete GWAS summary data^2^. Summary level data of the cell indices which related to neutrophils, eosinophils, and basophils from the datasets that satisfy minimum requirements imported from the EBI database of complete GWAS summary data (see text footnote 2) which involved 173,480 European-ancestry participants. Detailed information of data source for the instrumental variables associated with exposures are shown in Supplementary Tables 1, 2. Single-nucleotide polymorphisms (SNPs) were identified as associated with the exposures with *p*-values at the genome-wide significance level (*p* < 5 × 10^–8^). SNPs with *R*^2^ > 0.01 and within 5,000 kb distance were identified as in linkage disequilibrium and were excluded from the study. Associations of these SNPs with VTE were studied in summary level results including 4,620 patients and 356,574 controls from Neale lab analysis of UK Biobank phenotypes, round 2 (see text footnote 1), 53.76% are female and 46.24% are male. The study design like the collection of samples, quality control procedures and imputation methods have been described in the original publication. Genotype imputation and associated quality control procedures have been previously described (17, 18).

### Statistical Methods

The multiplicative random-effects (RE) and fixed-effects (FE), inverse-variance weighted (IVW) were used to assess the causal associations between the exposures and VTE. The effect measures were the odds ratio (OR) of the risk of VTE, which was normalized to one SD increment in each exposure factor. In addition, we conducted weighted median (WM)-based method and MR-Egger statistical sensitivity analyses to ensure the robustness of pleiotropic IVs (19). MR-Egger method can identify potential pleiotropy (*p* for intercept <0.05) and give corrected estimates (20). Heterogeneity was assessed with the *I*^2^ index (21) and the funnel plot (22). We used leave-one-out analysis to evaluate the stability of these genetic variants by excluding one individual SNP each time. All statistical results are two-sided, and a *p* < 0.05 was considered statistically significant. The statistical analyses were performed with R (version 4.0.4), TwoSampleMR (0.5.5), and MR (0.5.0) (23).

## Results

Mendelian randomization analysis showed that red blood cell distribution width (RDW), mean corpuscular volume of reticulocyte (MCVr), mean corpuscular volume (MCV), and monocyte count (MONO) had a significant causal relationship to VTE (Table 1). All MR results are shown in Supplementary Table 2. The results showed that genetically predicted RDW, MCVr, and MCV were positively correlated with VTE but monocyte count was negatively correlated with VTE. RDW, MCVr, and MONO used the multiplicative random-effects model IVW because of large heterogeneity, while MCV used fixed-effects model IVW (Figure 2 and Table 1). Heterogeneity test results are the same as funnel plot results (Supplementary Figures 1–23). The MR-Egger method did not find horizontal pleiotropy (RDW: *P* = 0.279, MCVr: *P* = 0.0701, MCV: *P* = 0.400, MONO: *P* = 0.246), and the Leave-one-out sensitivity test showed all independent SNP could drive a significant effect of RDW on VTE. The significant associations of RDW and VTE did not remain after removing move one of them, but in MCVr, MCV, and MONO, we did not find any independent SNP could drive a significant effect on VTE (Supplementary Figures 24–46). We found no causal relationship between other cell indices and VTE (Supplementary Figures 47–69). Scatter plot showing the relationship between 23 exposure factors and VTE (Supplementary Figures 70–92).

**TABLE 1:** Mendelian randomization estimates for the causal effect of cell indices on VTE.

Exposures	Methods	OR (95% CI)	*P*-value	Cochrane’s *Q* value	Intercept of pleiotropy	*p* for pleiotropy
**Mean corpuscular volume**						
	IVW (FE)	1.001 (1.0004, 1.0022)	0.005	520.085	3.12356E-05	0.400
	IVW (RE)	1.001 (1.0002, 1.0024)	0.024			
	Weighted median	1.001 (9998, 1.0031)	0.092			
	MR-Egger	1.001 (0.9988, 1.0025)	0.493			
**Monocyte count**						
	IVW (FE)	0.998 (0.9971, 0.9993)	0.001	745.344	5.76487E-05	0.227
	IVW (RE)	0.998 (0.9965, 0.9999)	0.041			
	Weighted median	0.998 (0.9966, 1.0004)	0.122			
	MR-Egger	0.997 (0.9939, 0.9997)	0.029			
**Mean corpuscular volume of reticulocyte**						
	IVW (FE)	1.003 (1.0015, 1.0036)	0.000	633.335	9.19964E-05	0.070
	IVW (RE)	1.003 (1.0010,1.0041)	0.001			
	Weighted median	1.001 (0.9997, 1.0032)	0.106			
	MR-Egger	1.000 (0.9977, 1.0032)	0.752			
**Red blood cell (erythrocyte) distribution width**						
	IVW (FE)	1.002 (1.0008, 1.0030)	0.001	574.301	0.000	0.279
	IVW (RE)	1.002 (1.0003, 1.0035)	0.022			
	Weighted median	1.000 (0.9980, 1.0019)	0.958			
	MR-Egger	1.001 (0.9978, 1.0034)	0.674			

*OR, odds ratio; CI, confidence interval; IVW, inverse variance weighted; RE, random-effects; FE, fixed-effects.*

**FIGURE 2:**
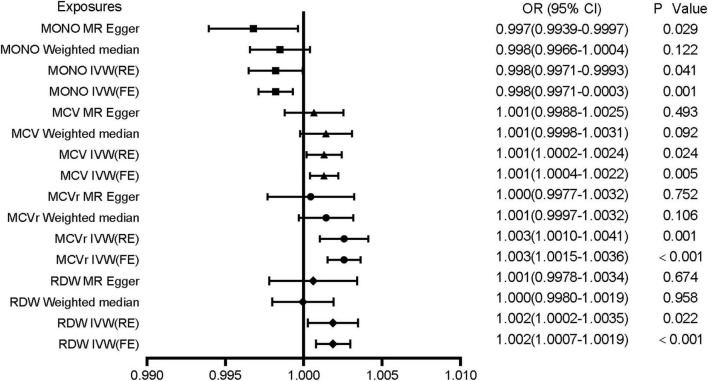
Associations of genetically predicted MONO, MCV, MCVr, and RDW with VTE. MONO, monocyte count; MCV, mean corpuscular volume; MCVr, mean corpuscular volume of reticulocyte; RDW, red blood cell distribution width; OR, odds ratio; CI, confidence interval.

## Discussion

In this two-sample Mendelian randomization analysis, we investigated the association of blood cell indices with VTE risk. Our results show that genetically proxied red blood cell distribution width (RDW), mean corpuscular volume of reticulocyte (MCVr), and mean red blood cell volume (MCV) are positively correlated with VTE, and monocyte levels are negatively correlated with VTE. There was no evidence that other blood cell-level characteristics were causally related to VTE risk.

Previous cohort studies have shown that high RDW levels are a risk factor for VTE events, and RDW is a predictor of all-cause mortality in VTE patients (24, 25). High levels of RDW are associated with both premature release of immature red blood cells into the blood (25) and disease-related biological and metabolic imbalances (26, 27). Furthermore, RDW is negatively correlated with erythrocyte deformability, and high RDW levels may lead to cell aggregation and increased blood viscosity (4, 28), which in turn promotes thrombosis. We demonstrated a causal relationship between RDW and VTE by MR analysis based on GWAS, a relatively large population. Therefore, although the mechanism of the association between RDW and VTE remains unclear, it seems to be explained by the intermediate development of other diseases and the effect of RDW on hemodynamics, and future studies are necessary to explore the specific mechanism. The reason why the MR results of RDW are not robust may be related to the constant changes in red blood cell volume and traits. Hematocrit, hemoglobin concentration, and red blood cell count were not associated with an increased risk of VTE in a prospective cohort study (29). However, another prospective study involving 26,108 subjects with a median follow-up of 12.5 years found that hematocrit, hemoglobin concentration, red blood cell count, except MCV were risk factors for VTE in the general population (30). They believe that the effect of blood cells on VTE is mainly achieved by changing the blood viscosity by affecting the hematocrit, which were confirmed by other studies (31, 32). It is worth noting that a prospective cohort study of 108,521 subjects found no association between high hematocrit and VTE risk (33). Conflicting results may be biased by uncharacterized confounding factors in observational studies, as characteristics such as blood cell levels and size are influenced by numerous factors, including disease, external and internal environments. In addition, our MR analysis showed a causal relationship between VTE and erythrocyte volume-related cell indices such as MCVr and MCV. The possible reason for this result is that although red blood cells are an important component of blood clots, they are more likely to be captured by fibrin due to their larger volume in the flow of white blood clots, it is not the function of red blood cells that plays a major role in the thrombosis process. A retrospective study of 5408 subjects found a strong dose-response relationship (OR 2.8, 95% CI 1.3–5.8) between MONO and VTE (24), and it is now believed that monocytes was involved in thrombosis (34) and thrombolysis (35) through tissue factor expression, which is consistent with the results of our MR analysis, that the absolute level of monocytes was causally related to VTE, while the percentage of monocytes is not.

A prospective cohort study of 2,430 subjects found no association between WBC counts and VTE (OR 0.94, 95% CI 0.65–1.36) (36), whereas another prospective cohort study of 19,237 subjects with a median follow-up of 7.8 years concluded the opposite (37). Our MR analysis showed no evidence of a causal relationship between indices of other leukocyte lineages and VTE other than monocyte counts. However, some studies have also thought that neutrophils, lymphocytes, eosinophils and VTE are related (38–42), which studies focus on hospitalized patients and are subject to bias due to confounding factors such as immobilization, medication use, surgical intervention, and the disease itself. We speculate that the neutrophil-VTE connection is primarily through neutrophil extracellular traps rather than neutrophil counts, and that NETs are able to directly activate coagulation, bridging the immune and coagulation systems through immunothrombosis. For this reason, dipyridamole is clinically used to inhibit NETosis in order to prevent thrombosis. The most well-defined roles for monocytes in coagulation are to initiate coagulation through presentation of TF and to potentiate thrombo-inflammation through inflammasome activation, which is largely related to the number of monocytes. We believe that the function of neutrophils is closely related to VTE and the number of monocytes is related to VTE. For the above reasons, more research is necessary.

Platelets are the predominant cell type for thrombosis, and high platelet counts may be associated with susceptibility to thrombosis, but our MR showed that genetically proxied platelet counts are not associated with VTE, which is consistent with the results of a prospective cohort study of 108,521 subjects with a median follow-up of 8 years in Hagen (33) and a prospective study with a median follow-up of 12.5 years (29). High platelet count levels in healthy individuals are not a susceptibility factor for VTE, platelet count changes may be secondary to other high-risk factors for VTE, and genetically proxied platelet counts are not associated with VTE risk. For platelets, many studies have demonstrated that mean platelet volume (MPV), a measure of platelet activity, is associated with VTE (43–48), but our results were negative. The lifespan of platelets is about 1 week, so PLT, MPV, and PDW are prone to major changes due to platelet turnover. The variable shape and volume of platelets means that platelet parameters should not be used alone as indicators of platelet activation even in normal populations, maybe association of platelets with VTE may be caused by other confounding factors.

This study has several limitations. First, we assumed that the association of different exposures with VTE was linear in the MR analysis, but that Developmental adaptation could alter the effect of the genetic instruments on the outcome (49). Second, we were unable to stratify populations so that conclusions might be compromised if allele frequencies differ across populations. Third, the results are based on European ancestry, and future studies in mixed or other populations are warranted to extend our conclusions. The OR is not very significant, which suggests that genetically predicted cell indices are not the main factor affecting the occurrence of VTE, but our results provide a certain reference for further research. Finally, our method could not demonstrate confounding factors in the MR analysis of the relationship between various cellular components and VTE, nor could it demonstrate the interaction relationship between blood cell components and VTE.

It’s worth noting that our results are less susceptible to biases including confounding factors and reverse causality, which is particularly important in disease conditions square up the large variability in circulating metabolites and blood cells within the human microenvironment.

## Conclusion

Our MR study demonstrated the causal effects of genetically proxied RDW, MCVr, MCV, and MONO on the risk of VTE. Blood cells affect the occurrence of VTE to a certain extent. Targeting these factors might be a potential strategy to prevent VTE, Future studies are needed to explore the exact mechanism and confirm the potential clinical value of such a prevention and treatment strategy.

## Data Availability Statement

The original contributions presented in this study are included in the article/Supplementary Material, further inquiries can be directed to the corresponding author/s.

## Author Contributions

JH had full access to all of the data in the study and took responsibility for the integrity of the data and accuracy of the data analysis, including and especially any adverse effects. QJ, YY, JL, YS, JY, RM, NZ, and CL contributed substantially to the study design, data analysis, and interpretation, and writing of the manuscript. All authors made substantial contributions to the conception and design, acquisition of data, and analysis and interpretation of data.

## Conflict of Interest

The authors declare that the research was conducted in the absence of any commercial or financial relationships that could be construed as a potential conflict of interest.

## Publisher’s Note

All claims expressed in this article are solely those of the authors and do not necessarily represent those of their affiliated organizations, or those of the publisher, the editors and the reviewers. Any product that may be evaluated in this article, or claim that may be made by its manufacturer, is not guaranteed or endorsed by the publisher.

## Footnotes

1
http://www.nealelab.is/uk-biobank

2
https://www.ebi.ac.uk/gwas

## Supplementary Material

Supplementary Figures 1–23 Funnel plot showing results of heterogeneity test.

Supplementary Figures 24–46 Leave-one-out analysis of cell indices.

Supplementary Figures 47–69 Associations of 23 exposures with venous thromboembolism (VTE) in Mendelian randomization analyses.

Supplementary Figures 70–92 Scatter plot shows individual causal estimates from each genetic variant associated with each exposure on the *x*-axis and VTE on the *y*-axis.

Supplementary Table 1 Single-nucleotide polymorphisms used for each exposure.

Supplementary Table 2 Results of MR analysis.
