# Supplementary material for: Blood Cells and Venous Thromboembolism Risk: A Two-Sample Mendelian Randomization Study
Source: Front Cardiovasc Med. 2022 Jul 8;9:919640. doi: 10.3389/fcvm.2022.919640 (PMC9304581; doi:10.3389/fcvm.2022.919640)

Supplementary figure 1: Funnel plot to assess heterogeneity of white blood cell count. The blue line represents the inverse variance weighted estimate, and the dark blue line represents the Mendelian Randomization Egger estimate.

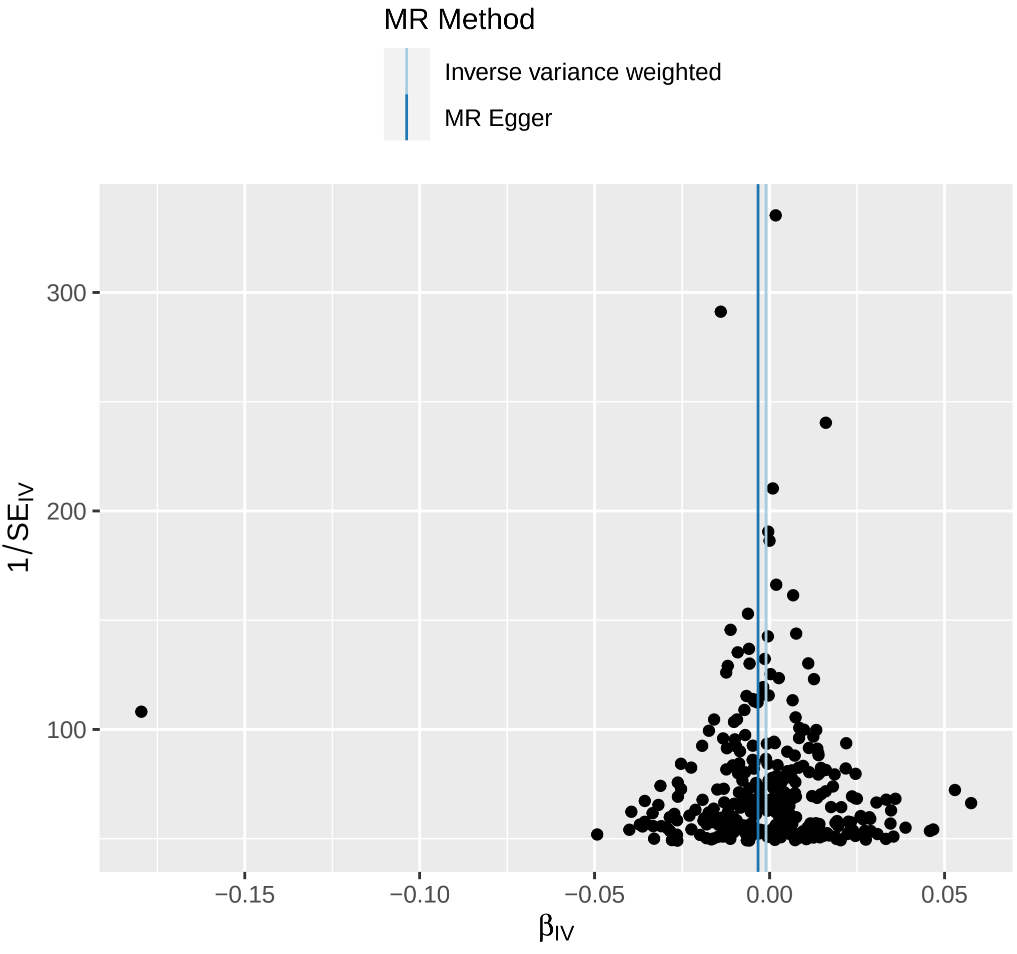

Supplementary figure 2: Funnel plot to assess heterogeneity of monocyte percentage. The blue line represents the inverse variance weighted estimate, and the dark blue line represents the Mendelian Randomization Egger estimate.

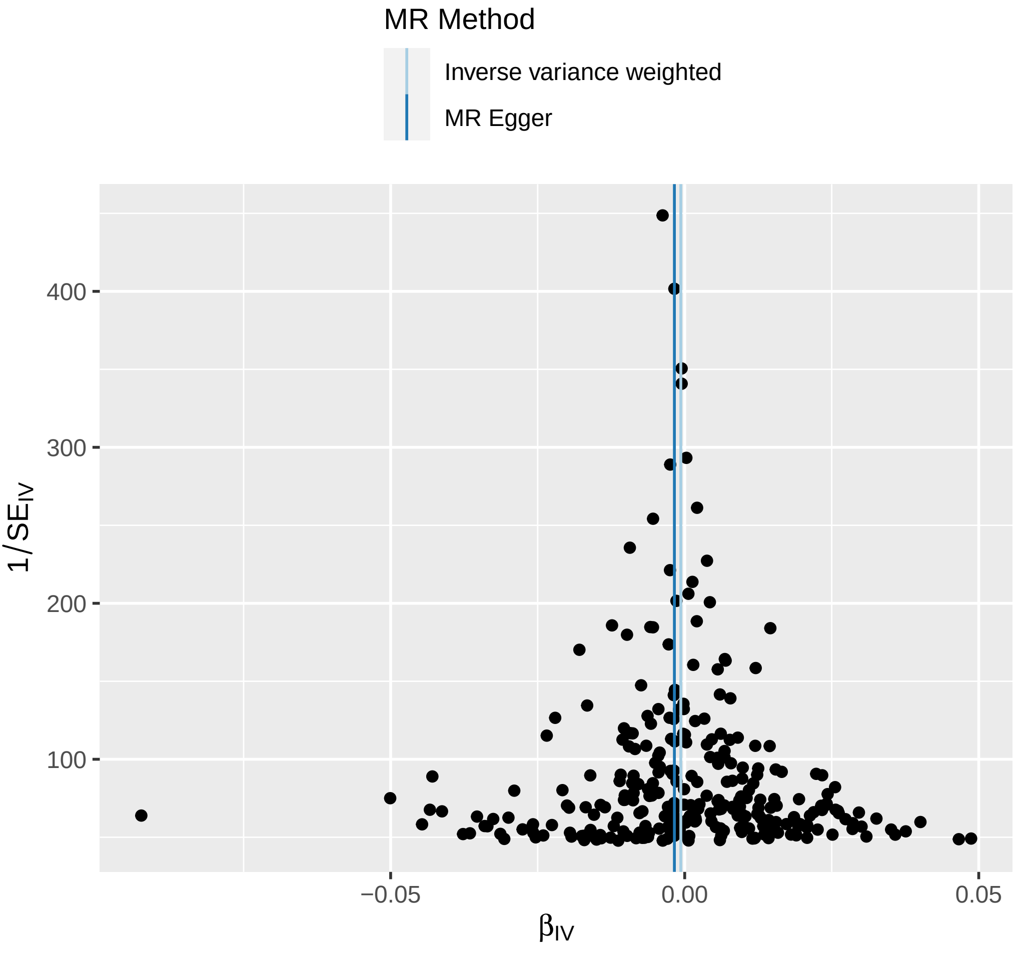

Supplementary figure 3: Funnel plot to assess heterogeneity of monocyte count. The blue line represents the inverse variance weighted estimate, and the dark blue line represents the Mendelian Randomization Egger estimate.

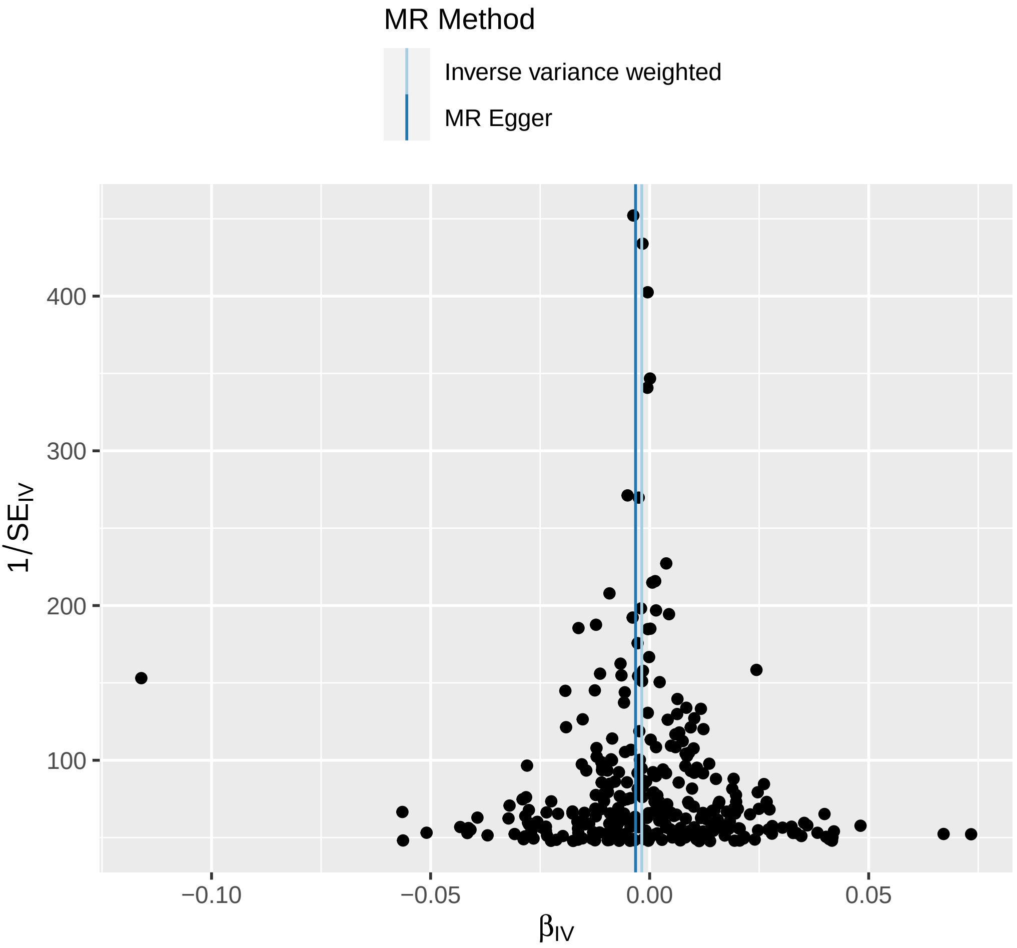

Supplementary figure 4: Funnel plot to assess heterogeneity of red blood cell distribution width. The blue line represents the inverse variance weighted estimate, and the dark blue line represents the Mendelian Randomization Egger estimate.

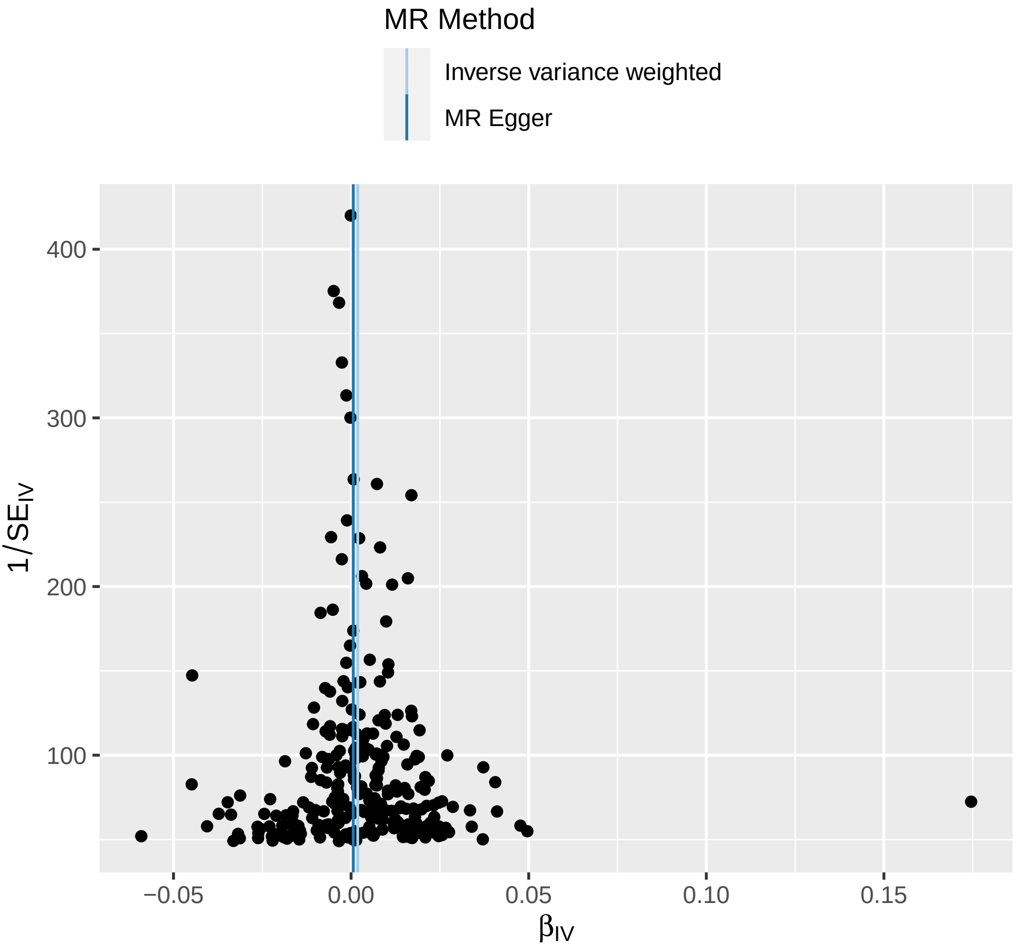

Supplementary figure 5: Funnel plot to assess heterogeneity of red blood cell count. The blue line represents the inverse variance weighted estimate, and the dark blue line represents the Mendelian Randomization Egger estimate.

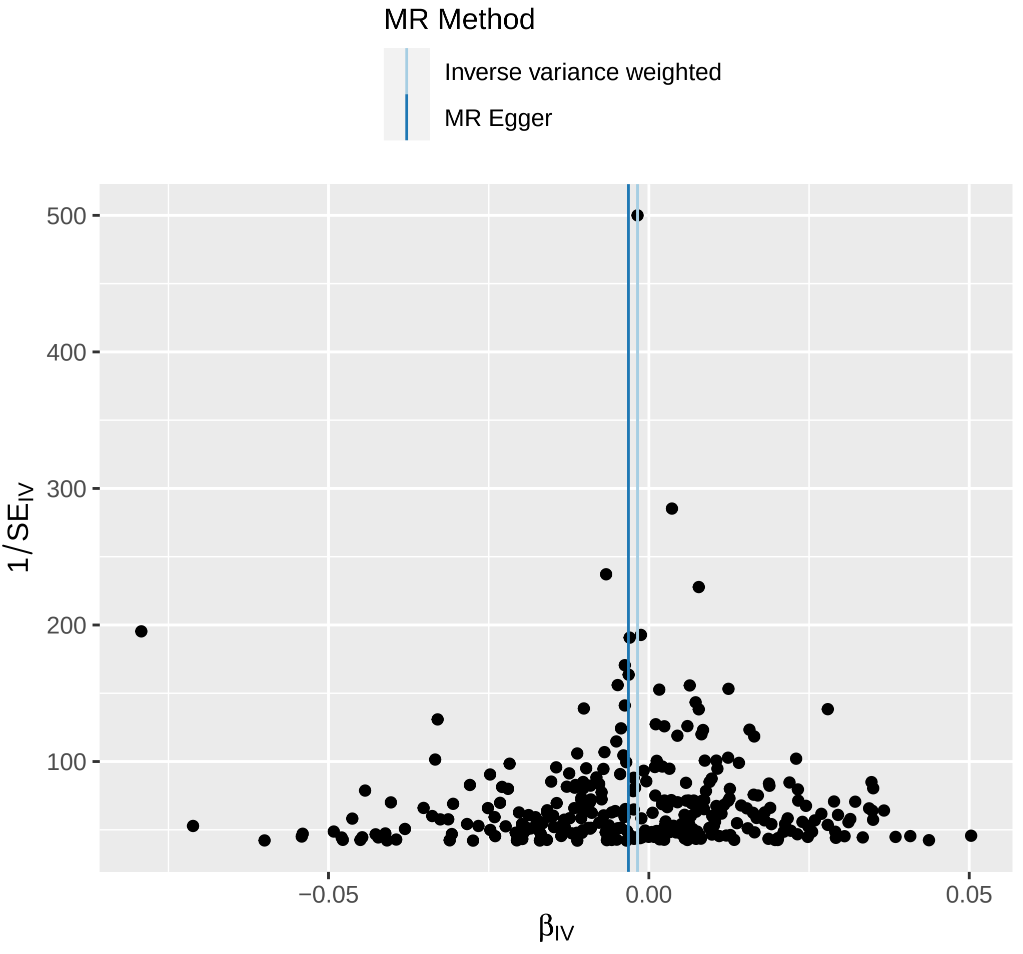

Supplementary figure 6: Funnel plot to assess heterogeneity of lymphocyte percentage. The blue line represents the inverse variance weighted estimate, and the dark blue line represents the Mendelian Randomization Egger estimate.

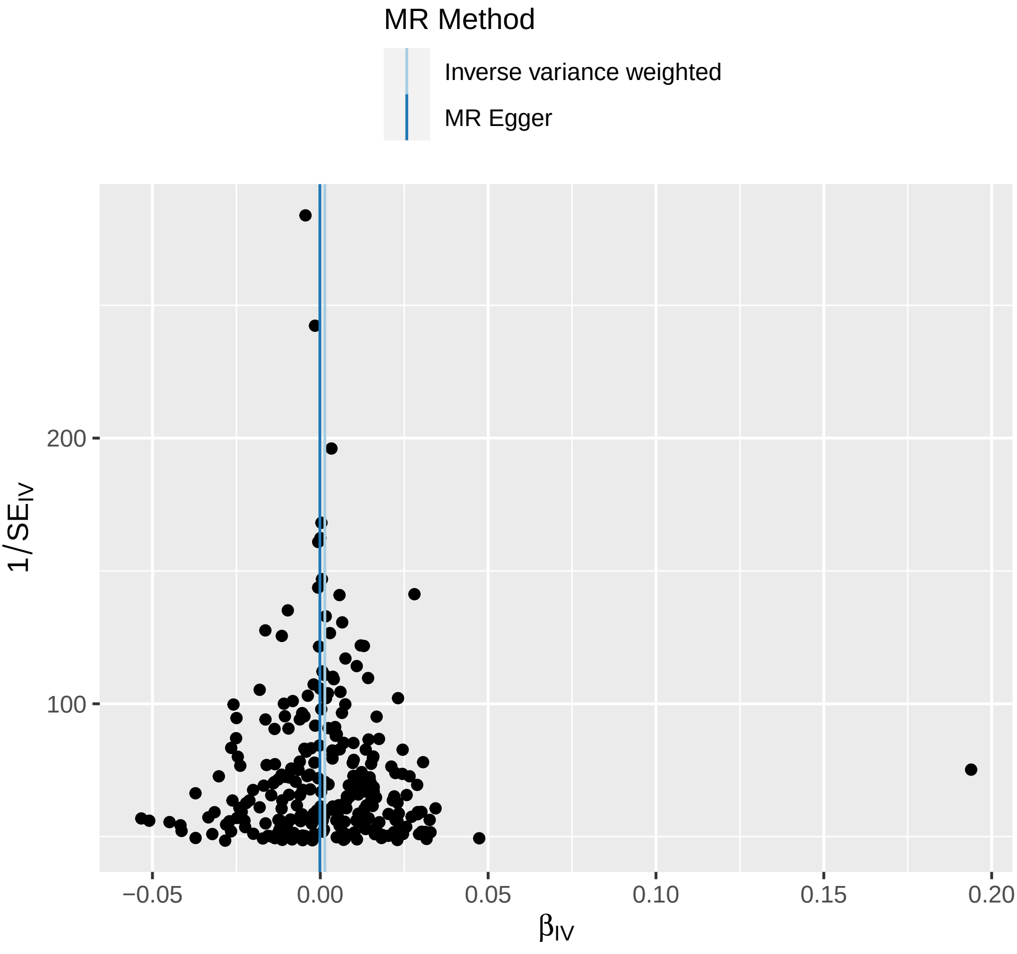

Supplementary figure 7: Funnel plot to assess heterogeneity of lymphocyte count. The blue line represents the inverse variance weighted estimate, and the dark blue line represents the Mendelian Randomization Egger estimate.

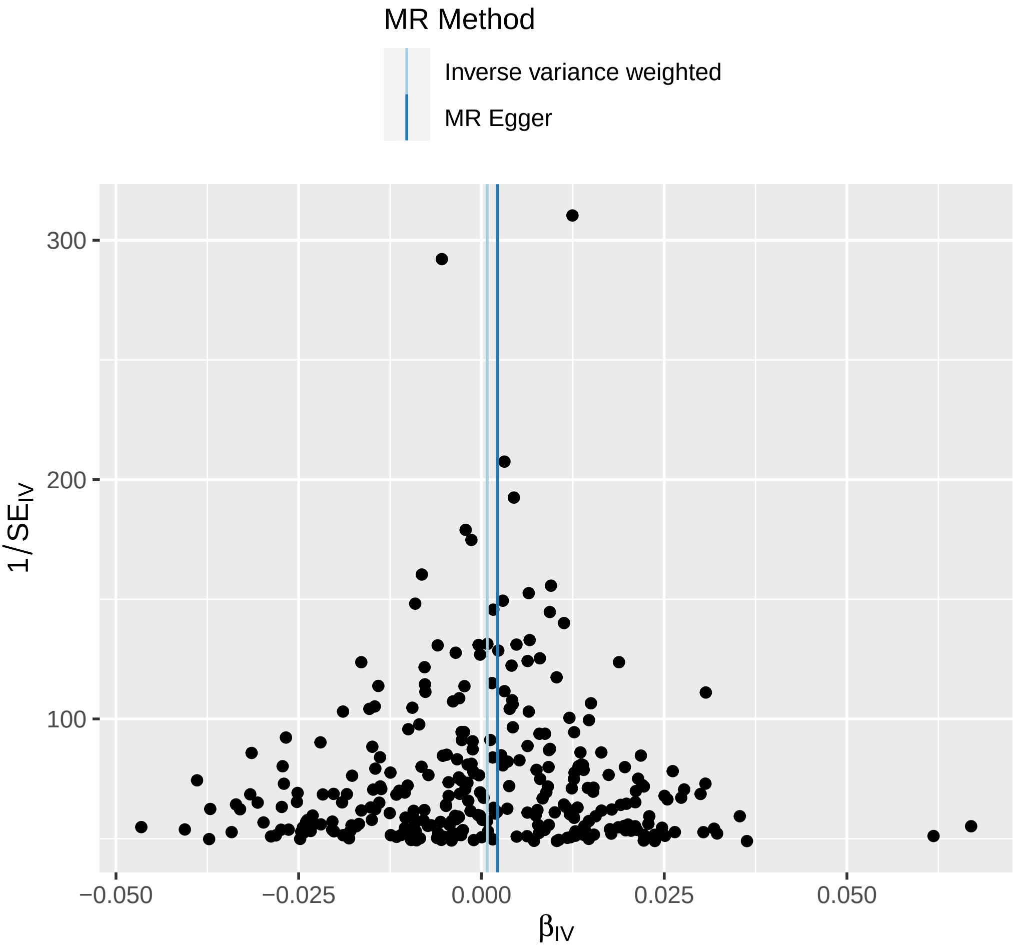

Supplementary figure 8: Funnel plot to assess heterogeneity of mean corpuscular volume. The blue line represents the inverse variance weighted estimate, and the dark blue line represents the Mendelian Randomization Egger estimate.

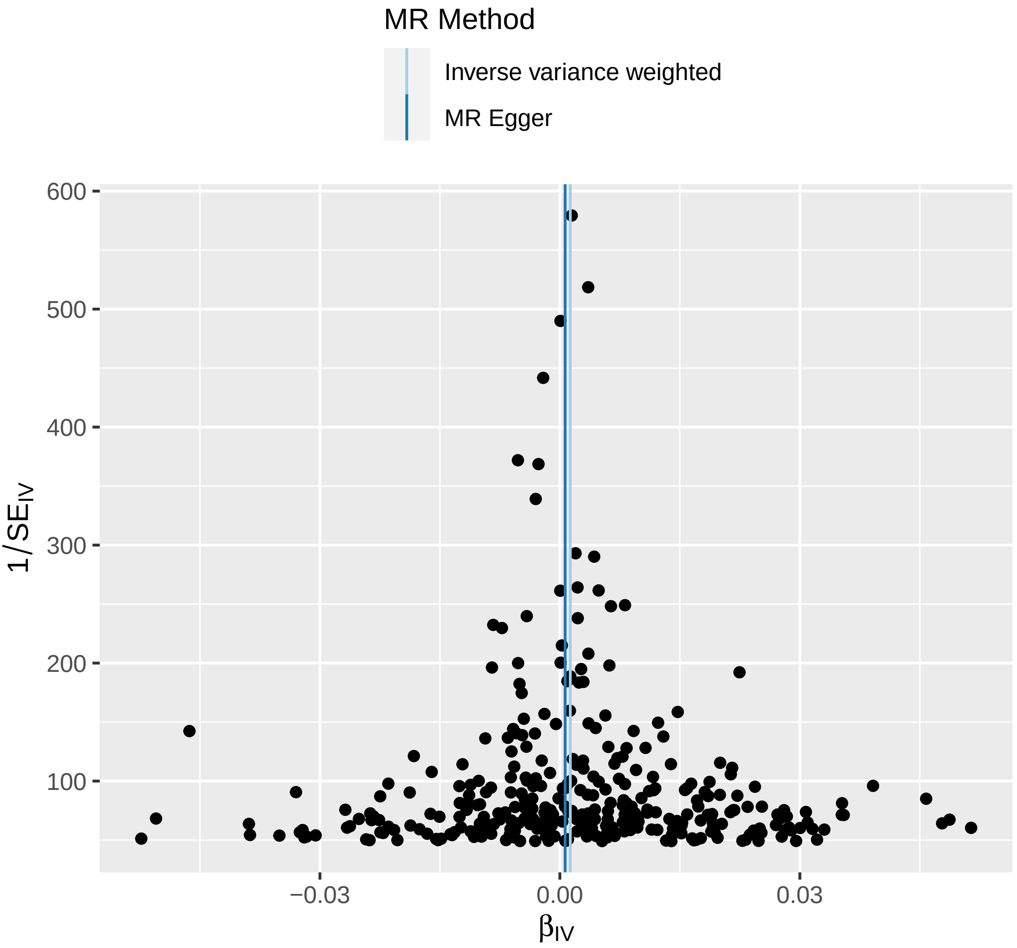

Supplementary figure 9: Funnel plot to assess heterogeneity of mean corpuscular haemoglobin concentration. The blue line represents the inverse variance weighted estimate, and the dark blue line represents the Mendelian Randomization Egger estimate.

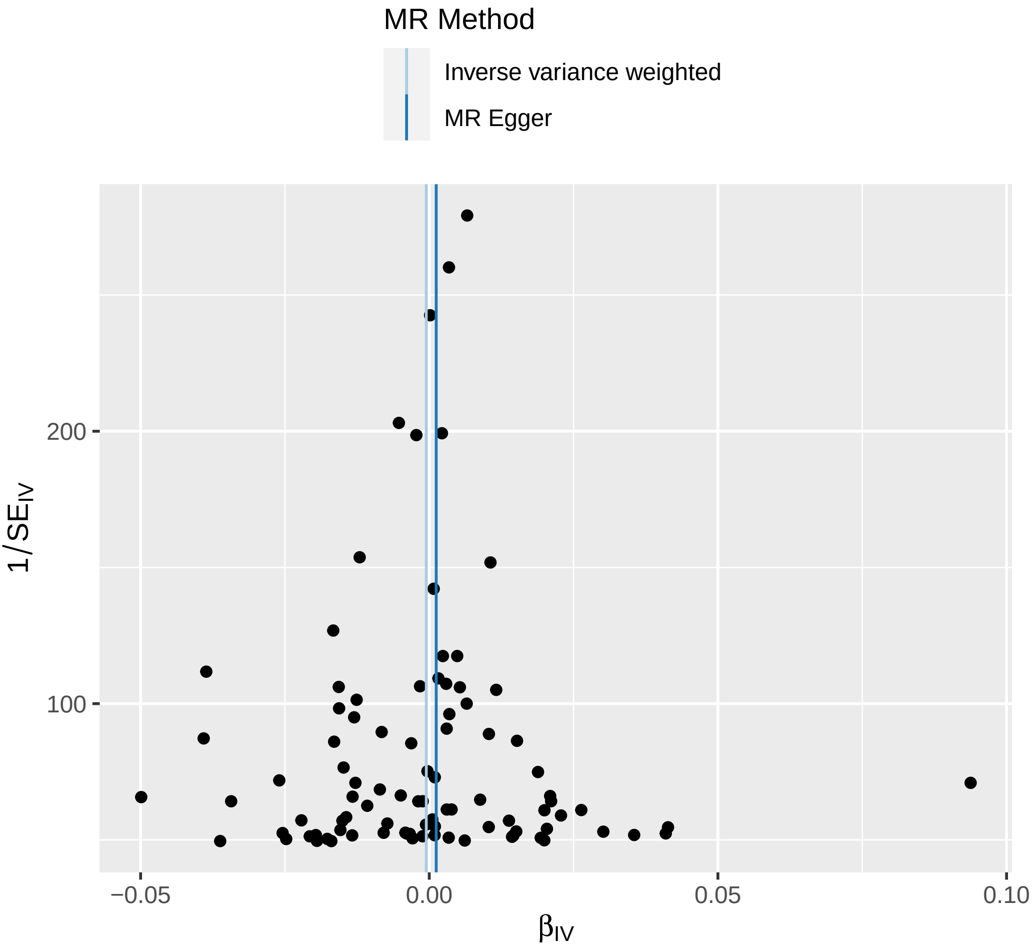

Supplementary figure 10: Funnel plot to assess heterogeneity of mean corpuscular volume of reticulocyte. The blue line represents the inverse variance weighted estimate, and the dark blue line represents the Mendelian Randomization Egger estimate.

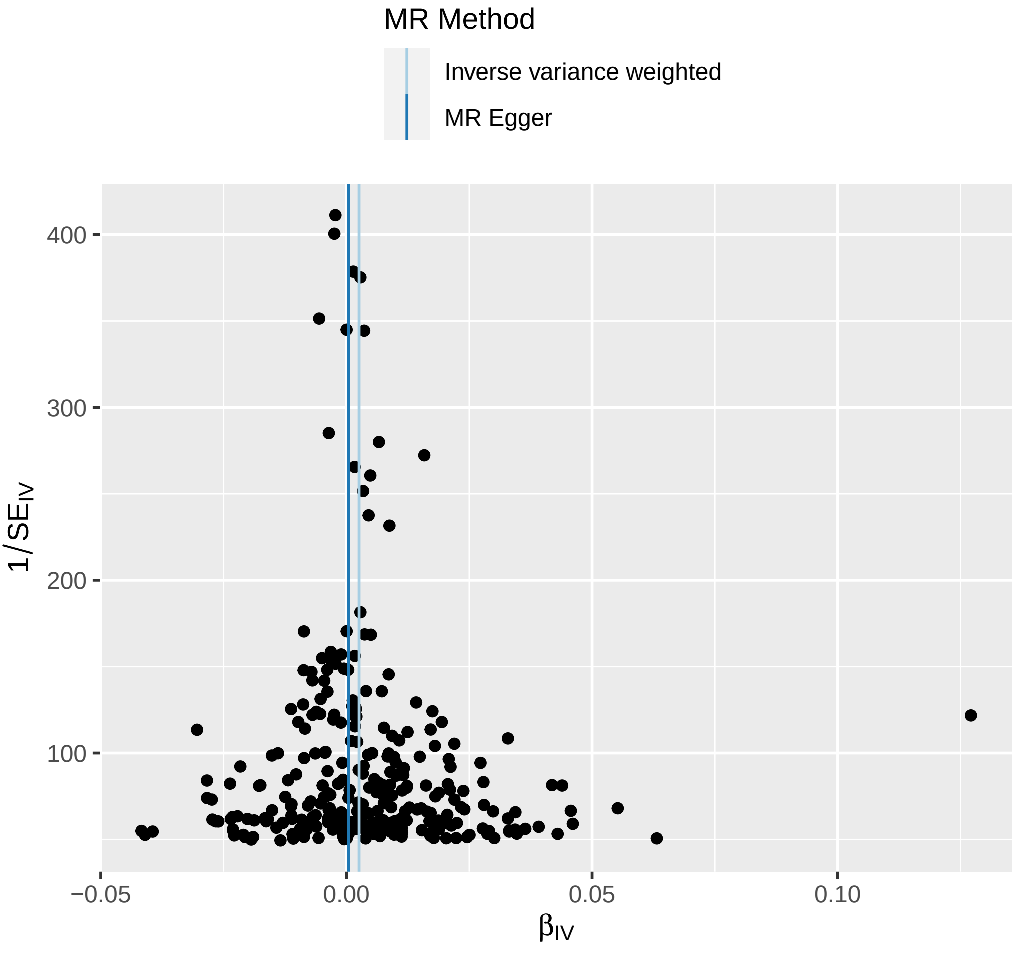

Supplementary figure 11: Funnel plot to assess heterogeneity of mean platelet volume. The blue line represents the inverse variance weighted estimate, and the dark blue line represents the Mendelian Randomization Egger estimate.

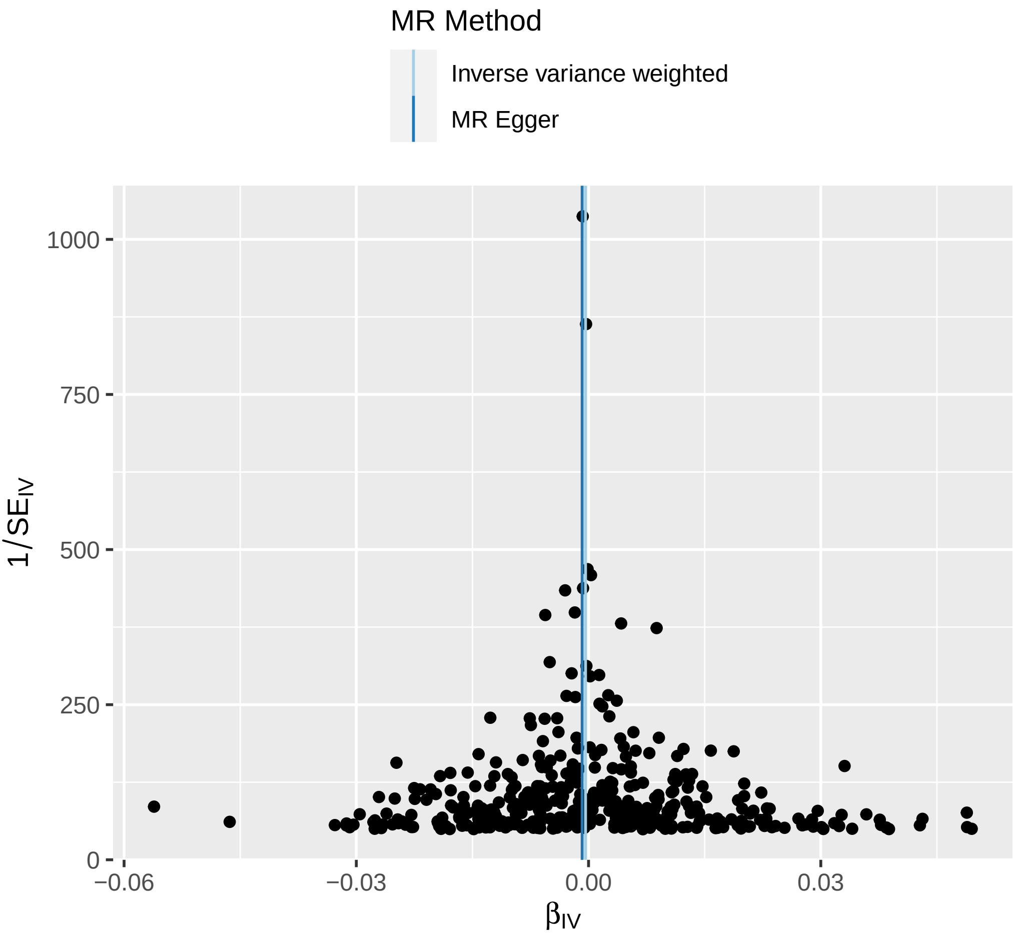

Supplementary figure 12: Funnel plot to assess heterogeneity of basophil percentage of granulocytes. The blue line represents the inverse variance weighted estimate, and the dark blue line represents the Mendelian Randomization Egger estimate.

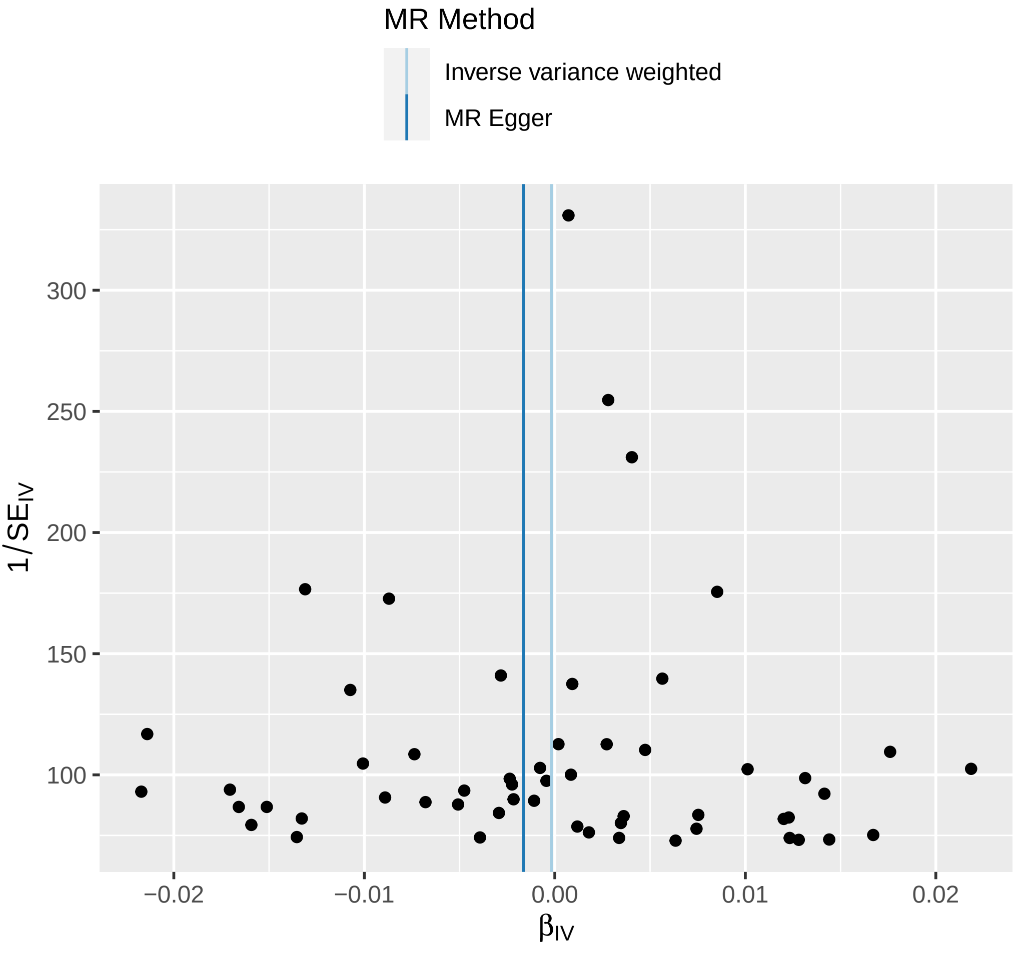

Supplementary figure 13: Funnel plot to assess heterogeneity of sum basophil neutrophil count. The blue line represents the inverse variance weighted estimate, and the dark blue line represents the Mendelian Randomization Egger estimate.

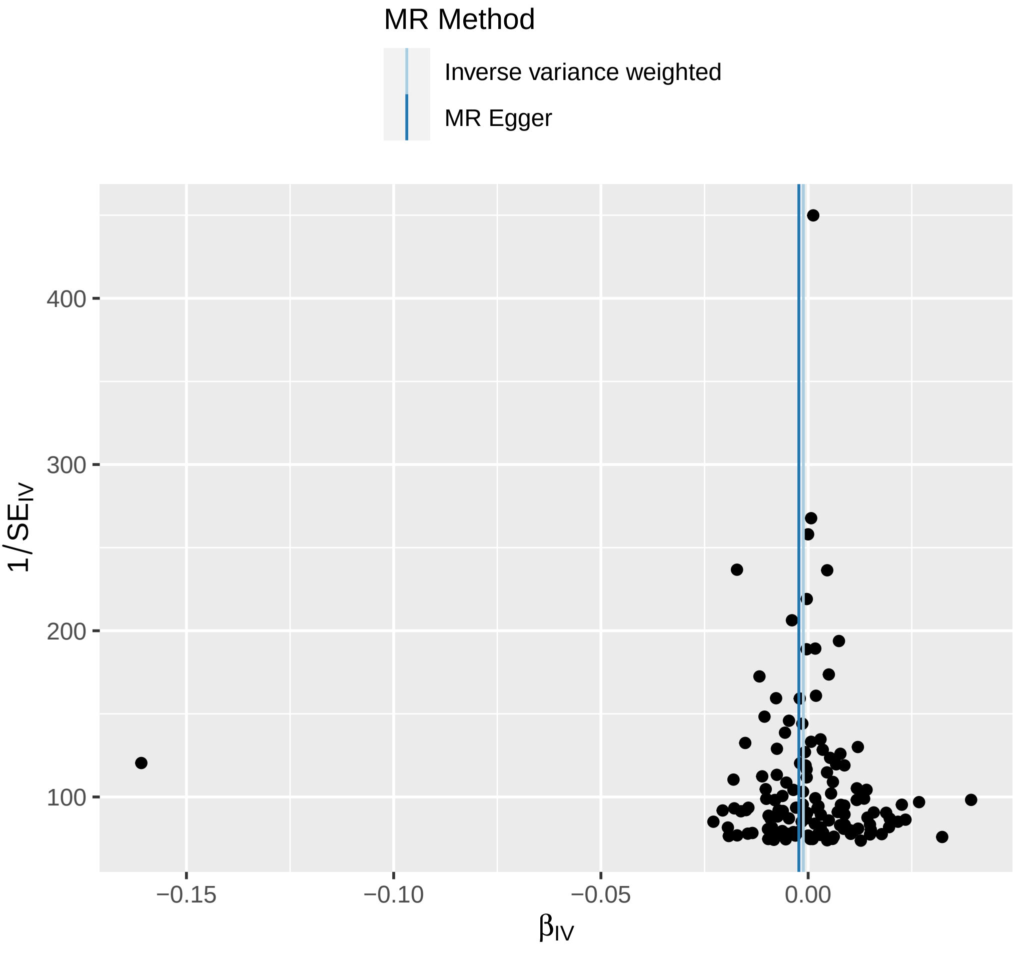

Supplementary figure 14: Funnel plot to assess heterogeneity of eosinophil percentage of granulocytes. The blue line represents the inverse variance weighted estimate, and the dark blue line represents the Mendelian Randomization Egger estimate.

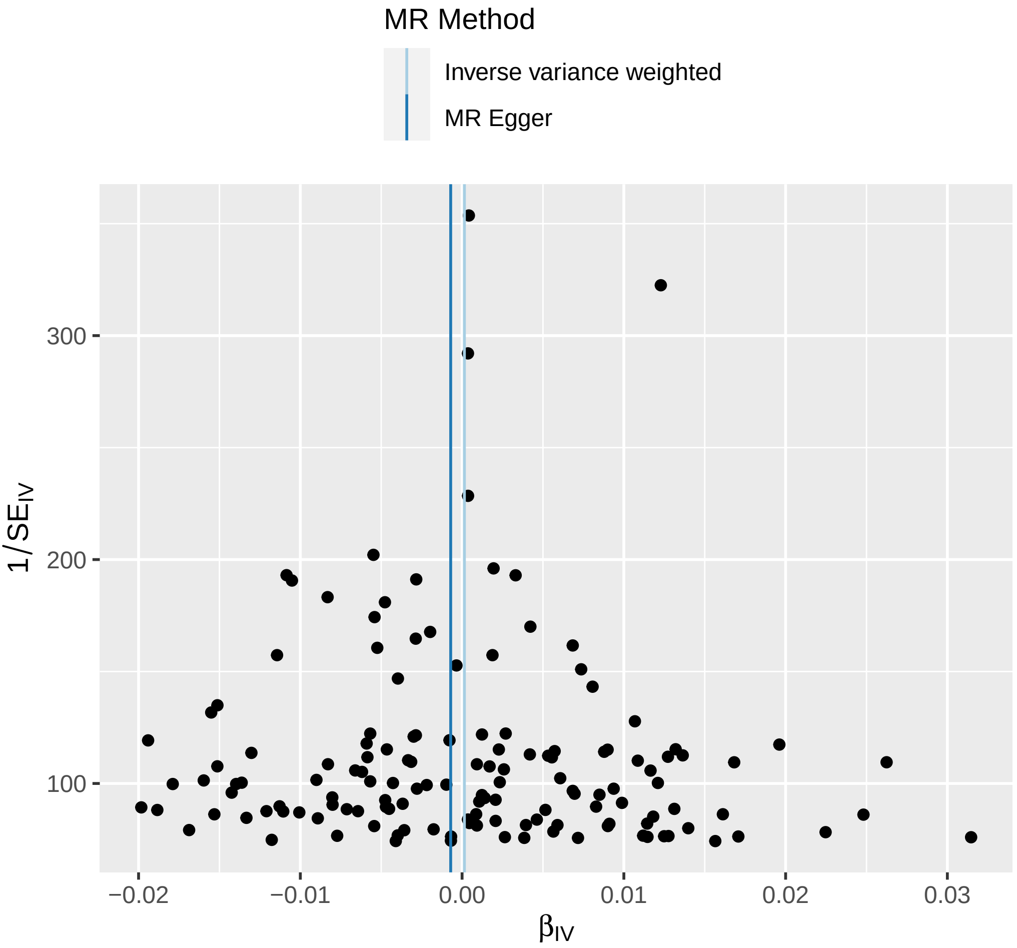

Supplementary figure 15: Funnel plot to assess heterogeneity of eosinophil counts. The blue line represents the inverse variance weighted estimate, and the dark blue line represents the Mendelian Randomization Egger estimate.

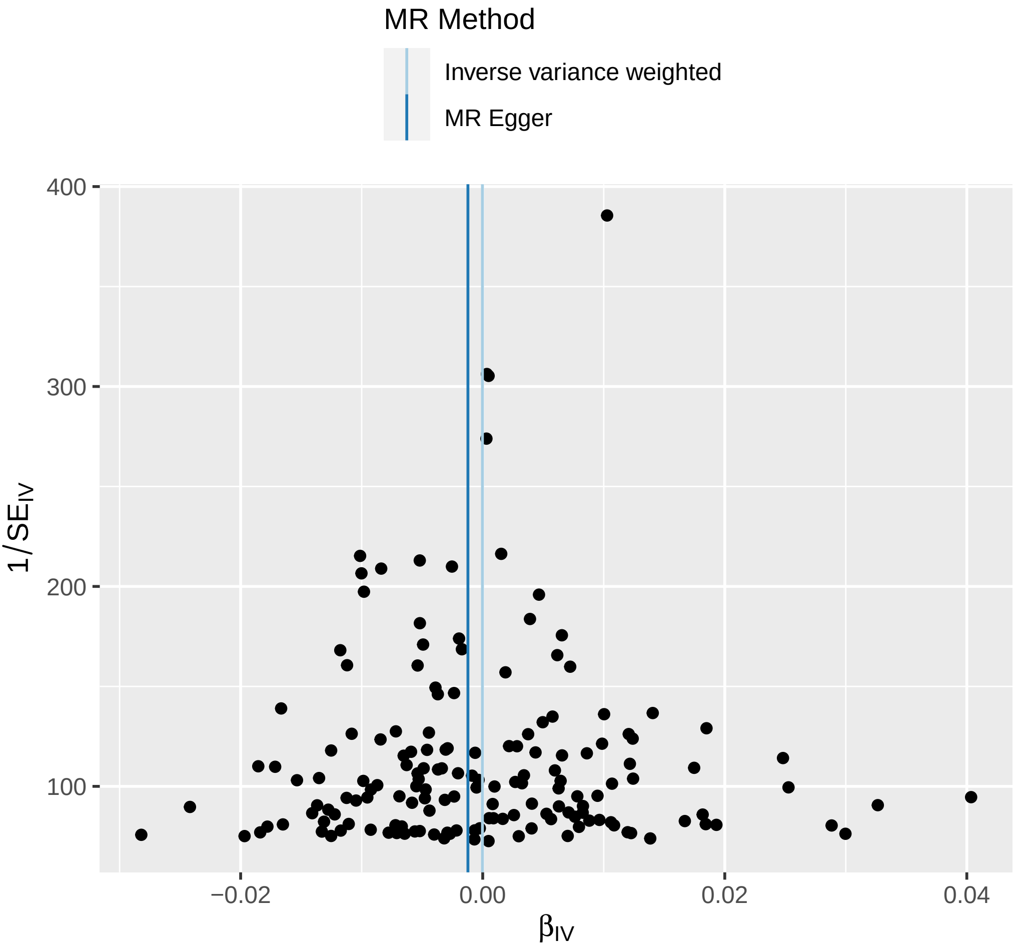

Supplementary figure 16: Funnel plot to assess heterogeneity of reticulocyte percentage. The blue line represents the inverse variance weighted estimate, and the dark blue line represents the Mendelian Randomization Egger estimate.

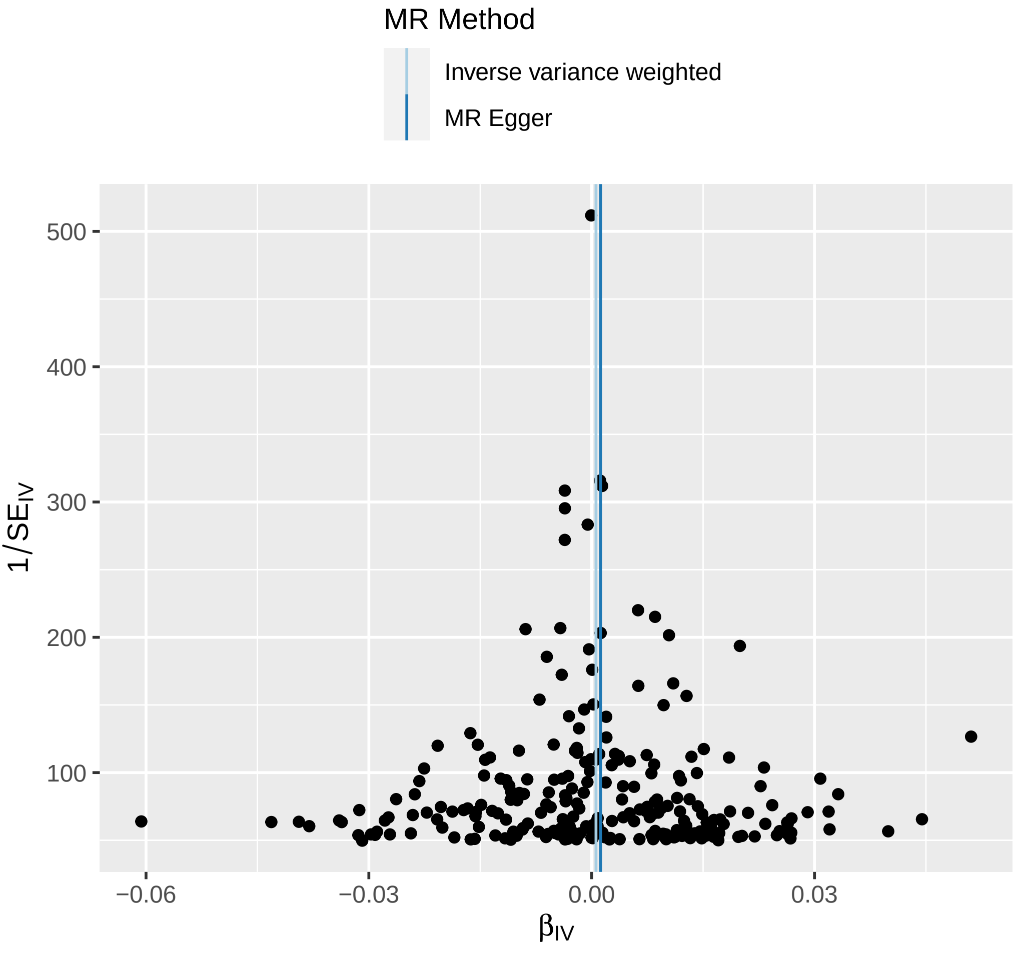

Supplementary figure 17: Funnel plot to assess heterogeneity of reticulocyte count. The blue line represents the inverse variance weighted estimate, and the dark blue line represents the Mendelian Randomization Egger estimate.

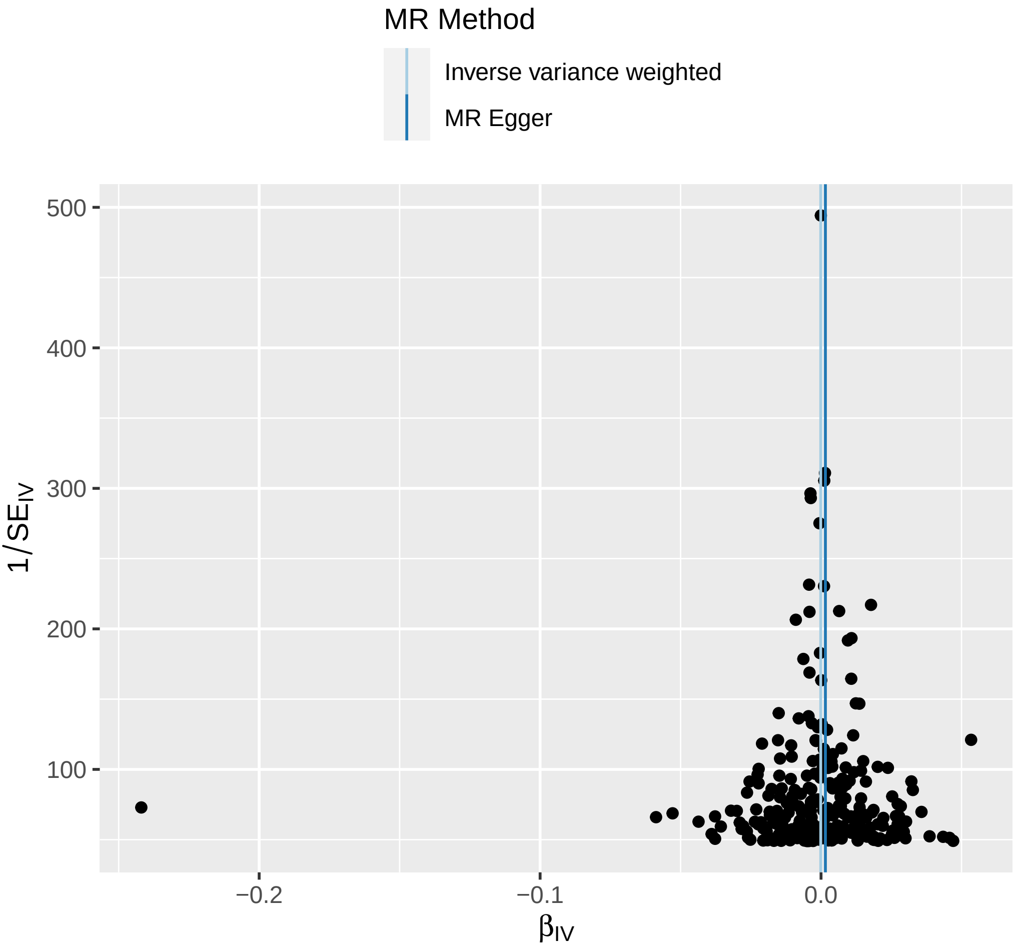

Supplementary figure 18: Funnel plot to assess heterogeneity of haemoglobin concentration. The blue line represents the inverse variance weighted estimate, and the dark blue line represents the Mendelian Randomization Egger estimate.

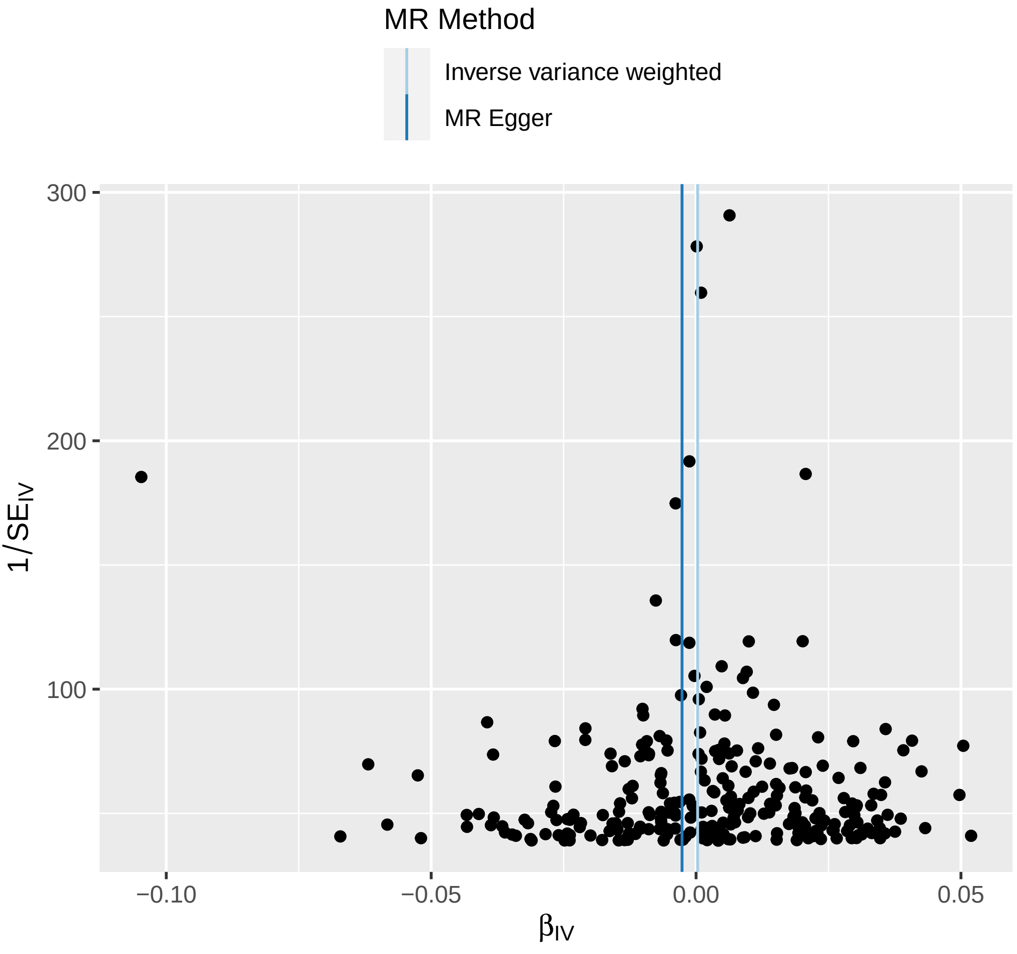

Supplementary figure 19: Funnel plot to assess heterogeneity of hematocrit. The blue line represents the inverse variance weighted estimate, and the dark blue line represents the Mendelian Randomization Egger estimate.

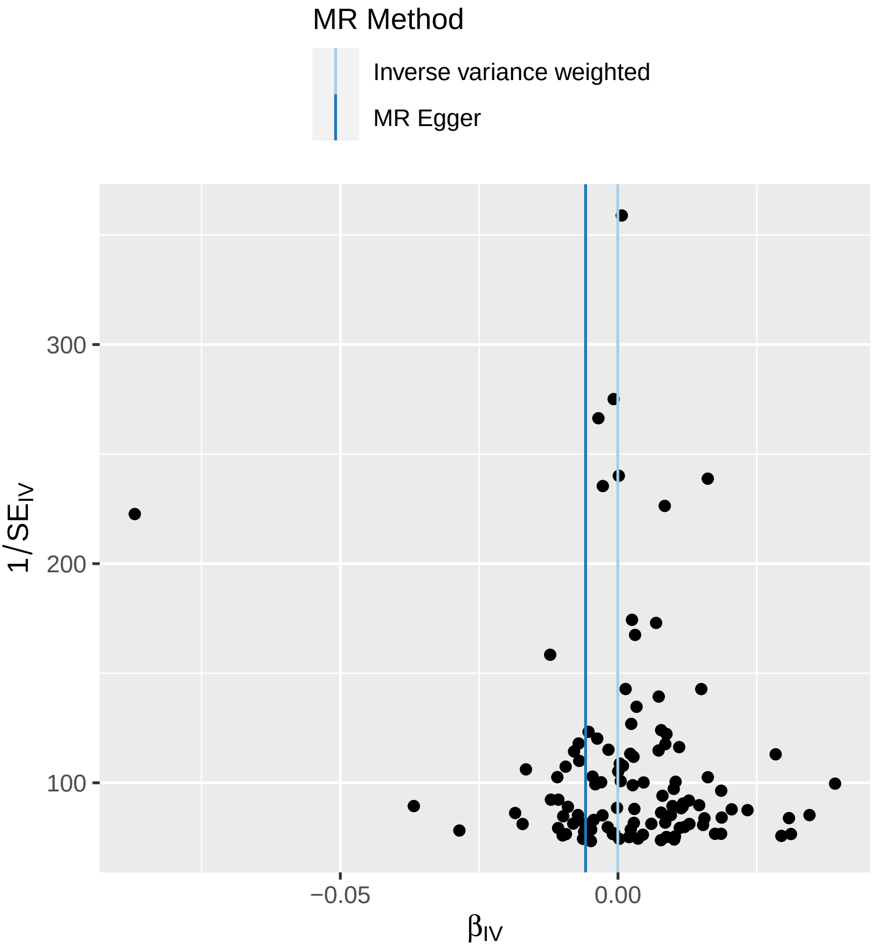

Supplementary figure 20: Funnel plot to assess heterogeneity of platelet distribution width. The blue line represents the inverse variance weighted estimate, and the dark blue line represents the Mendelian Randomization Egger estimate.

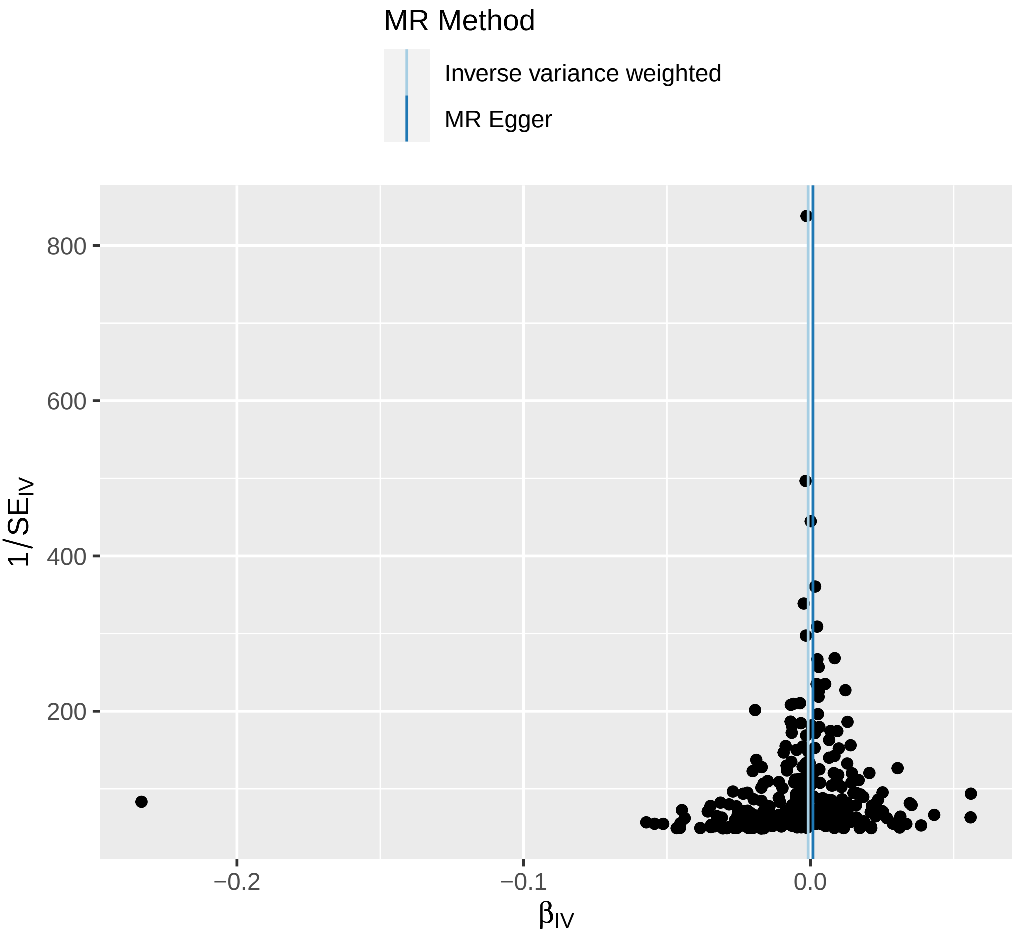

Supplementary figure 21: Funnel plot to assess heterogeneity of patelet count. The blue line represents the inverse variance weighted estimate, and the dark blue line represents the Mendelian Randomization Egger estimate.

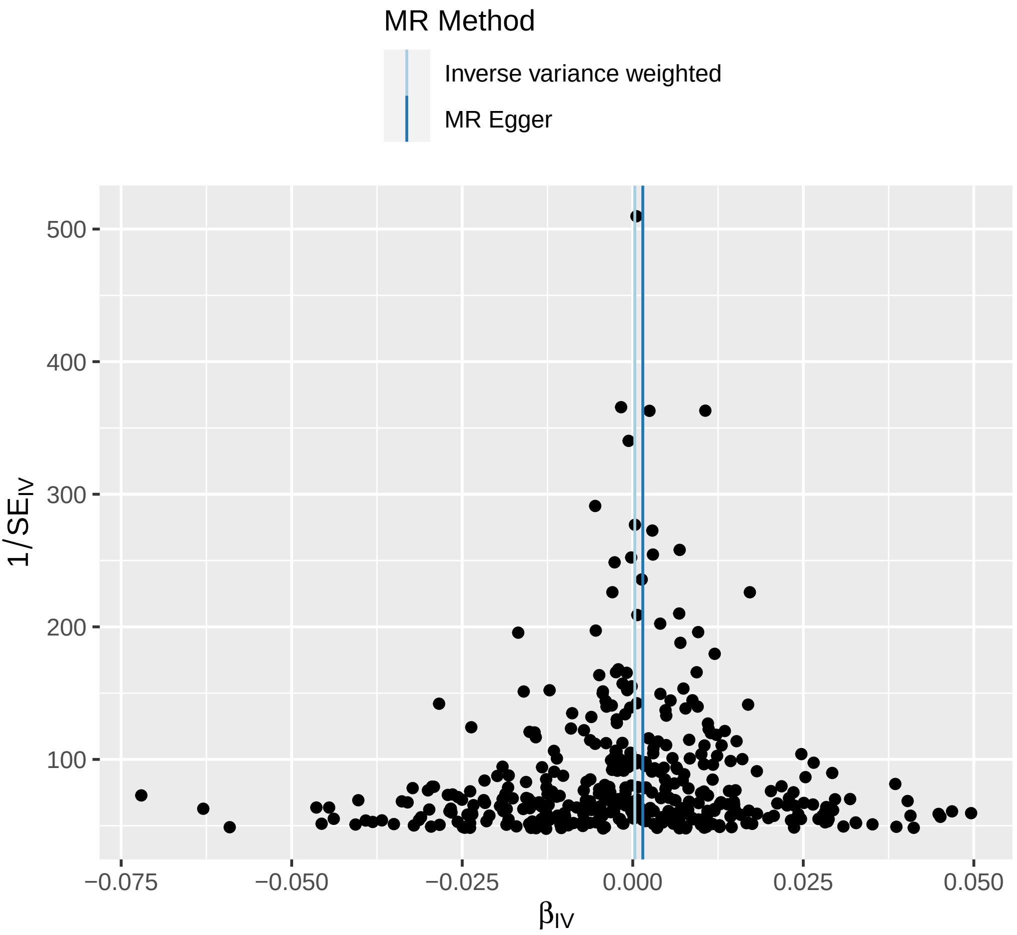

Supplementary figure 22: Funnel plot to assess heterogeneity of neutrophil percentage of granulocytes. The blue line represents the inverse variance weighted estimate, and the dark blue line represents the Mendelian Randomization Egger estimate.

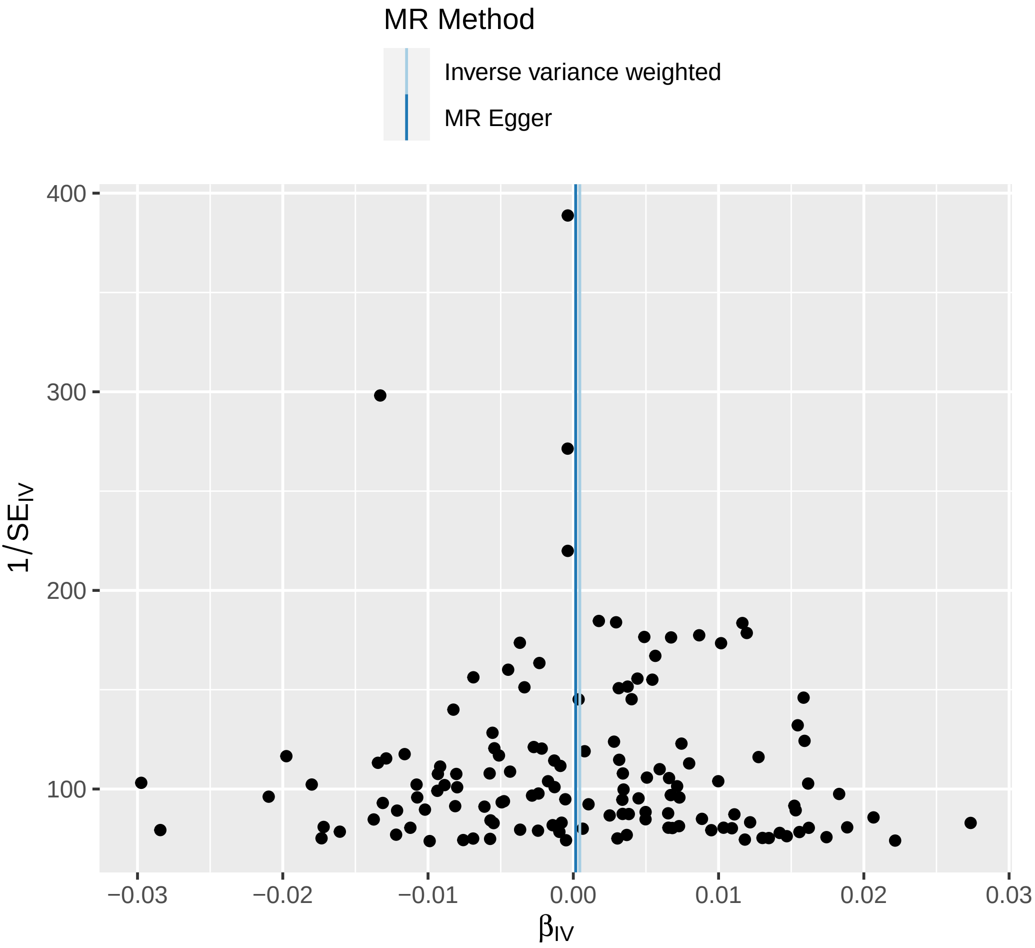

Supplementary figure 23: Funnel plot to assess heterogeneity of neutrophil count. The blue line represents the inverse variance weighted estimate, and the dark blue line represents the Mendelian Randomization Egger estimate.

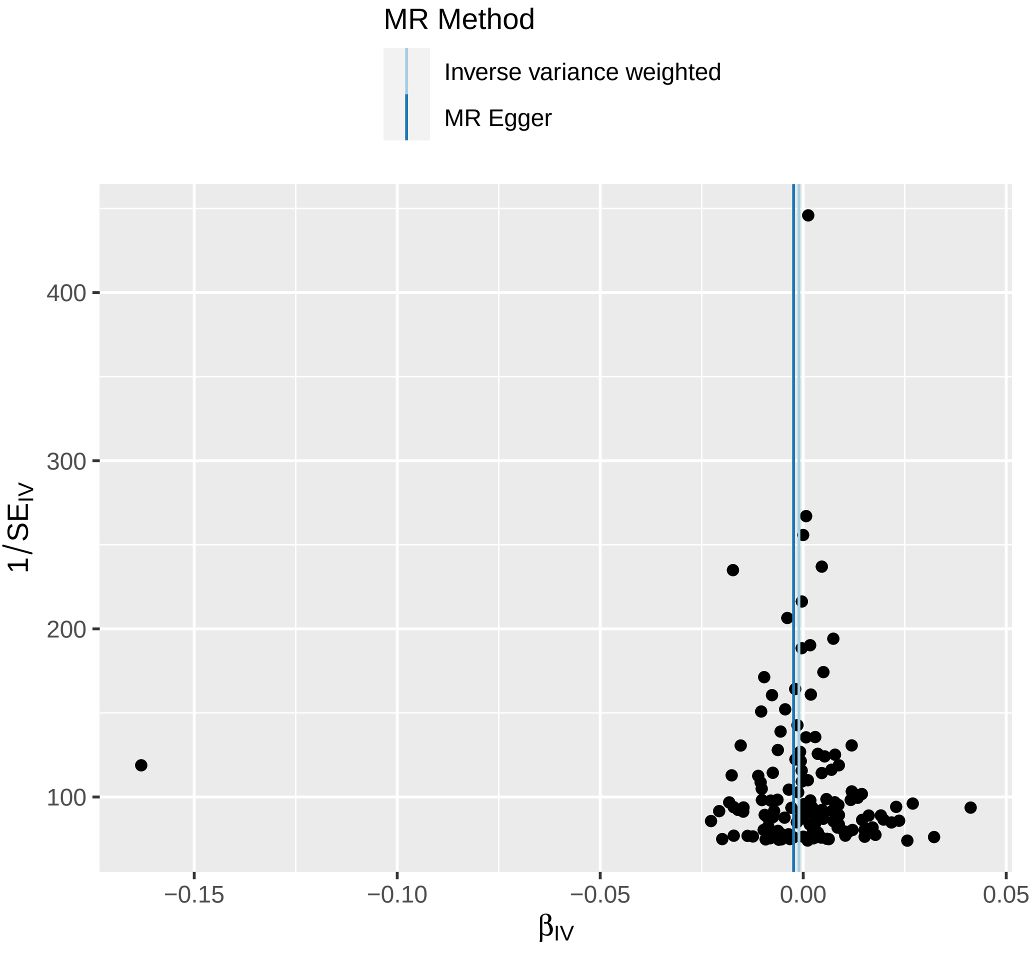

Supplementary Figure 24: Forest plot of MR leave-one-out sensitivity analysis of the causal effects of single nucleotide polymorphisms associated with white blood cell count on venous thromboembolism.

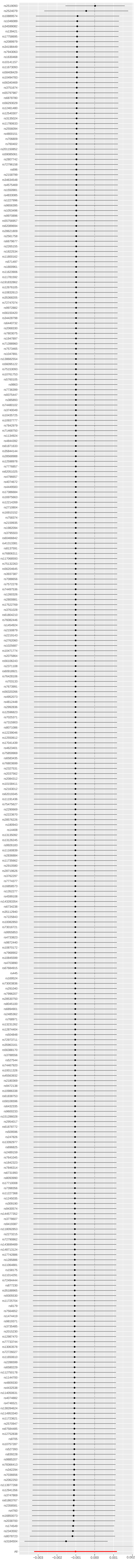

Supplementary Figure 25: Forest plot of MR leave-one-out sensitivity analysis of the causal effects of single nucleotide polymorphisms associated with monocyte percentage on venous thromboembolism.

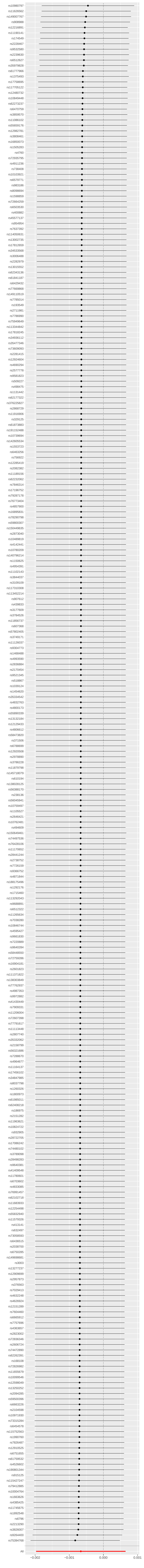

Supplementary Figure 26: Forest plot of MR leave-one-out sensitivity analysis of the causal effects of single nucleotide polymorphisms associated with monocyte count on venous thromboembolism.

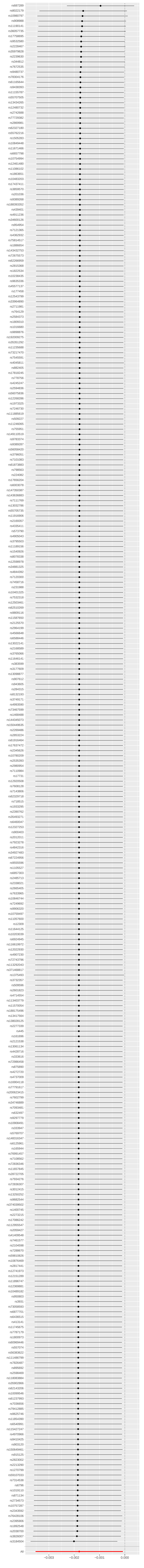

Supplementary Figure 27: Forest plot of MR leave-one-out sensitivity analysis of the causal effects of single nucleotide polymorphisms associated with red blood cell distribution width on venous thromboembolism.

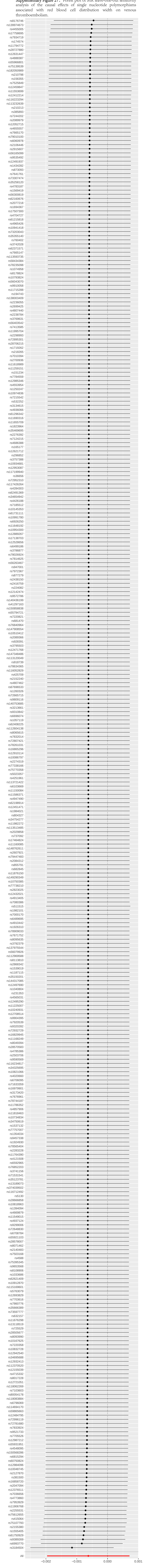

Supplementary Figure 28: Forest plot of MR leave-one-out sensitivity analysis of the causal effects of single nucleotide polymorphisms associated with red blood cell count on venous thromboembolism.

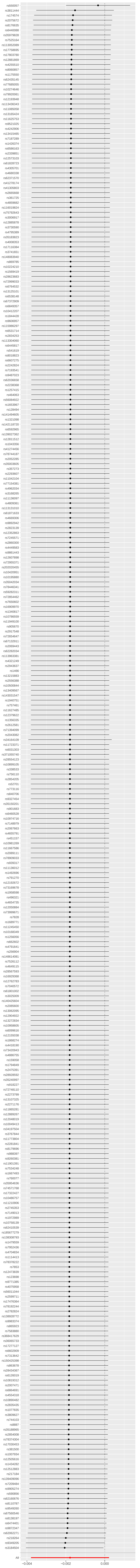

Supplementary Figure 29: Forest plot of MR leave-one-out sensitivity analysis of the causal effects of single nucleotide polymorphisms associated with lymphocyte percentage on venous thromboembolism.

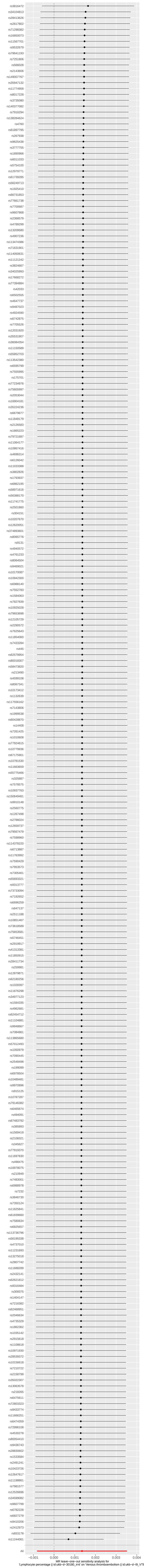

Supplementary Figure 30: Forest plot of MR leave-one-out sensitivity analysis of the causal effects of single nucleotide polymorphisms associated with lymphocyte count on venous thromboembolism.

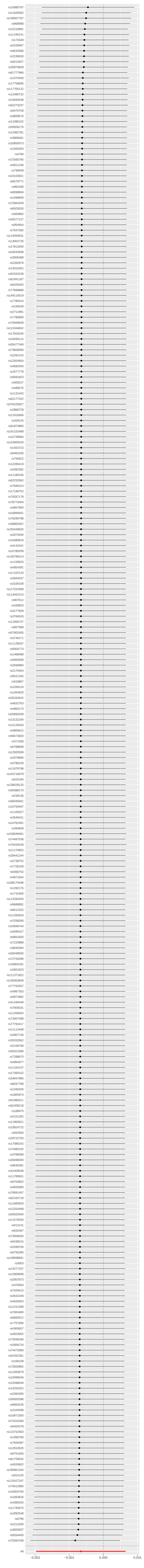

Supplementary Figure 31: Forest plot of MR leave-one-out sensitivity analysis of the causal effects of single nucleotide polymorphisms associated with mean corpuscular volume on venous thromboembolism.

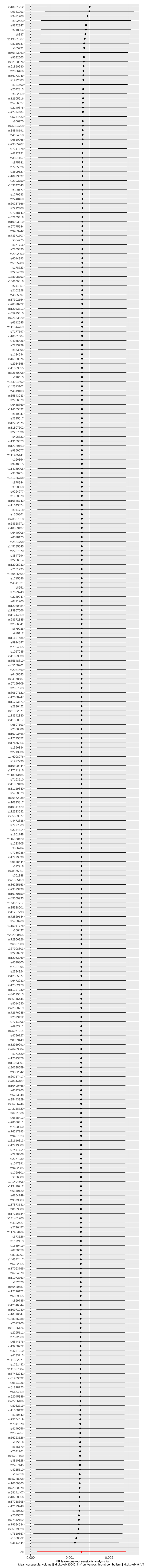

Supplementary Figure 32: Forest plot of MR leave-one-out sensitivity analysis of the causal effects of single nucleotide polymorphisms associated with mean corpuscular haemoglobin concentration on venous thromboembolism.

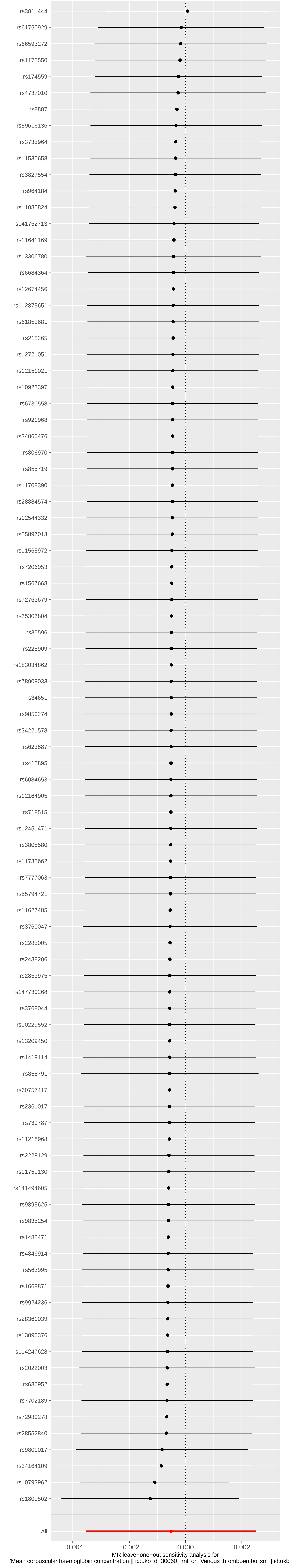

Supplementary Figure 33: Forest plot of MR leave-one-out sensitivity analysis of the causal effects of single nucleotide polymorphisms associated with mean corpuscular volume of reticulocyte on venous thromboembolism

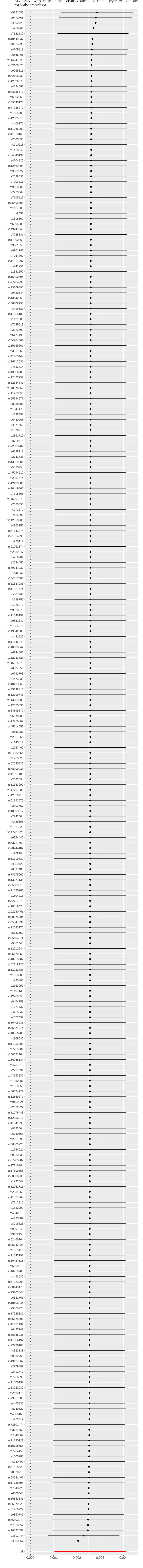

Supplementary Figure 34: Forest plot of MR leave-one-out sensitivity analysis of the causal effects of single nucleotide polymorphisms associated with mean platelet volume on venous thromboembolism

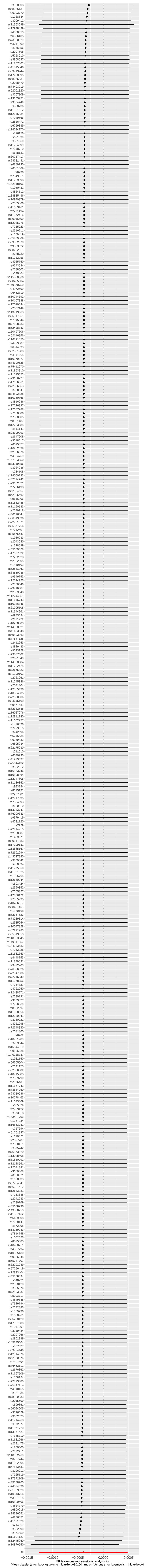

Supplementary Figure 35: Forest plot of MR leave-one-out sensitivity analysis of the causal effects of single nucleotide polymorphisms associated with basophil percentage of granulocytes on venous thromboembolism

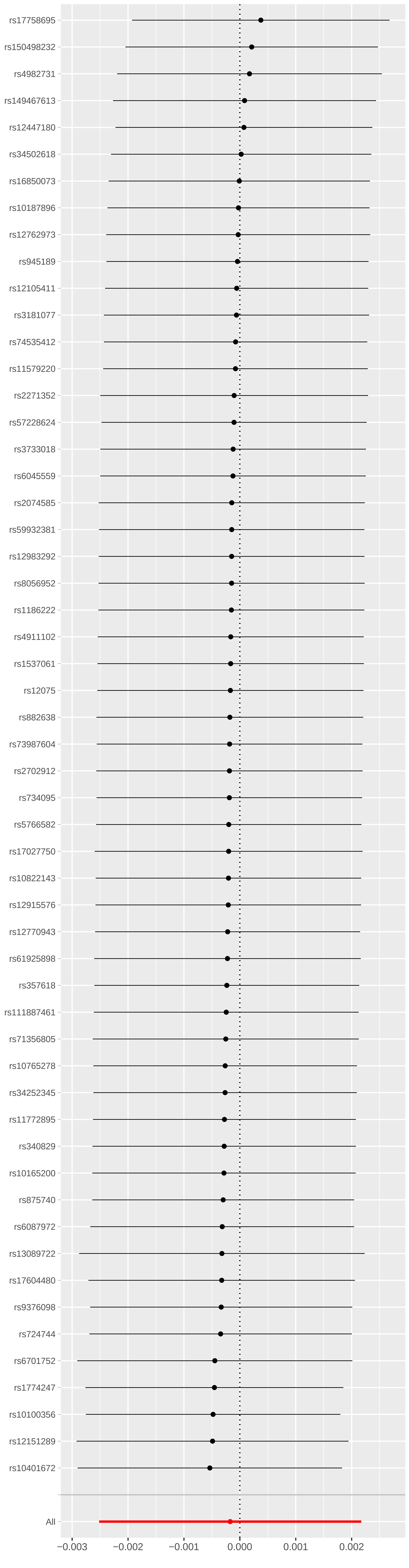

Supplementary Figure 36: Forest plot of MR leave-one-out sensitivity analysis of the causal effects of single nucleotide polymorphisms associated with sum basophil neutrophil counts on venous thromboembolism

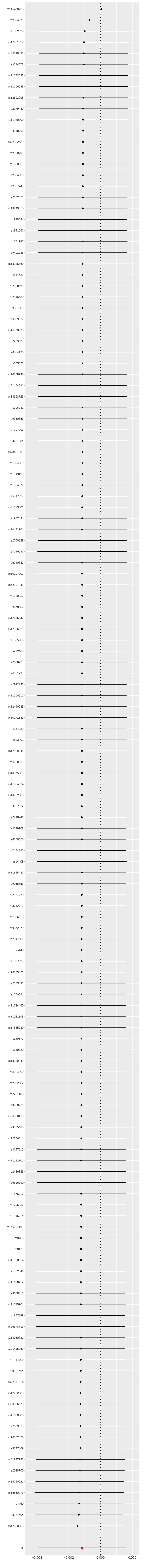

Supplementary Figure 37: Forest plot of MR leave-one-out sensitivity analysis of the causal effects of single nucleotide polymorphisms associated with eosinophil percentage of granulocytes on venous thromboembolism

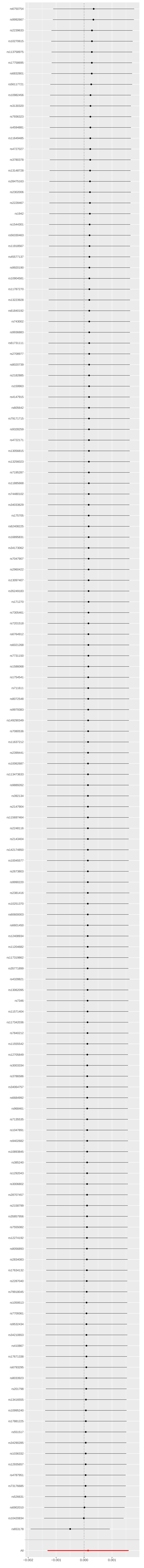

Supplementary Figure 38: Forest plot of MR leave-one-out sensitivity analysis of the causal effects of single nucleotide polymorphisms associated with eosinophil counts on venous thromboembolism.

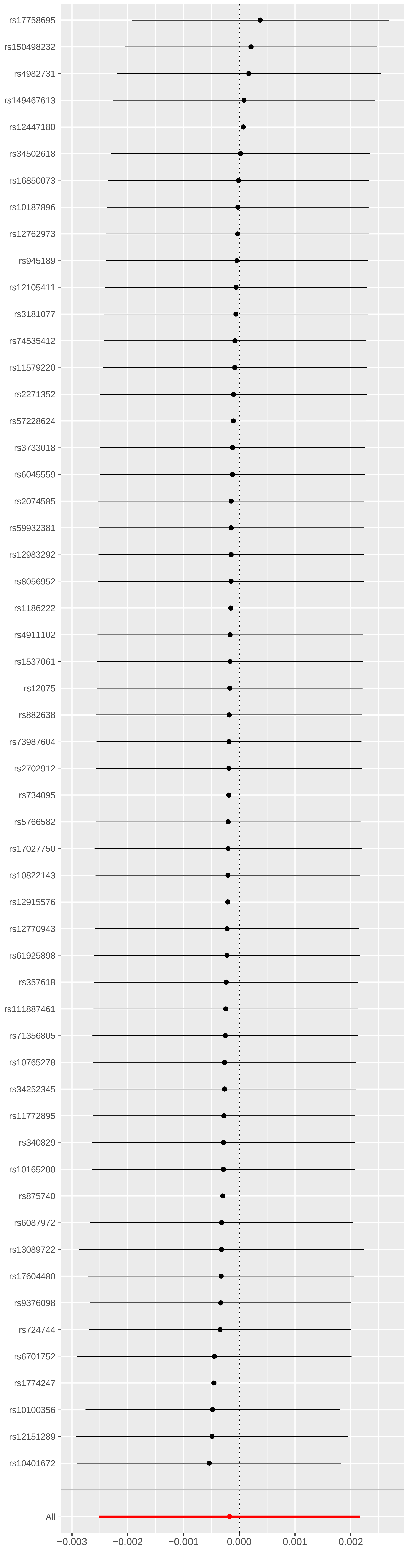

Supplementary Figure 39: Forest plot of MR leave-one-out sensitivity analysis of the causal effects of single nucleotide polymorphisms associated with reticulocyte percentage on venous thromboembolism

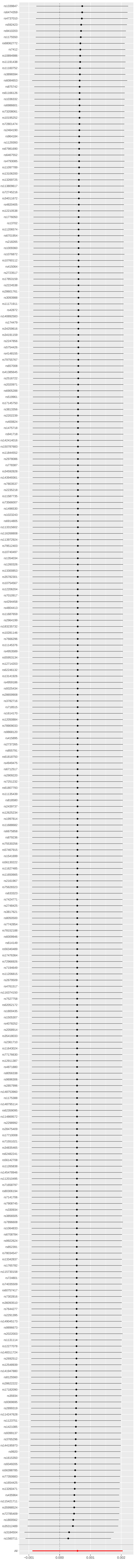

Supplementary Figure 40: Forest plot of MR leave-one-out sensitivity analysis of the causal effects of single nucleotide polymorphisms associated with reticulocyte count on venous thromboembolism

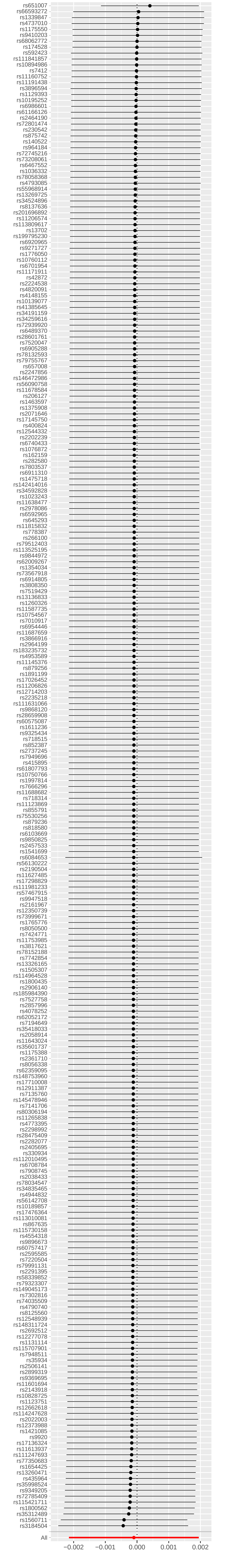

Supplementary Figure 41: Forest plot of MR leave-one-out sensitivity analysis of the causal effects of single nucleotide polymorphisms associated with haemoglobin concentration on venous thromboembolism.

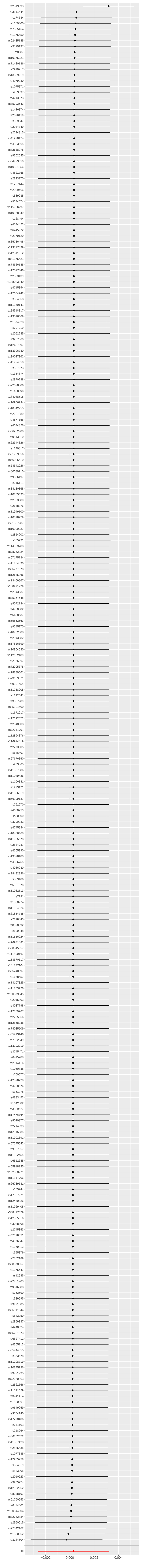

Supplementary Figure 42: Forest plot of MR leave-one-out sensitivity analysis of the causal effects of single nucleotide polymorphisms associated with hematocrit on venous thromboembolism.

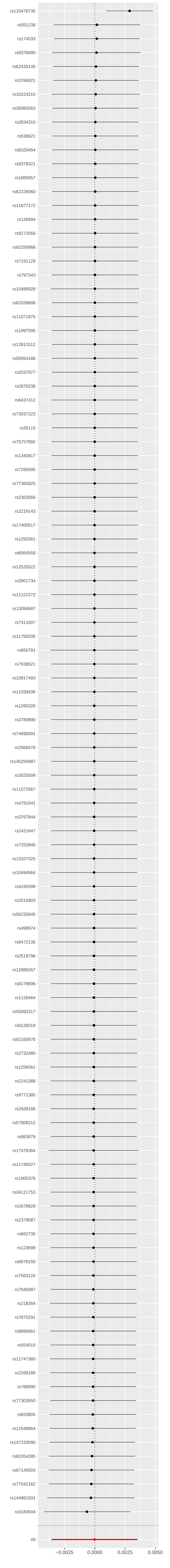

Supplementary Figure 43: Forest plot of MR leave-one-out sensitivity analysis of the causal effects of single nucleotide polymorphisms associated with Platelet distribution width on venous thromboembolism.

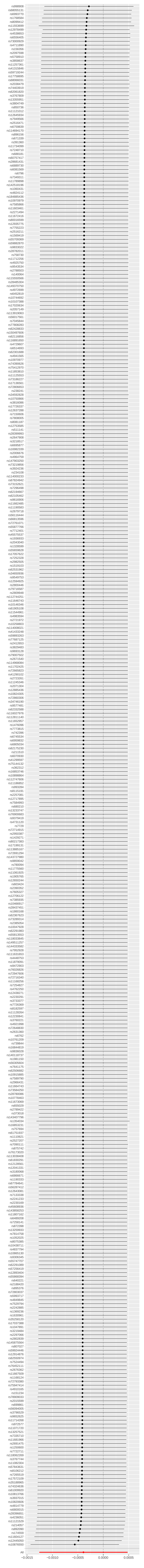

Supplementary Figure 44: Forest plot of MR leave-one-out sensitivity analysis of the causal effects of single nucleotide polymorphisms associated with platelet count on venous thromboembolism.

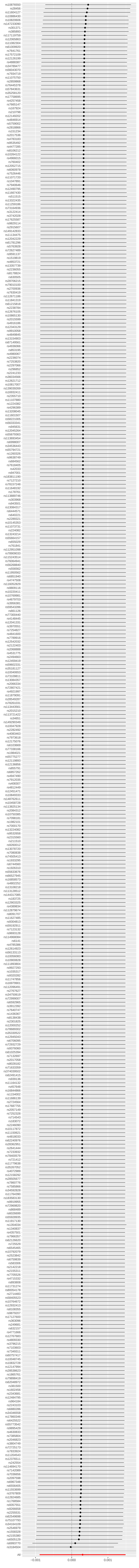

Supplementary Figure 45: Forest plot of MR leave-one-out sensitivity analysis of the causal effects of single nucleotide polymorphisms associated with Neutrophil percentage of granulocytes on venous thromboembolism.

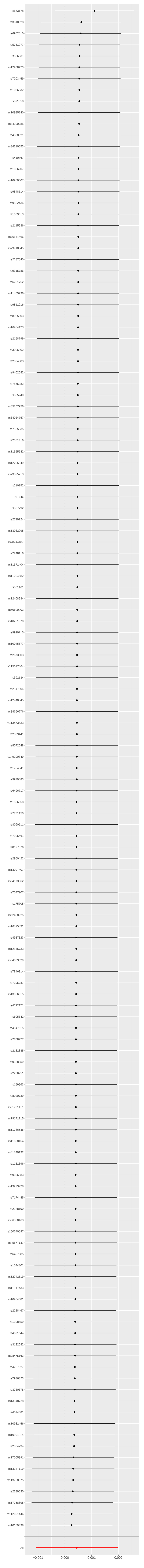

Supplementary Figure 46: Forest plot of MR leave-one-out sensitivity analysis of the causal effects of single nucleotide polymorphisms associated with neutrophil count on venous thromboembolism.

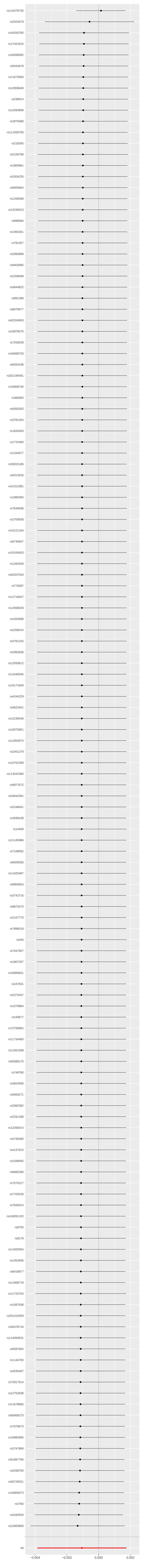

Supplementary Figure 47: Forest plot of the causal effects of single nucleotide polymorphisms associated with white blood cell count on venous thromboembolism.The significance of red lines are MR results of MR-Egger test and IVW method.

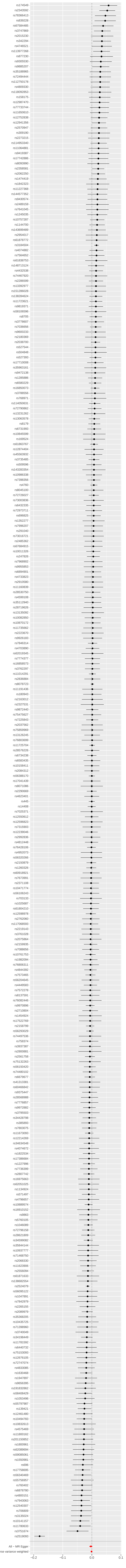

Supplementary Figure 48: Forest plot of the causal effects of single nucleotide polymorphisms associated with monocyte percentage on venous thromboembolism.The significance of red lines are MR results of MR-Egger test and IVW method.

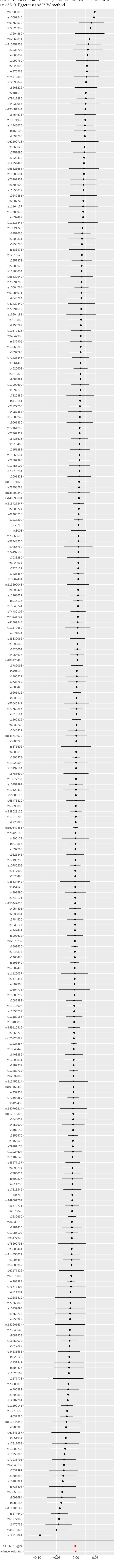

Supplementary Figure 49: Forest plot of the causal effects of single nucleotide polymorphisms associated with monocyte count on venous thromboembolism. The significance of red lines are MR results of MR-Egger test and IVW method.

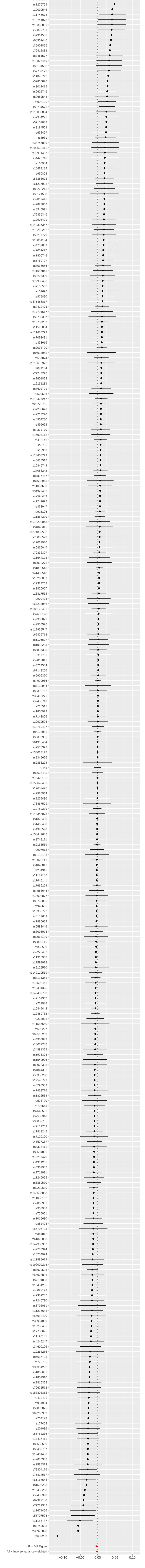

Supplementary Figure 50: Forest plot of the causal effects of single nucleotide polymorphisms associated with red blood cell distribution width on venous thromboembolism. The significance of red lines are MR results of MR-Egger test and IVW method.

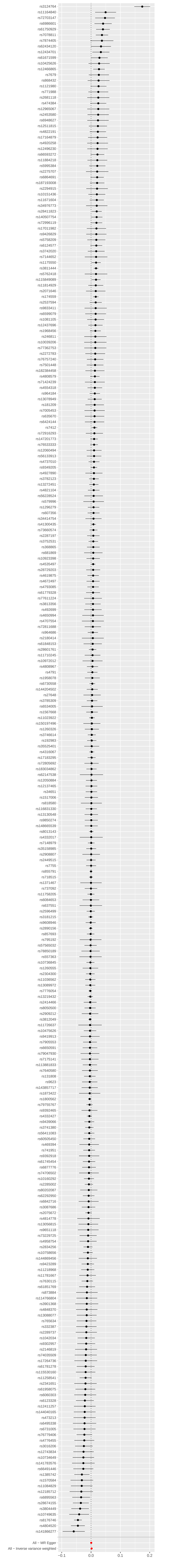

Supplementary Figure 51: Forest plot of the causal effects of single nucleotide polymorphisms associated with red blood cell count on venous thromboembolism.The significance of red lines are MR results of MR-Egger test and IVW method.

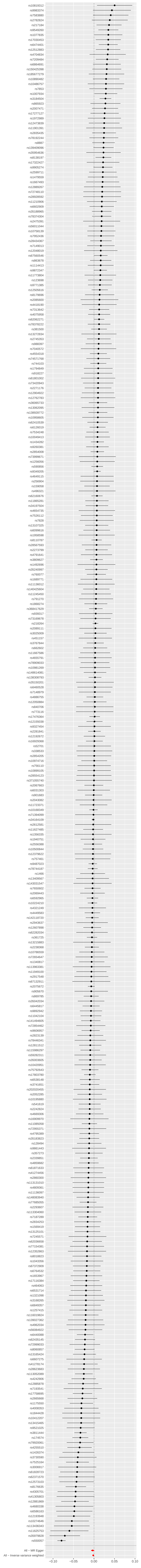

Supplementary Figure 52: Forest plot of the causal effects of single nucleotide polymorphisms associated with lymphocyte percentage on venous thromboembolism.The significance of red lines are MR results of MR-Egger test and IVW method.

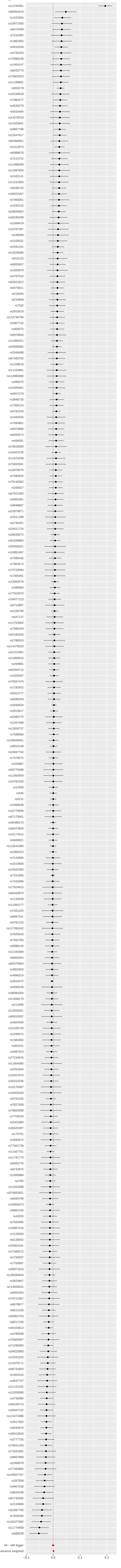

Supplementary Figure 53: Forest plot of the causal effects of single nucleotide polymorphisms associated with lymphocyte count on venous thromboembolism.The significance of red lines are MR results of MR-Egger test and IVW method.

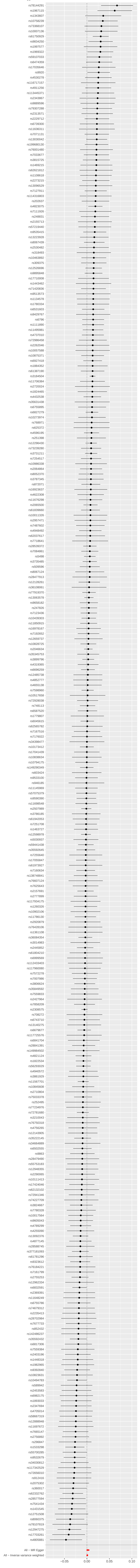

Supplementary Figure 54: Forest plot of the causal effects of single nucleotide polymorphisms associated with mean corpuscular volume on venous thromboembolism.The significance of red lines are MR results of MR-Egger test and IVW method.

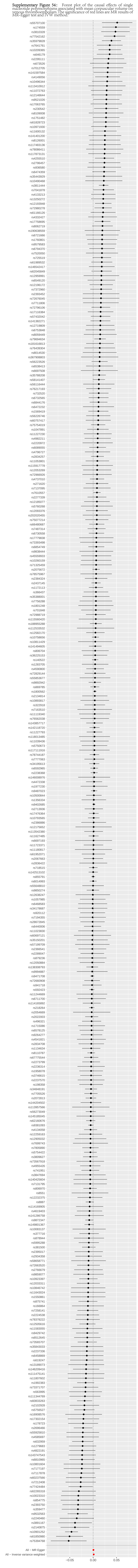

Supplementary Figure 55: Forest plot of the causal effects of single nucleotide polymorphisms associated with mean corpuscular haemoglobin concentration on venous thrmboembolism.The significance of red lines are MR results of MR-Egger test and IVW method.

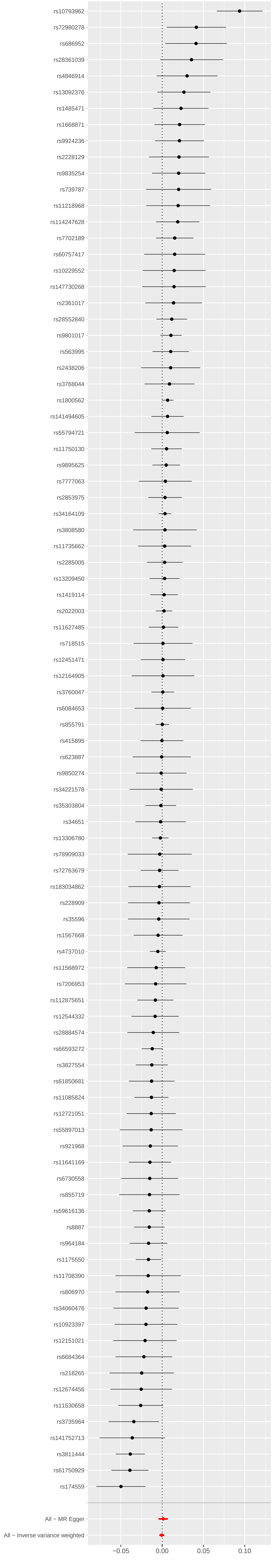

Supplementary Figure 56: Forest plot of the causal effects of single nucleotide polymorphisms associated with mean corpuscular volume of reticulocyte on venous thromboembolism.The significance of red lines are MR results of MR-Egger test and IVW method.

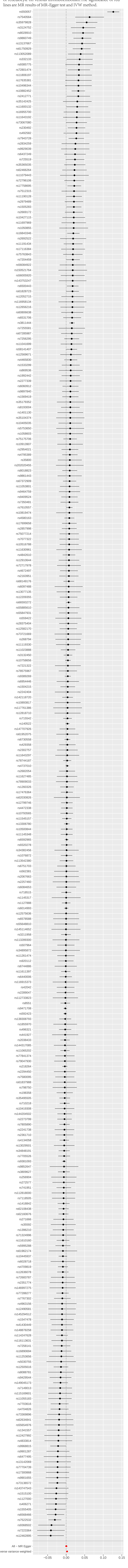

Supplementary Figure 57: Forest plot of the causal effects of single nucleotide polymorphisms associated with mean platelet volume on venous thromboembolism. The significance of red lines are MR results of MR-Egger test and IVW method.

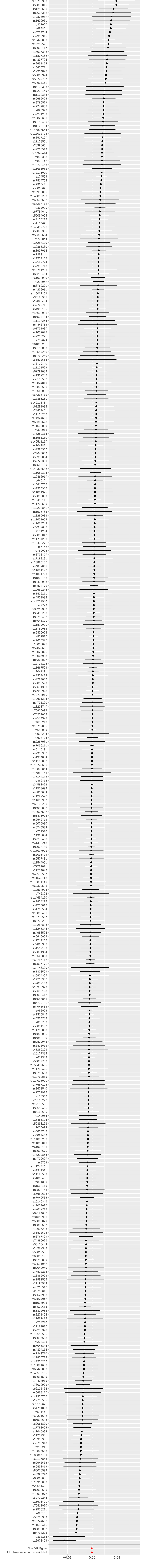

Supplementary Figure 58: Forest plot of the causal effects of single nucleotide polymorphisms associated with Basophil percentage of granulocytes on venous thromboembolism.The significance of red lines are MR results of MR-Egger test and IVW method.

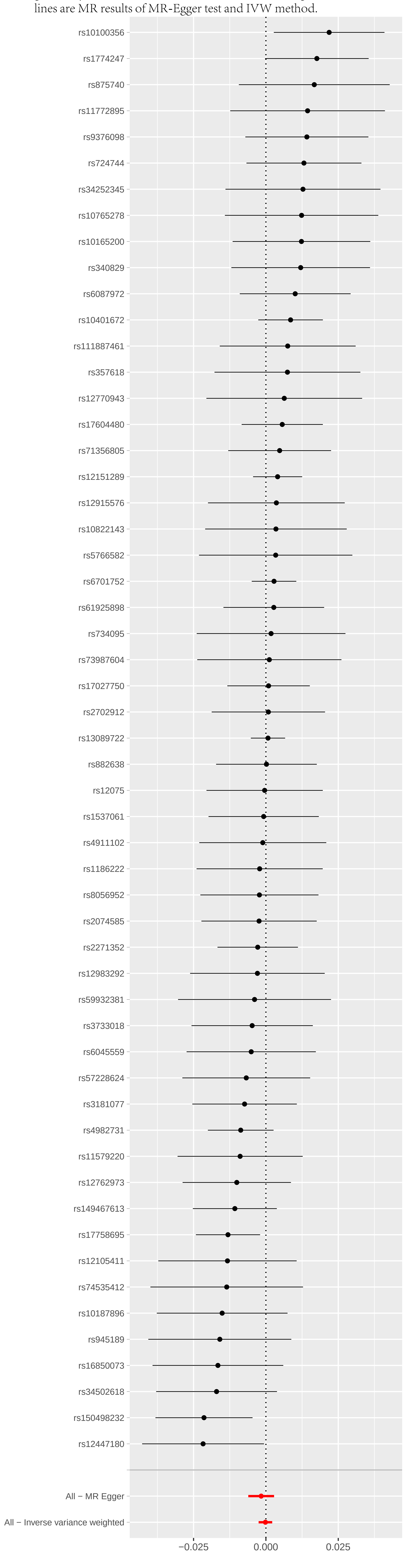

Supplementary Figure 59: Forest plot of the causal effects of single nucleotide polymorphisms associated with sum basophil neutrophil counts on venous thromboembolism. The significance of red lines are MR results of MR-Egger test and IVW method.

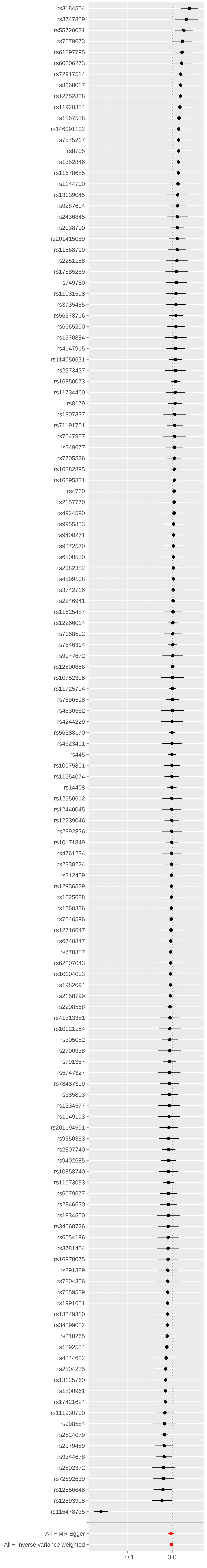

Supplementary Figure 60: Forest plot of the causal effects of single nucleotide polymorphisms associated with eosinophil percentage of granulocytes on venous thromboembolism.The significance of red lines are MR results of MR-Egger test and IVW method.

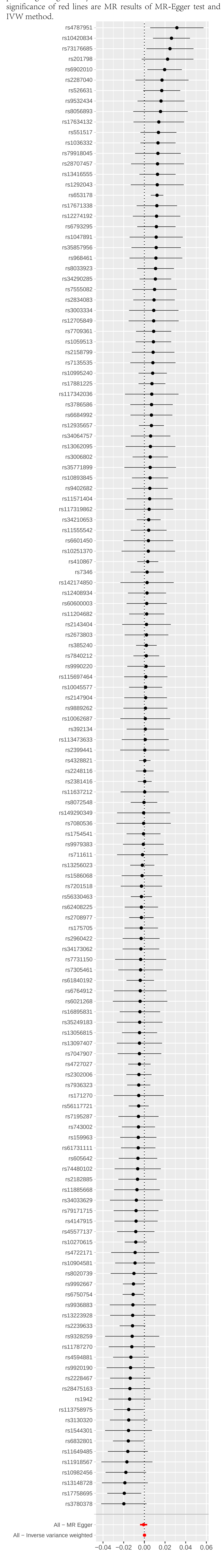

Supplementary Figure 61: Forest plot of the causal effects of single nucleotide polymorphisms associated with eosinophil counts on venous thromboembolism.The significance of red lines are MR results of MR-Egger test and IVW method.

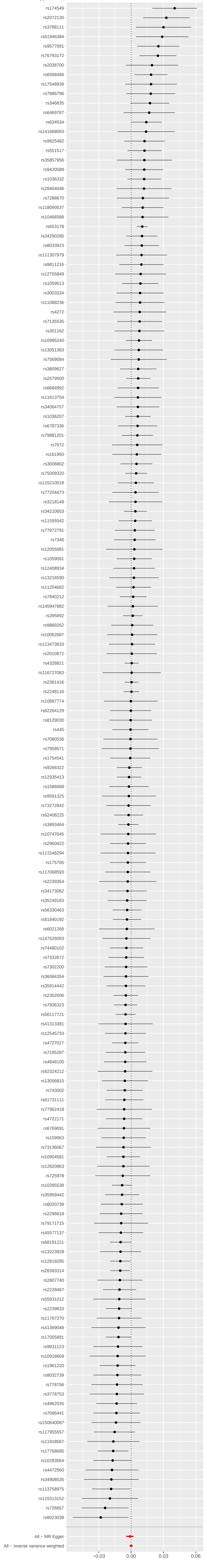

Supplementary Figure 62: Forest plot of the causal effects of single nucleotide polymorphisms associated with reticulocyte percentage on venous thromboembolism.The significance of red lines are MR results of MR-Egger test and IVW method.

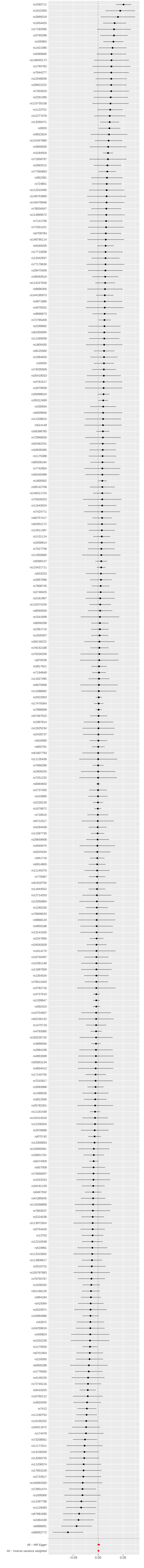

Supplementary Figure 63: Forest plot of the causal effects of single nucleotide polymorphisms associated with reticulocyte count on venous thromboembolism.The significance of red lines are MR results of MR-Egger test and IVW method.

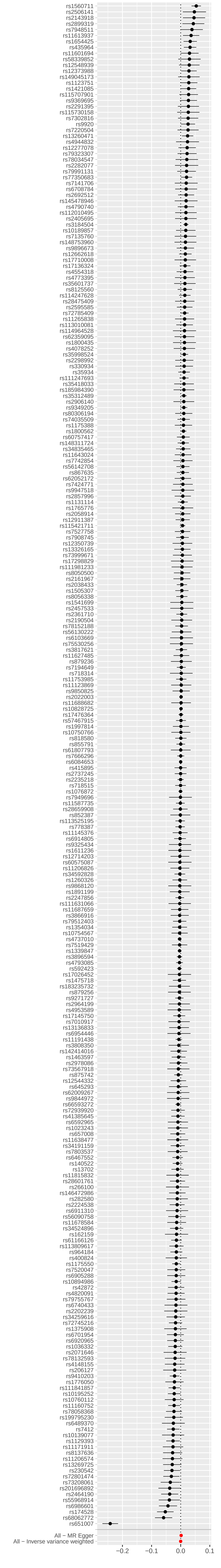

Supplementary Figure 64: Forest plot of the causal effects of single nucleotide polymorphisms associated with haemoglobin concentration on venous thromboembolism. The significance of red lines are MR results of MR-Egger test and IVW method.

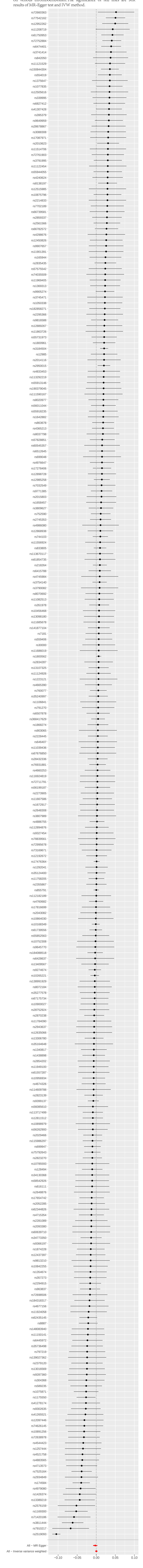

Supplementary Figure 65: Forest plot of the causal effects of single nucleotide polymorphisms associated with hematocrit on venous thromboembolism.The significance of red lines are MR results of MR-Egger test and IVW method.

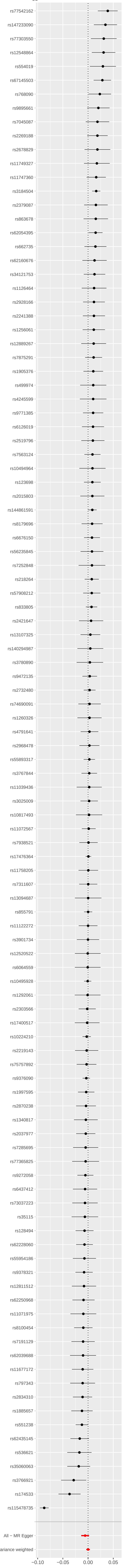

Supplementary Figure 66: Forest plot of the causal effects of single nucleotide polymorphisms associated with platelet distribution width on venous thromboembolism.The significance of red lines are MR results of MR-Egger test and IVW method.

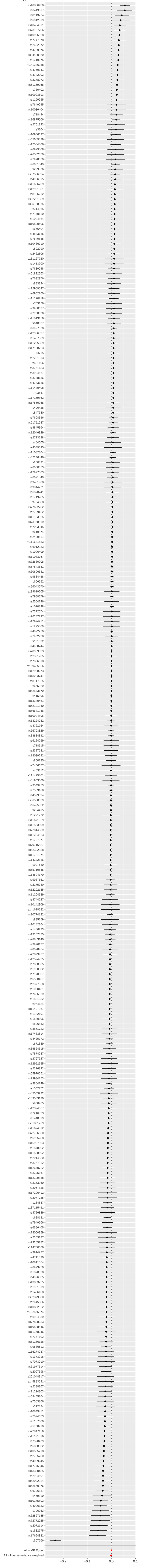

Supplementary Figure 67: Forest plot of the causal effects of single nucleotide polymorphisms associated with platelet count on venous thromboembolism.The significance of red lines are MR results of MR-Egger test and IVW method.

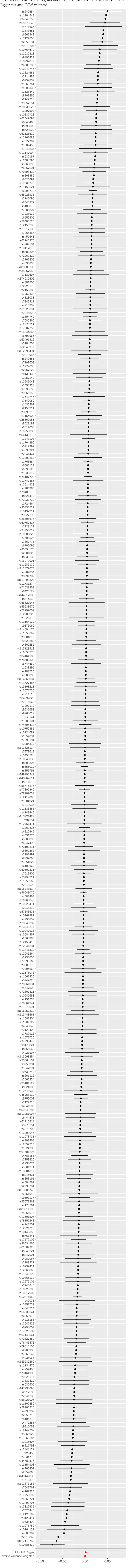

Supplementary Figure 68: Forest plot of the causal effects of single nucleotide polymorphisms associated with neutrophil percentage of granulocytes on venous thromboembolism.The significance of red lines are MR results of MR-Egger test and IVW method.

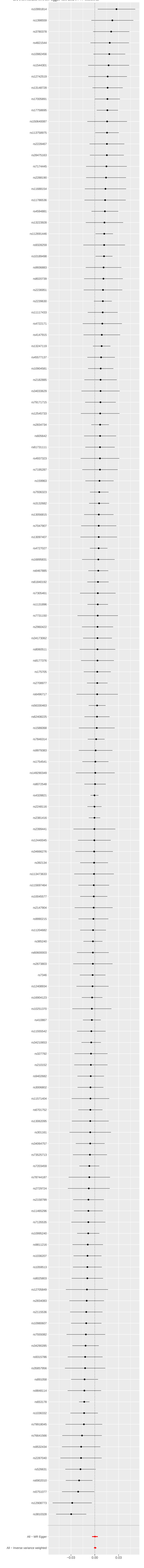

Supplementary Figure 69: Forest plot of the causal effects of single nucleotide polymorphisms associated with neutrophil count on venous thromboembolism.The significance of red lines are MR results of MR-Egger test and IVW method.

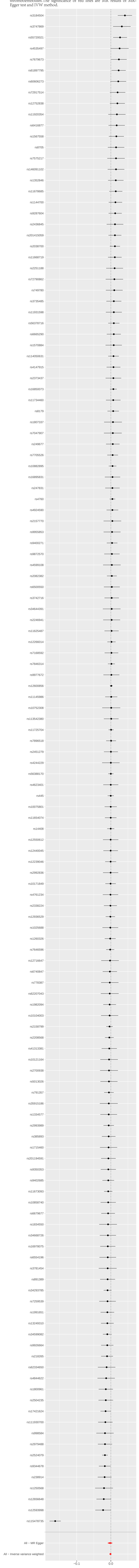

Supplementary Figure 70: Scatter plots of genetic associations with white blood cell count against the genetic associations with venous thromboembolism. The slopes of each line represent the causal association for each method.

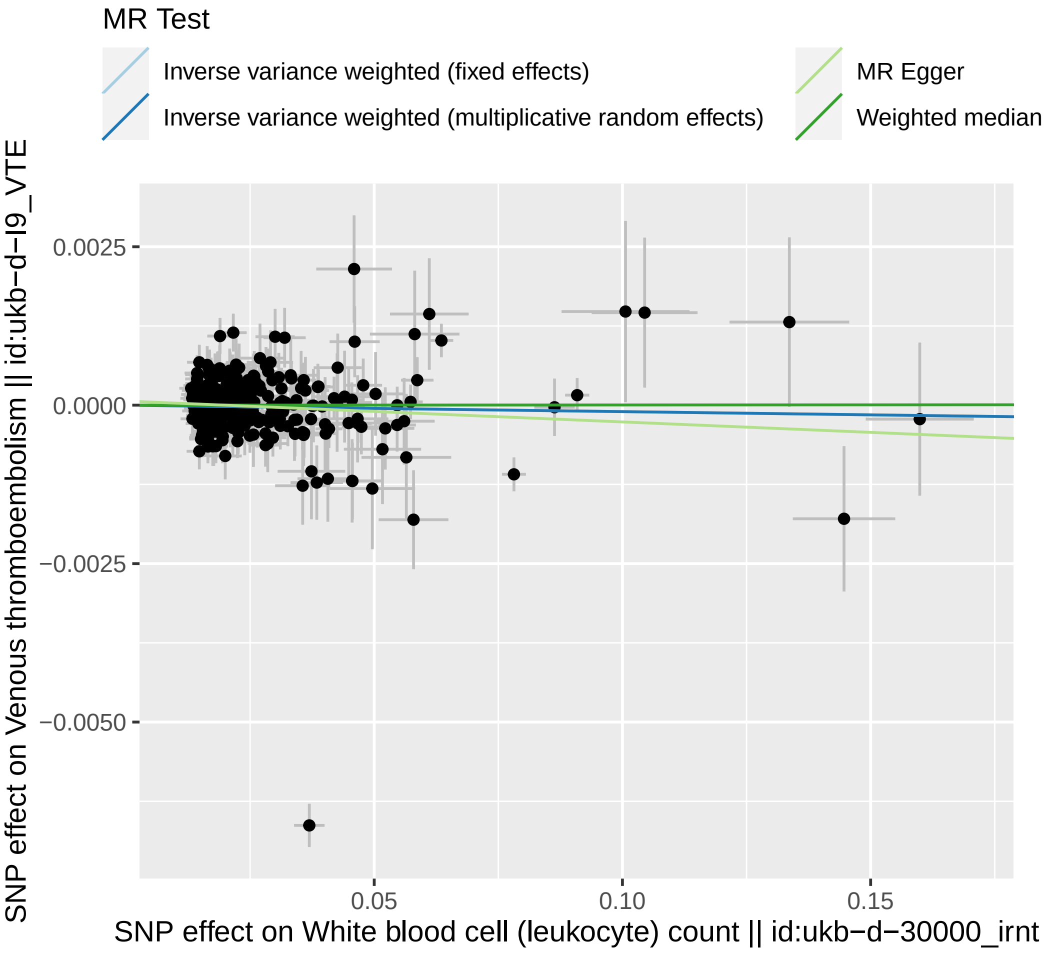

Supplementary Figure 71: Scatter plots of genetic associations with monocyte percentage against the genetic associations with venous thromboembolism. The slopes of each line represent the causal association for each method.

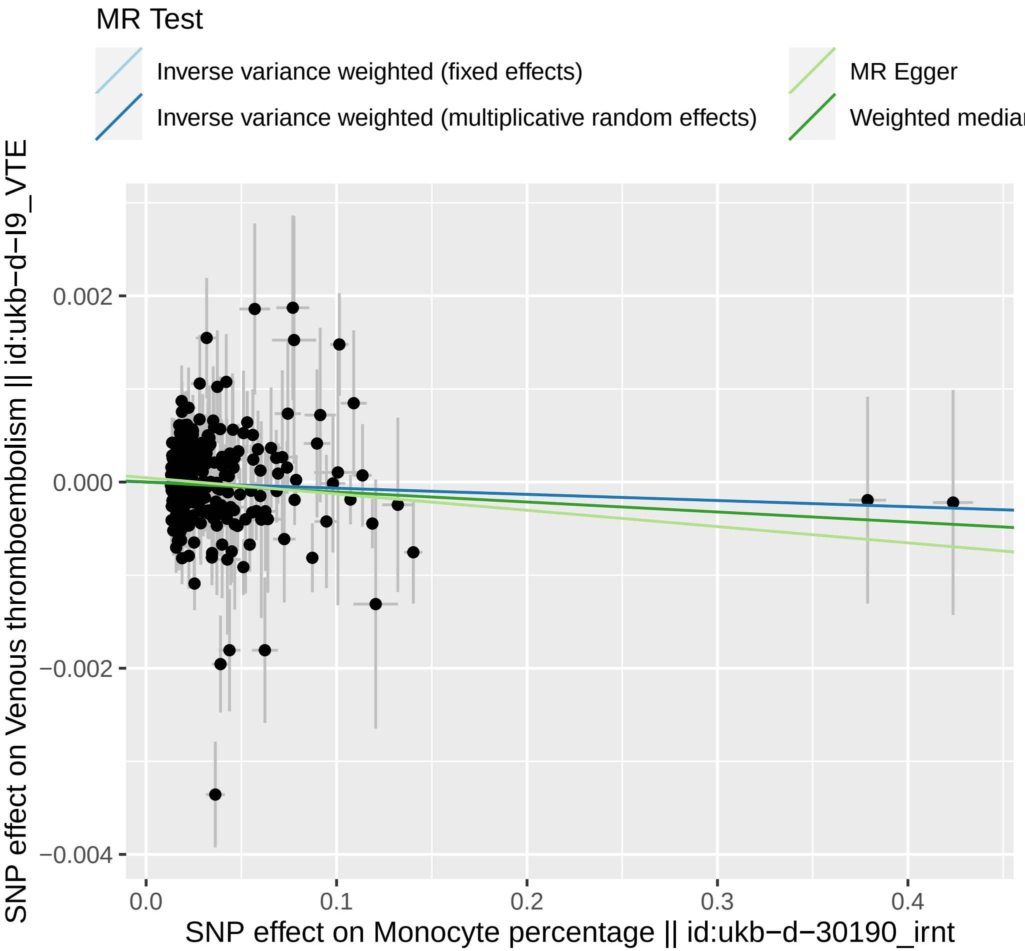

Supplementary Figure 72: Scatter plots of genetic associations with monocyte count against the genetic associations with venous thromboembolism. The slopes of each line represent the causal association for each method.

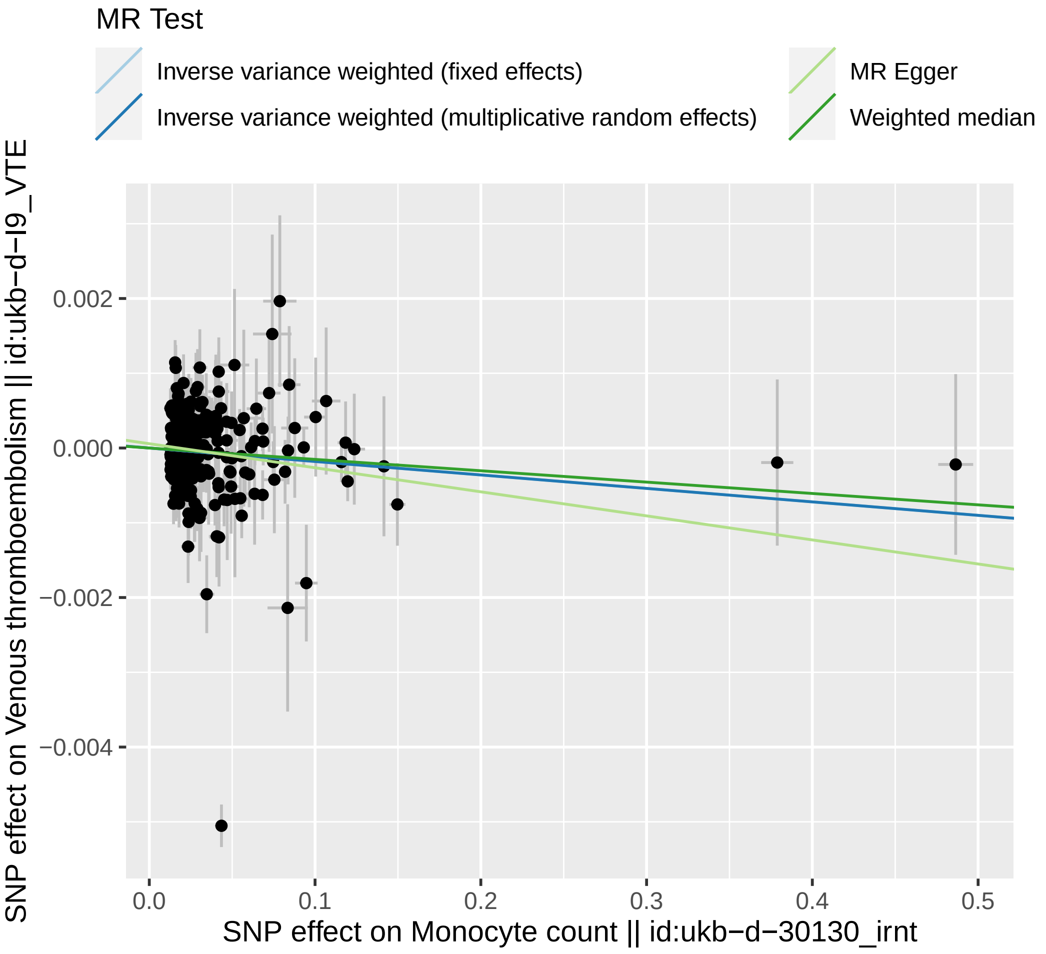

Supplementary Figure 73: Scatter plots of genetic associations with red blood cell distribution width against the genetic associations with venous thromboembolism. The slopes of each line represent the causal association for each method.

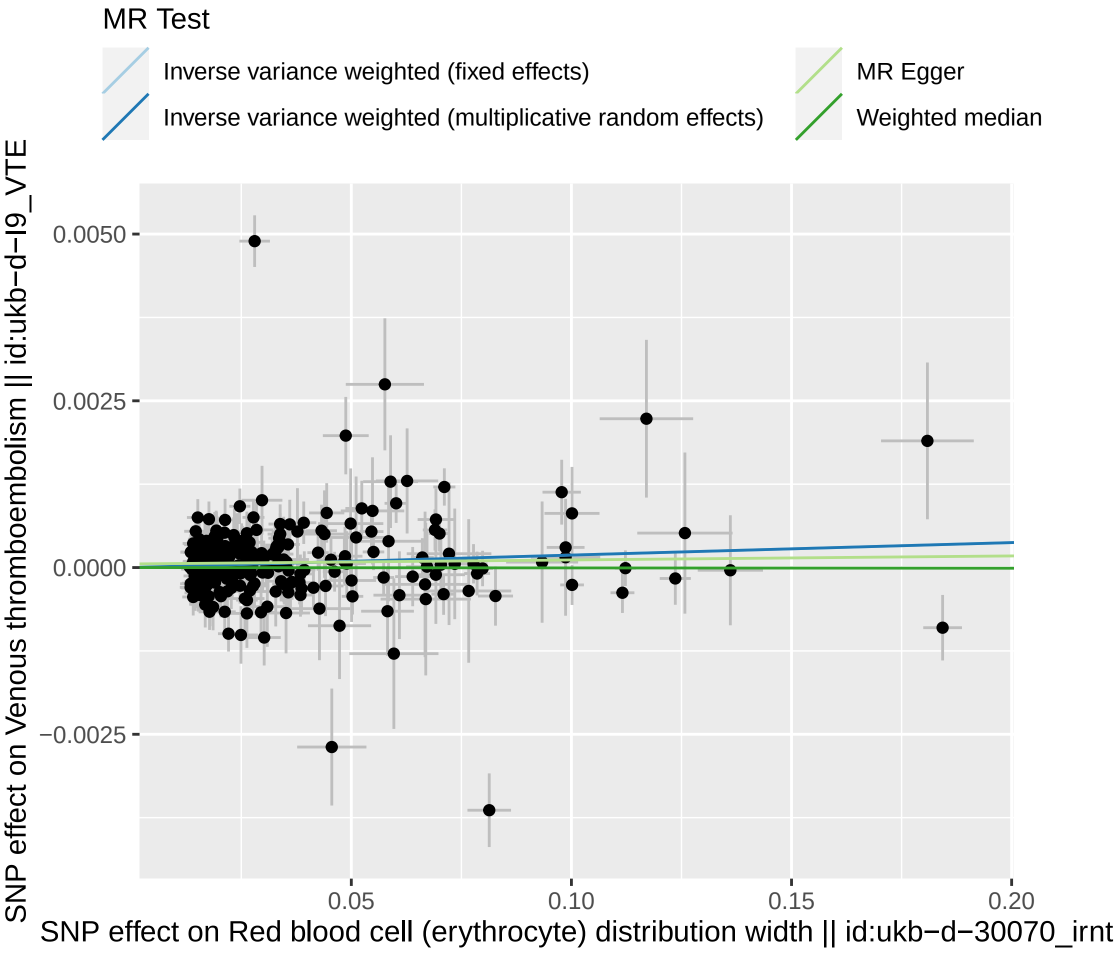

Supplementary Figure 74: Scatter plots of genetic associations with red blood cell count against the genetic associations with venous thromboembolism. The slopes of each line represent the causal association for each method.

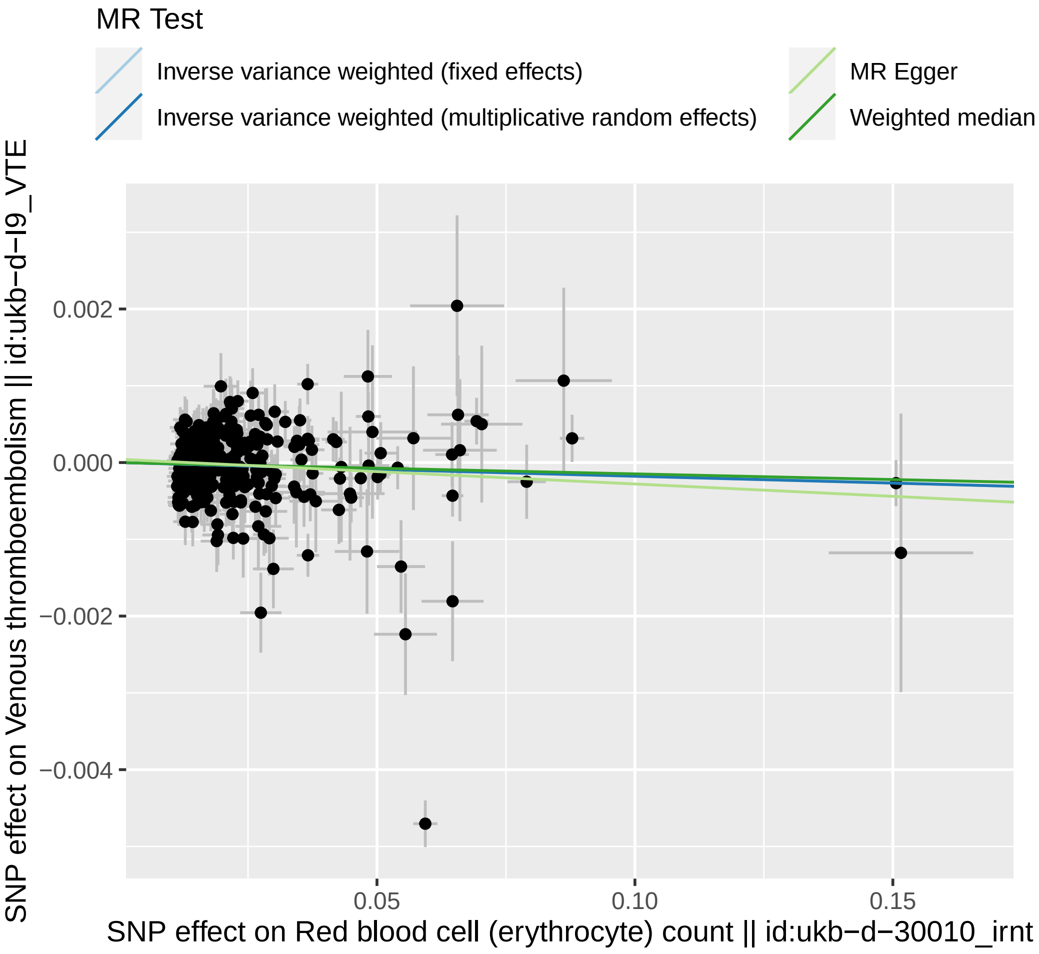

Supplementary Figure 75: Scatter plots of genetic associations with lymphocyte percentage against the genetic associations with venous thromboembolism. The slopes of each line represent the causal association for each method.

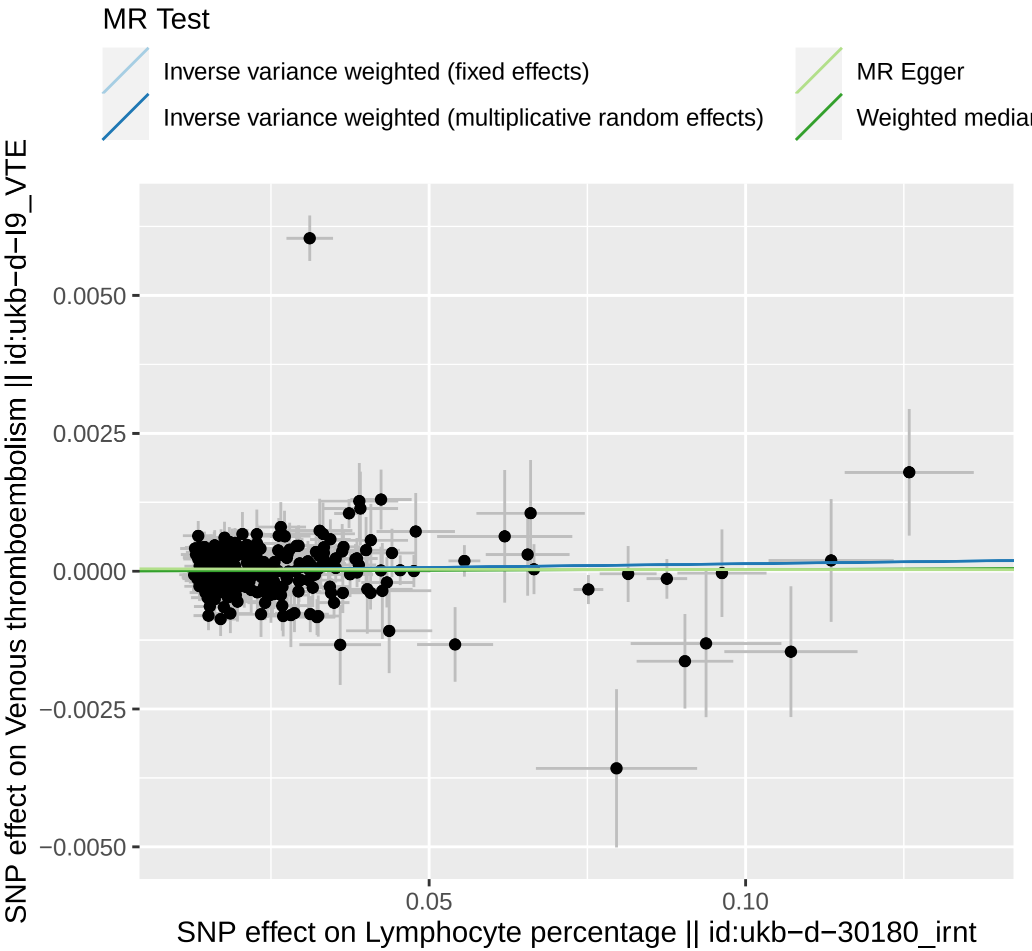

Supplementary Figure 76: Scatter plots of genetic associations with lymphocyte count against the genetic associations with venous thromboembolism. The slopes of each line represent the causal association for each method.

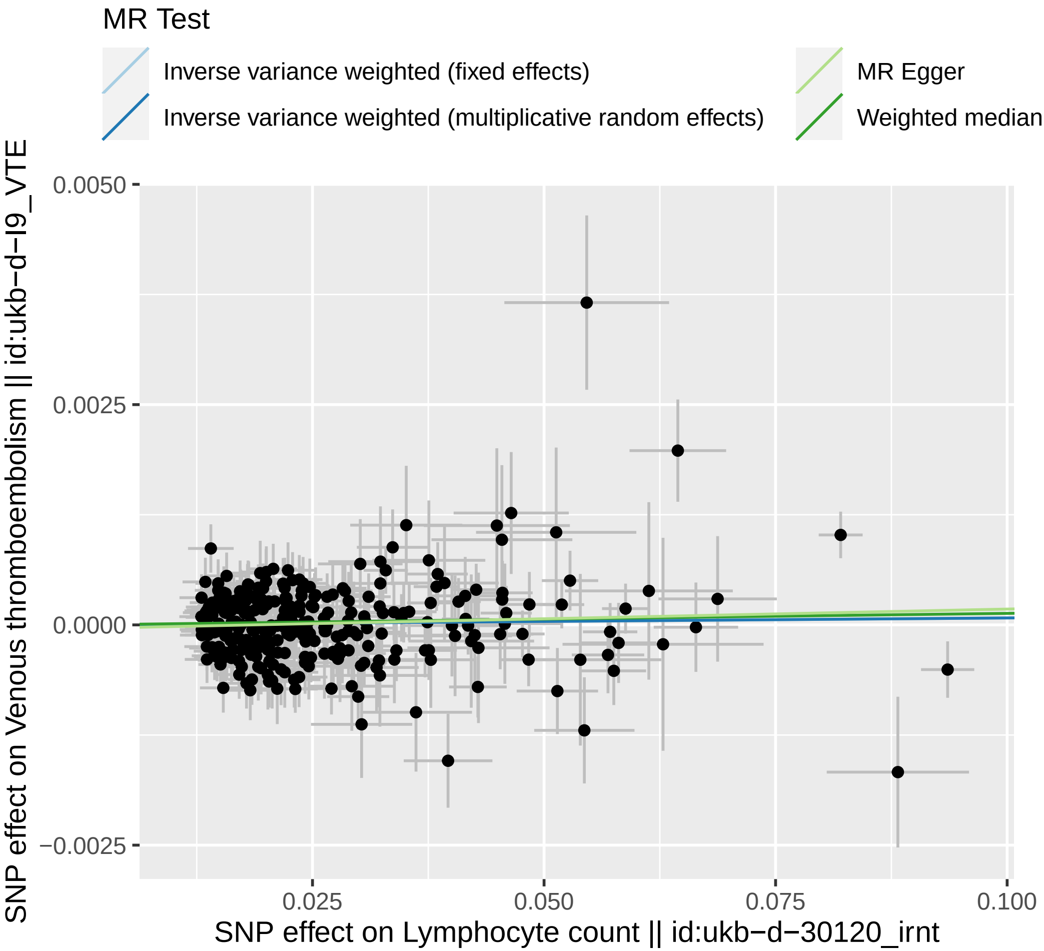

Supplementary Figure 77: Scatter plots of genetic associations with mean corpuscular volume against the genetic associations with venous thromboembolism. The slopes of each line represent the causal association for each method.

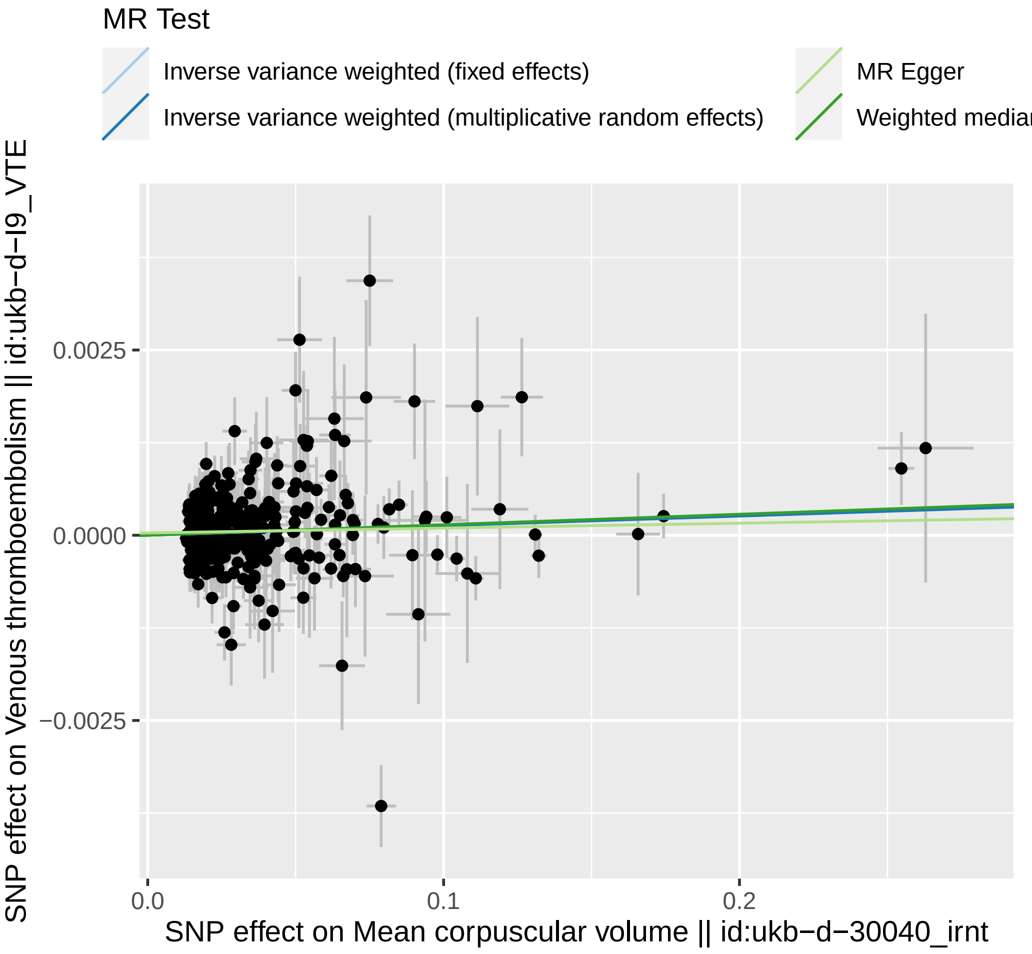

Supplementary Figure 78: Scatter plots of genetic associations with mean corpuscular haemoglobin concentration against the genetic associations with venous thromboembolism. The slopes of each line represent the causal association for each method.

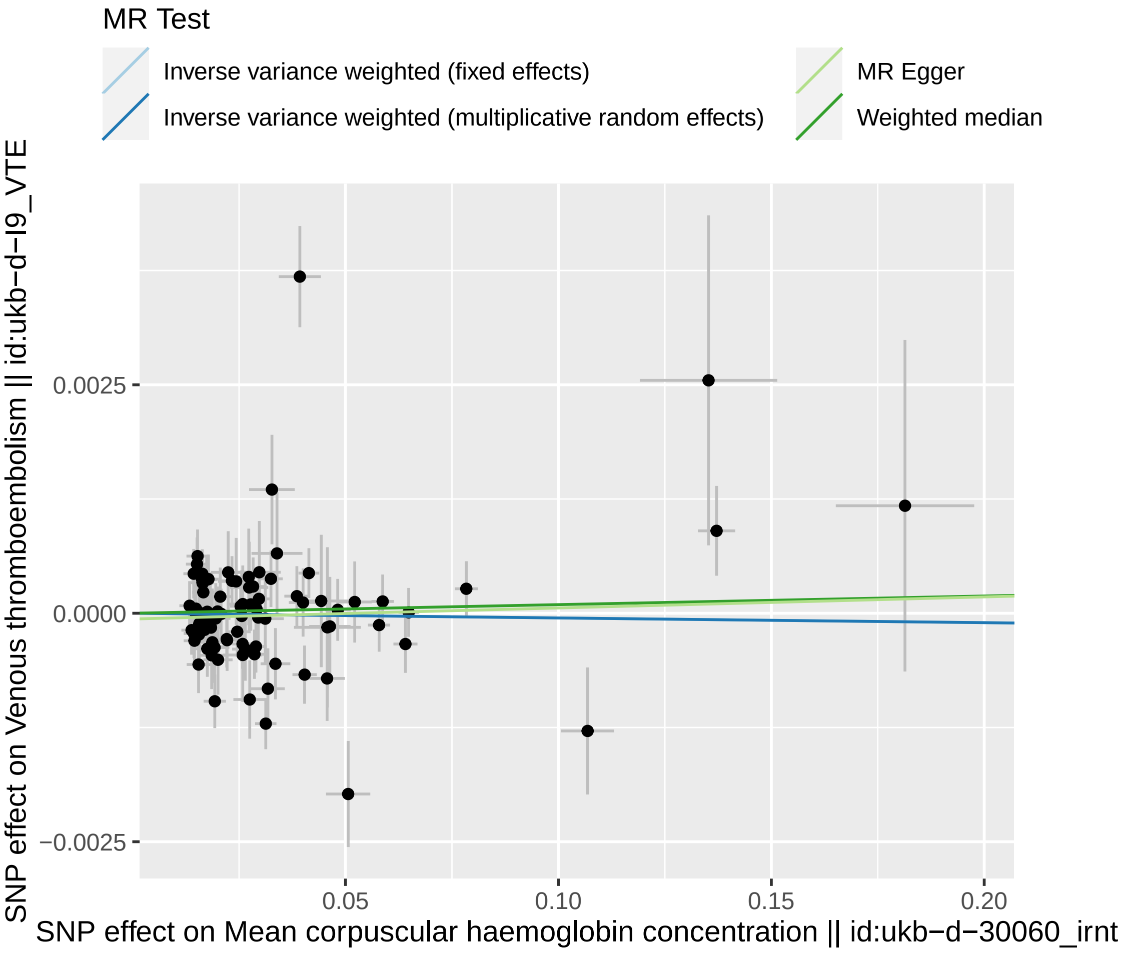

Supplementary Figure 79: Scatter plots of genetic associations with mean corpuscular volume of reticulocyte against the genetic associations with venous thromboembolism. The slopes of each line represent the causal association for each method.

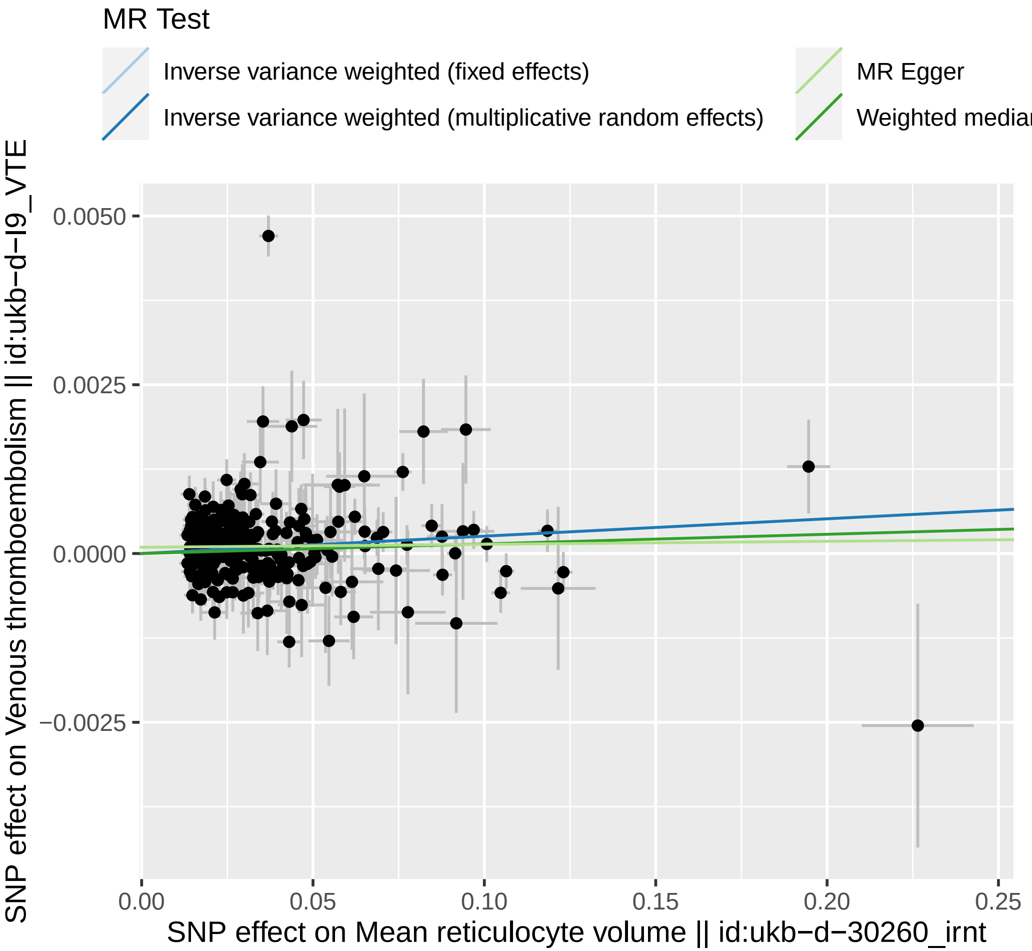

Supplementary Figure 80: Scatter plots of genetic associations with mean platelet volume against the genetic associations with venous thromboembolism. The slopes of each line represent the causal association for each method.

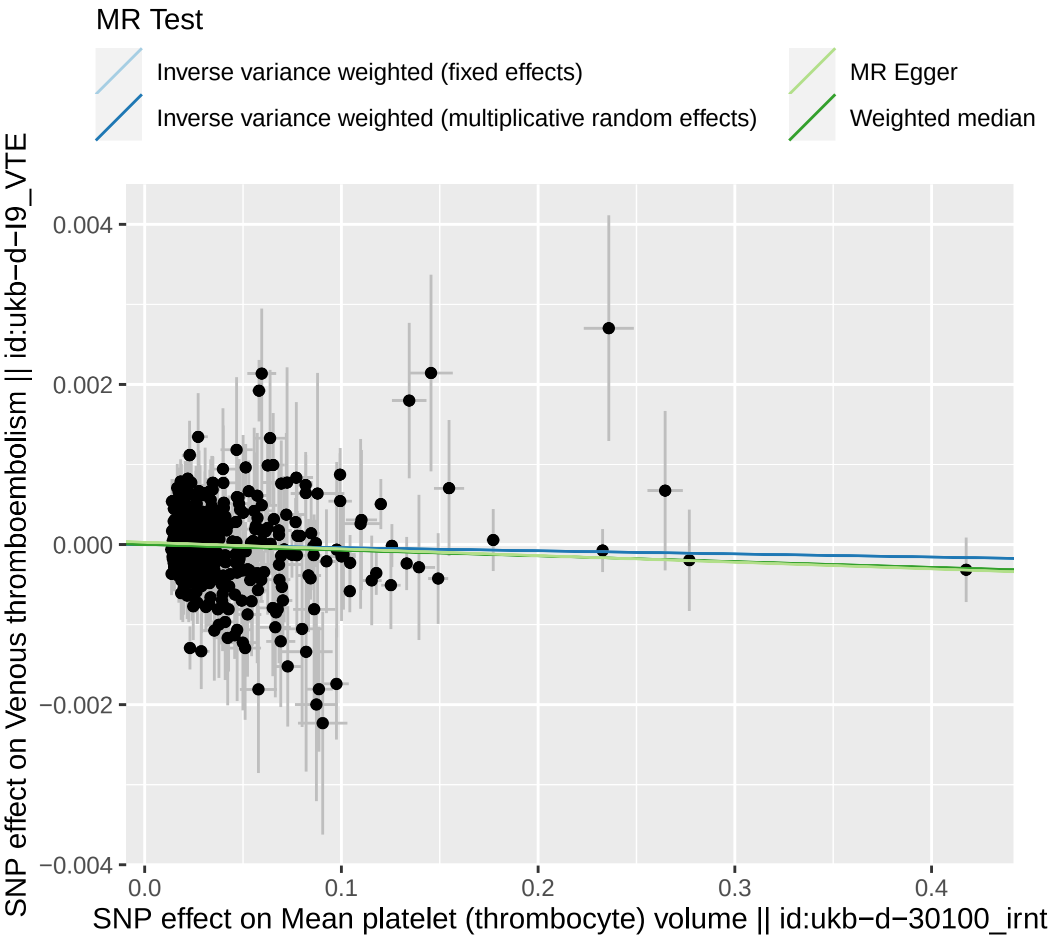

Supplementary Figure 81: Scatter plots of genetic associations with basophil percentage of granulocytes against the genetic associations with venous thromboembolism. The slopes of each line represent the causal association for each method.

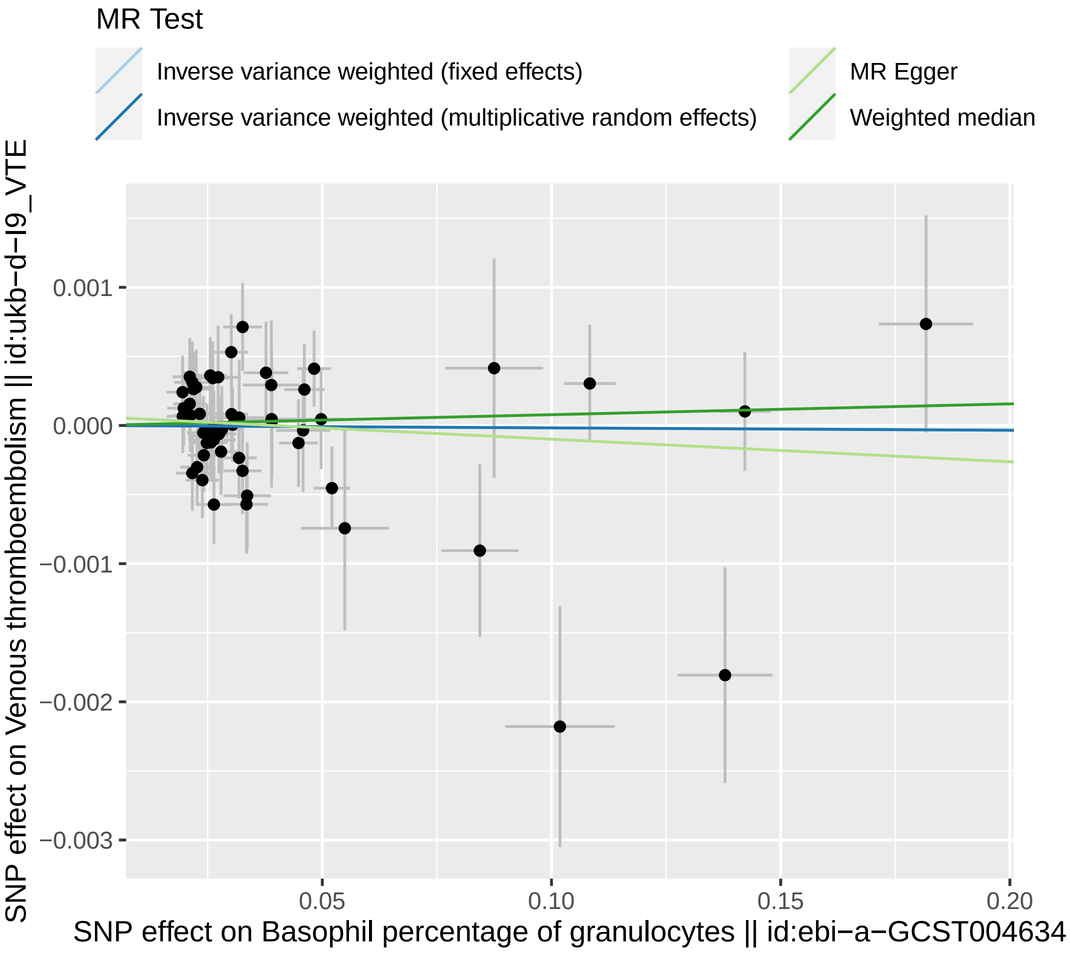

Supplementary Figure 82: Scatter plots of genetic associations with sum basophil neutrophil counts against the genetic associations with venous thromboembolism. The slopes of each line represent the causal association for each method.

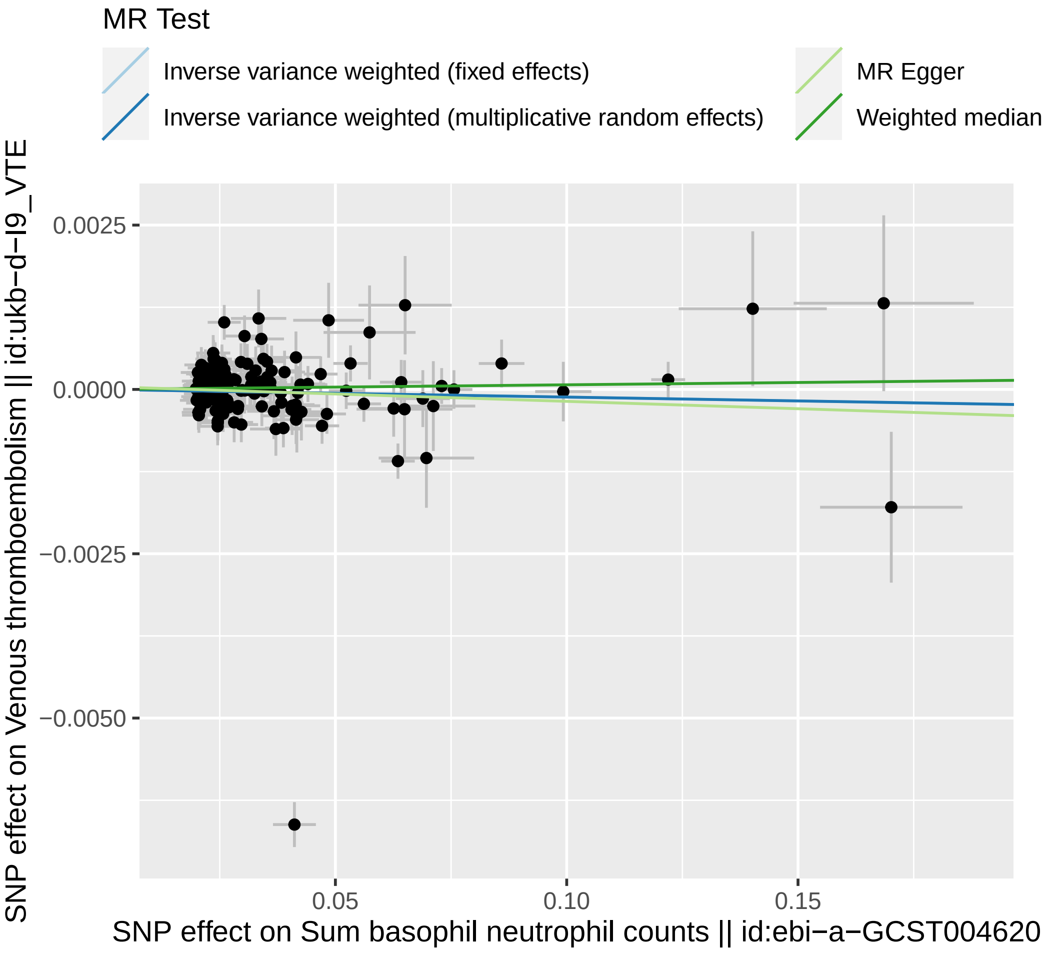

Supplementary Figure 83: Scatter plots of genetic associations with eosinophil percentage of granulocytes against the genetic associations with venous thromboembolism. The slopes of each line represent the causal association for each method.

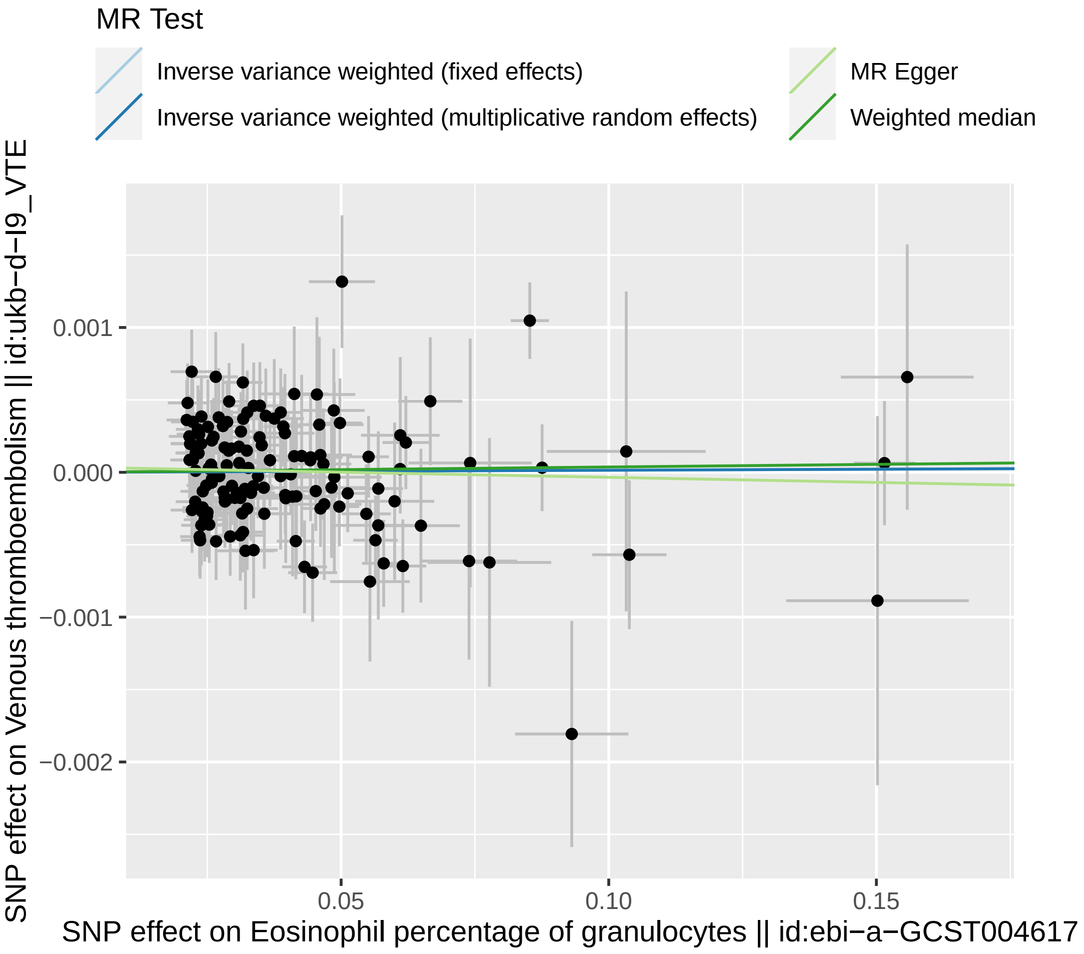

Supplementary Figure 84: Scatter plots of genetic associations with eosinophil counts against the genetic associations with venous thromboembolism. The slopes of each line represent the causal association for each method.

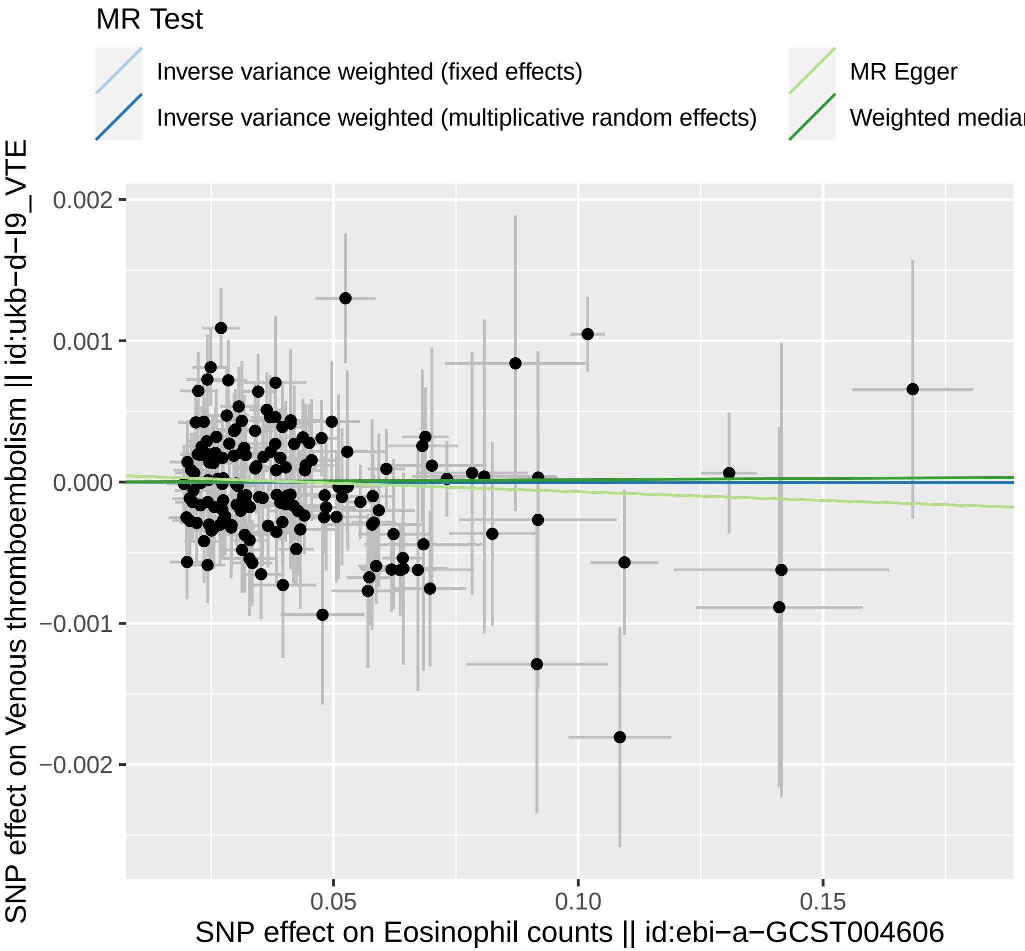

Supplementary Figure 85: Scatter plots of genetic associations with reticulocyte percentage against the genetic associations with venous thromboembolism. The slopes of each line represent the causal association for each method.

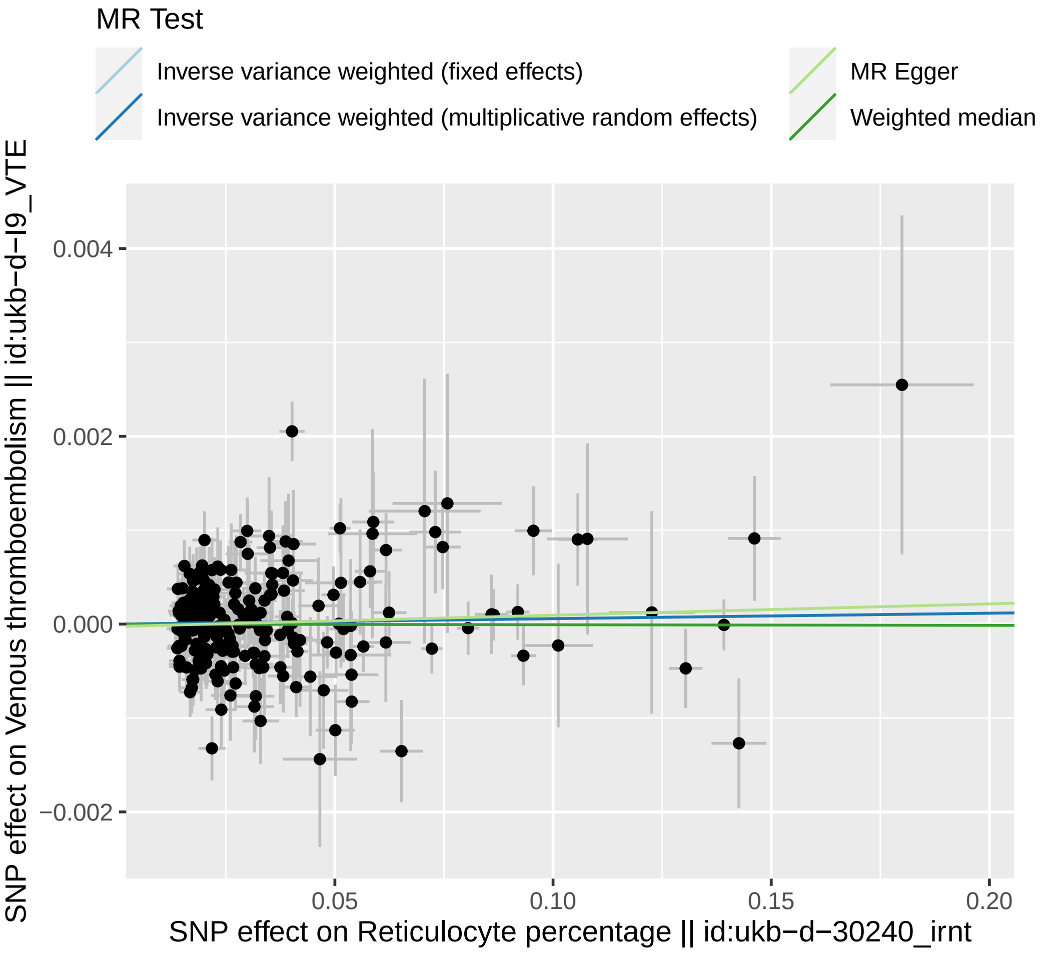

Supplementary Figure 86: Scatter plots of genetic associations with reticulocyte count against the genetic associations with venous thromboembolism. The slopes of each line represent the causal association for each method.

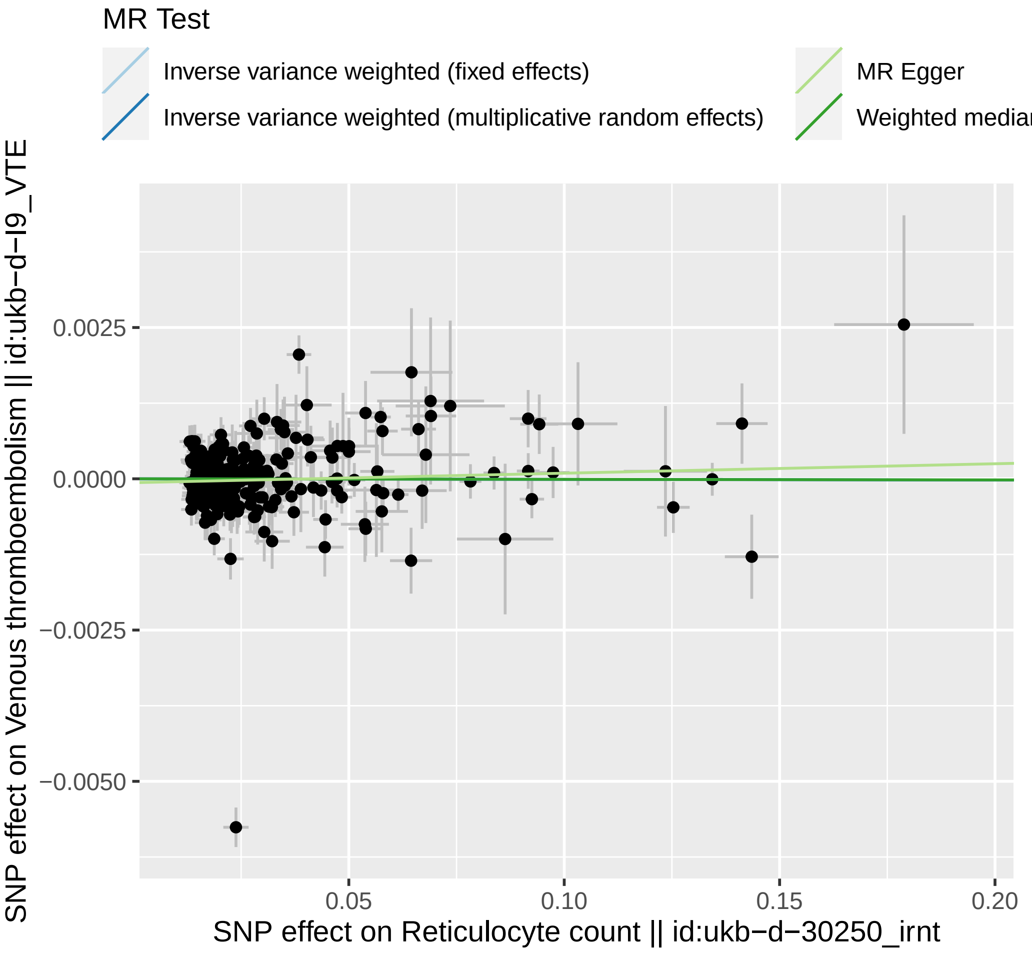

Supplementary Figure 87: Scatter plots of genetic associations with haemoglobin concentration against the genetic associations with venous thromboembolism. The slopes of each line represent the causal association for each method.

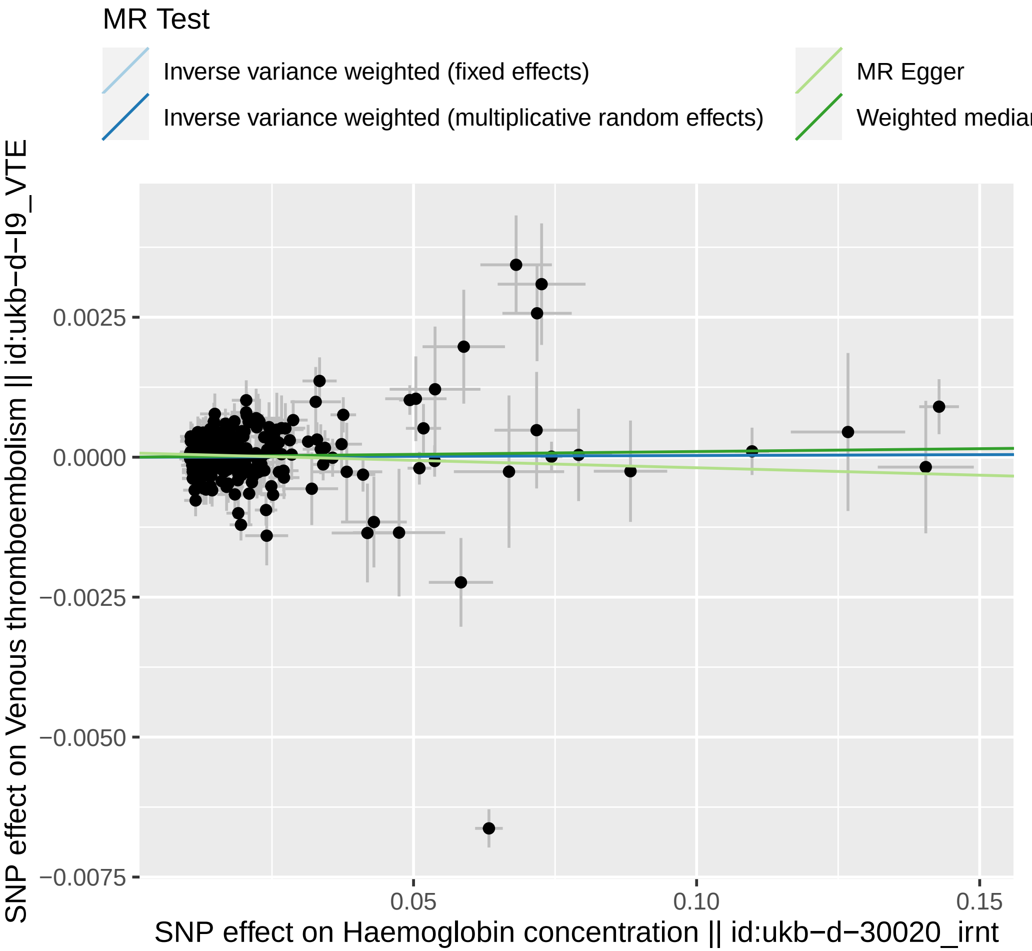

Supplementary Figure 88: Scatter plots of genetic associations with hematocrit against the genetic associations with venous thromboembolism. The slopes of each line represent the causal association for each method.

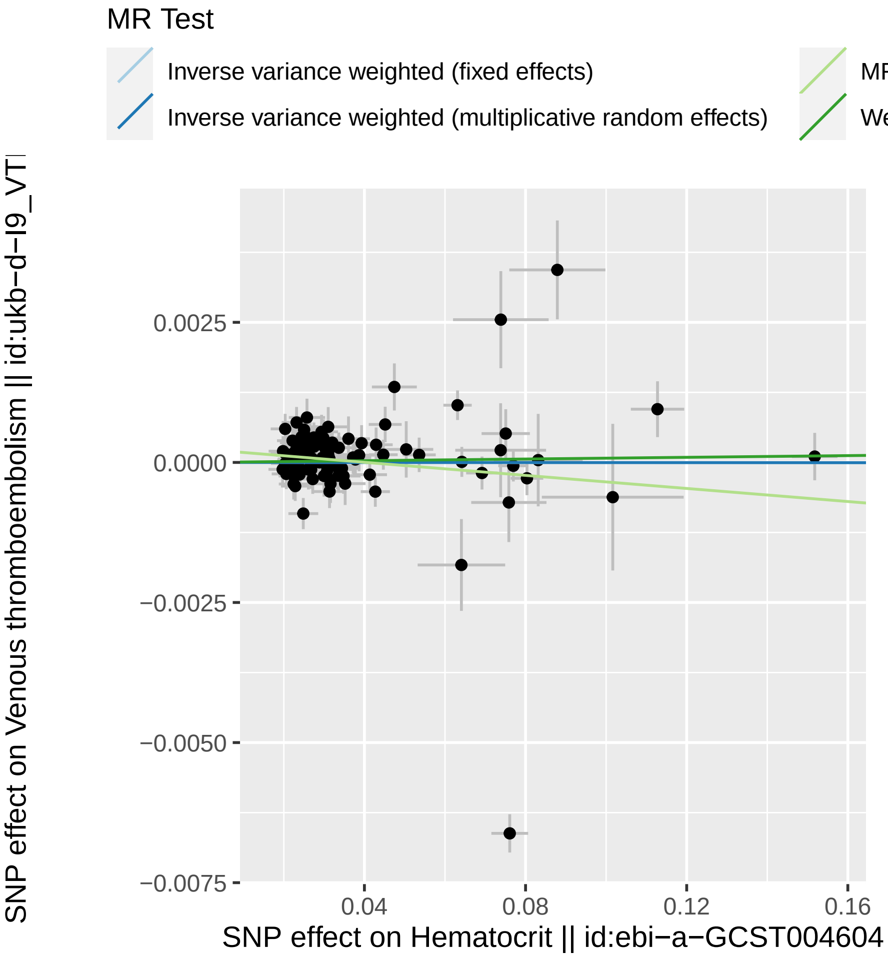

Supplementary Figure 89: Scatter plots of genetic associations with platelet distribution width against the genetic associations with venous thromboembolism. The slopes of each line represent the causal association for each method.

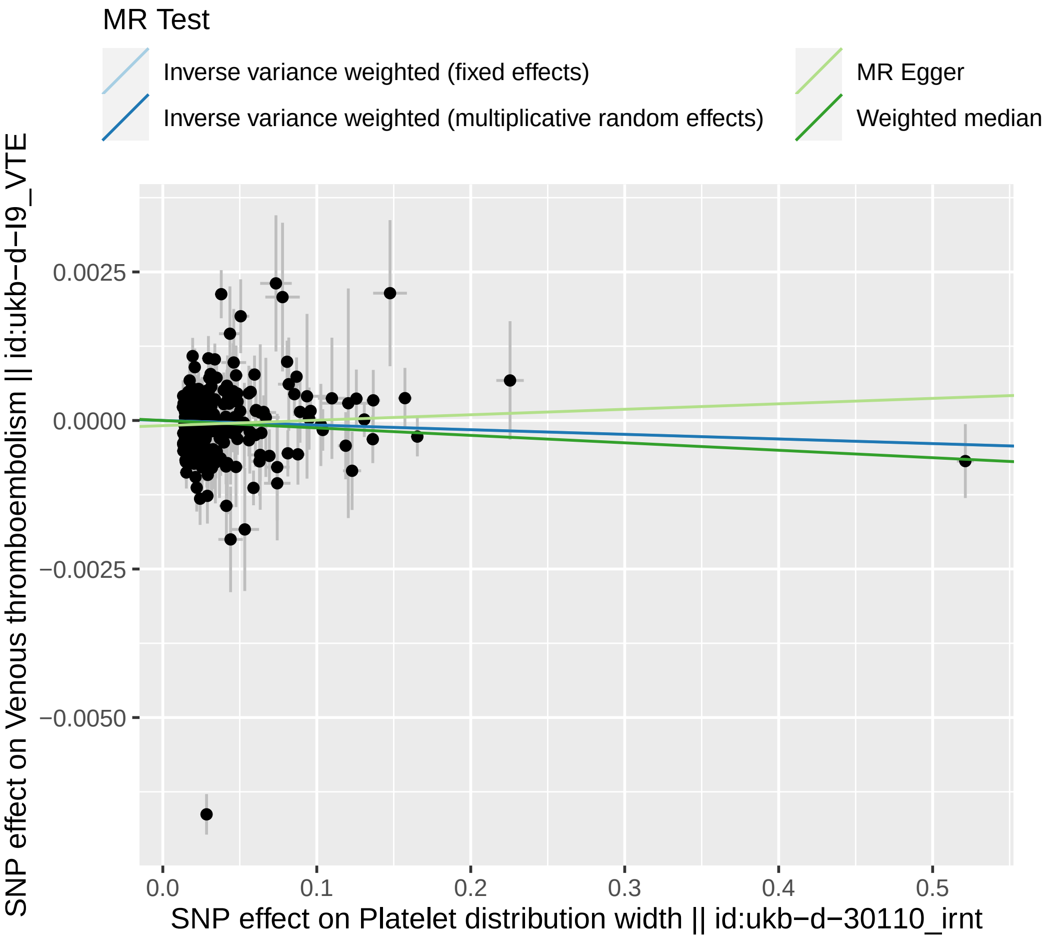

Supplementary Figure 90: Scatter plots of genetic associations with platelet count against the genetic associations with venous thromboembolism. The slopes of each line represent the causal association for each method.

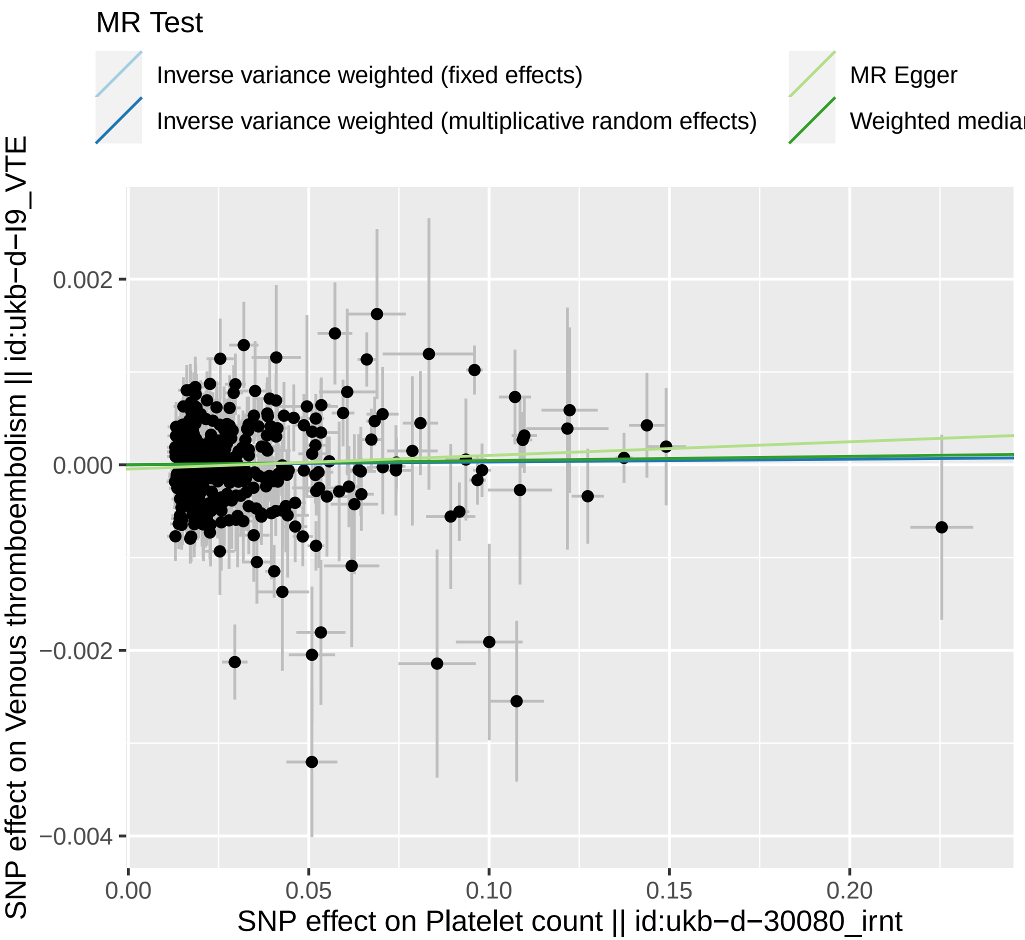

Supplementary Figure 91: Scatter plots of genetic associations with neutrophil percentage of granulocytes against the genetic associations with venous thromboembolism. The slopes of each line represent the causal association for each method.

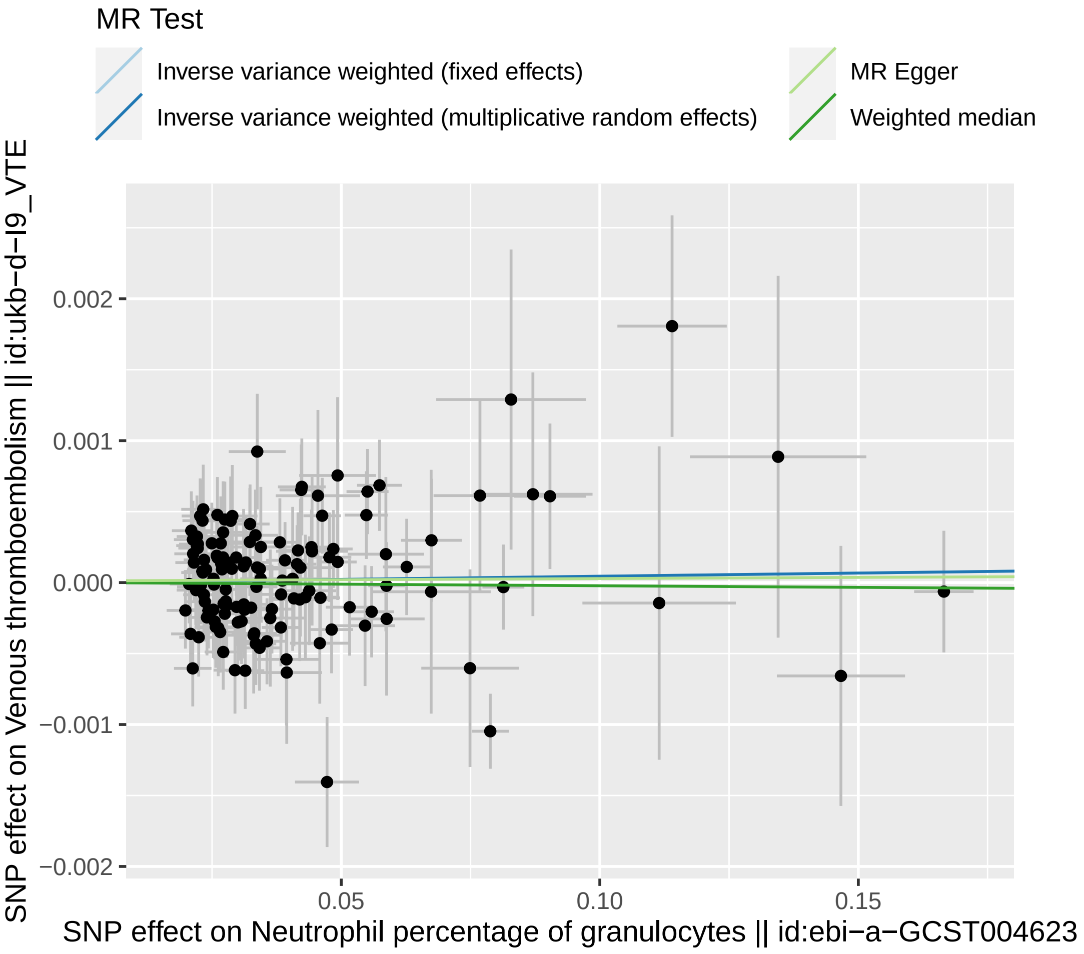

Supplementary Figure 92: Scatter plots of genetic associations with neutrophil count against the genetic associations with venous thromboembolism. The slopes of each line represent the causal association for each method.

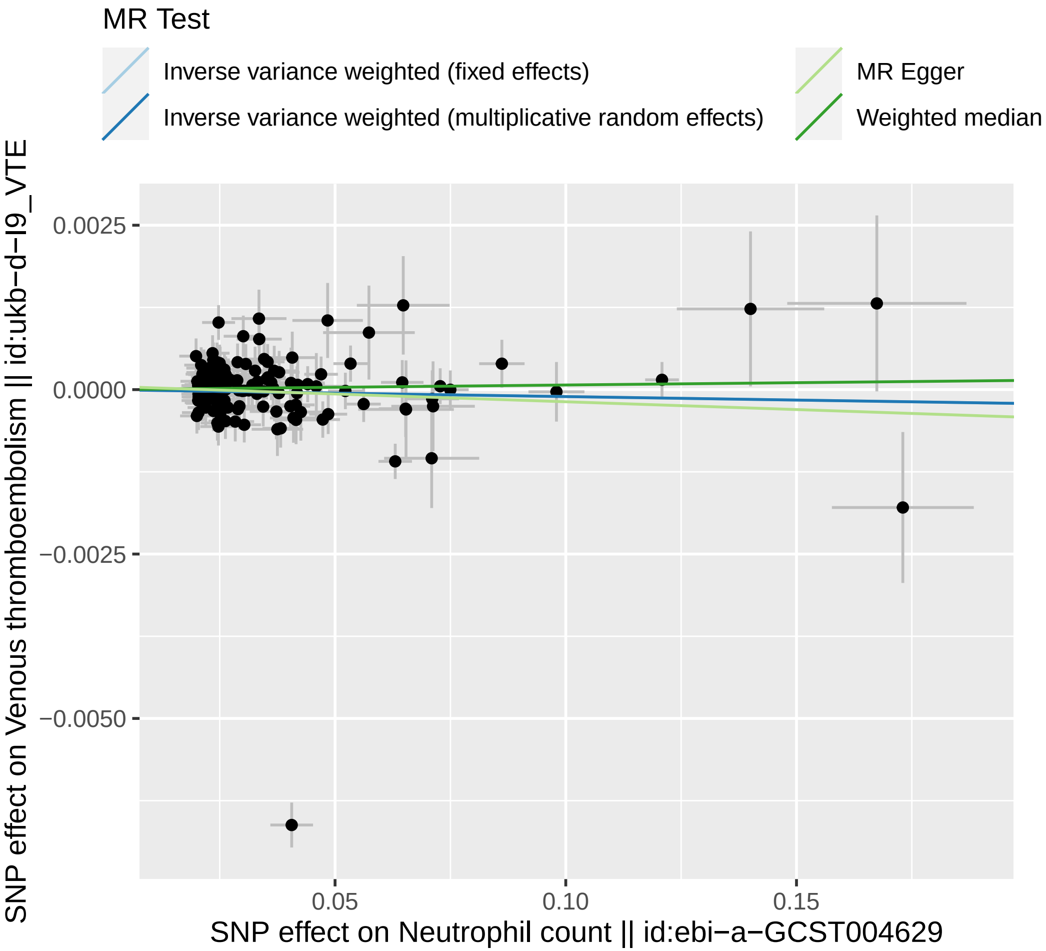

Supplement: Supplementary Figures 1–23 — Funnel plot showing results of heterogeneity test. [file Data_Sheet_1.pdf]
